# Noncanonical mTORC1-TFEB activation promotes hepatocyte plasticity and high-grade malignancy in hepatocellular carcinoma

**DOI:** 10.1172/JCI206334

**Published:** 2026-08-06

**Authors:** Chen Zhang, Xiaojuan Chao, Sha Neisha Williams, Xiaoli Wei, Anthony M. DiGirolamo, Alisha Bajracharya, Lichun Ma, Ming Huang, Nicholas Dunn, Wanqing Liu, Kaito Ueda, Masayuki Sugimoto, Andrea Ballabio, Hong-Min Ni, Wen-Xing Ding

**Affiliations:** 1 Department of Pharmacology, Toxicology and Therapeutics, University of Kansas Medical Center, Kansas City, Kansas, USA.; 2 Cancer Data Science Laboratory and; 3 Liver Cancer Program, Center for Cancer Research, National Cancer Institute, NIH, Bethesda, Maryland, USA.; 4 Department of Pharmaceutical Sciences, Eugene Applebaum College of Pharmacy and Health Sciences, and; 5 Department of Pharmacology, School of Medicine, Wayne State University, Detroit, Michigan, USA.; 6 Drug Delivery Research and Development Department, Nucleic Acid Medicine Business Division, Corporate Technology Sector, Nitto Denko Corporation, 1-1-2, Shimohozumi, Ibaraki, Osaka, Japan.; 7 Neurological Research Institute and Department of Molecular and Human Genetics, Baylor College of Medicine, Houston, Texas, USA.; 8 Telethon Institute of Genetics and Medicine (TIGEM), Pozzuoli, Italy.; 9 Medical Genetics, Department of Translational Medicine, Federico II University, Naples, Italy.; 10 Department of Internal Medicine and; 11 Institute for Reproductive and Developmental Sciences, University of Kansas Medical Center, Kansas City, Kansas, USA.

**Keywords:** Cell biology, Hepatology, Oncology, Autophagy, Liver cancer

## Abstract

Hepatocellular carcinoma (HCC) is heterogeneous, and hepatocyte plasticity is linked to poorer patient outcomes. A subset of human HCC harboring tuberous sclerosis complex 1 (*TSC1*) mutations exhibits more aggressive behavior. Transcription factor EB (TFEB) is a master regulator of lysosomal biogenesis and cell fate. We analyzed human normal and HCC tissue arrays for TFEB and CK19 expression, as well as bulk and single-cell RNA-seq datasets from mouse and human HCC, to define TFEB-associated transcriptional programs. We performed biochemical, histological, metabolomic, and transcriptomic analyses in liver-specific *Tsc1*-knockout (L-*Tsc1*–KO) and L-*Tsc1* *Tfeb*–double KO mice. Loss of hepatic *Tsc1* led to increased phosphorylation of S6 and 4EBP1, with paradoxical increases in TFEB nuclear translocation and activation. L-*Tsc1*–KO mice showed increased hepatocyte plasticity, decreased HNF4α, increased YAP activation, and spontaneous HCC with increased SOX9- and CK19-positive biliary epithelial cell–like cells at 8–12 months. Deletion of *Tfeb* dampened hepatic metabolic reprogramming and hepatocyte fate changes and inhibited tumor progression in L-*Tsc1*–KO mice. Increased TFEB activity was associated with increased YAP and SOX9 gene expression and high-grade malignant HCC in humans. These findings indicate that loss of hepatic TSC1 leads to noncanonical TFEB activation, promoting hepatocyte plasticity and tumor heterogeneity associated with high-grade malignancy in both mouse and human HCC.

## Abstract 2

Understanding how liver tumor heterogeneity is regulated by non-canonical mTORC1-TFEB will provide critical insights into tumor-specific therapies.

## Graphical abstract

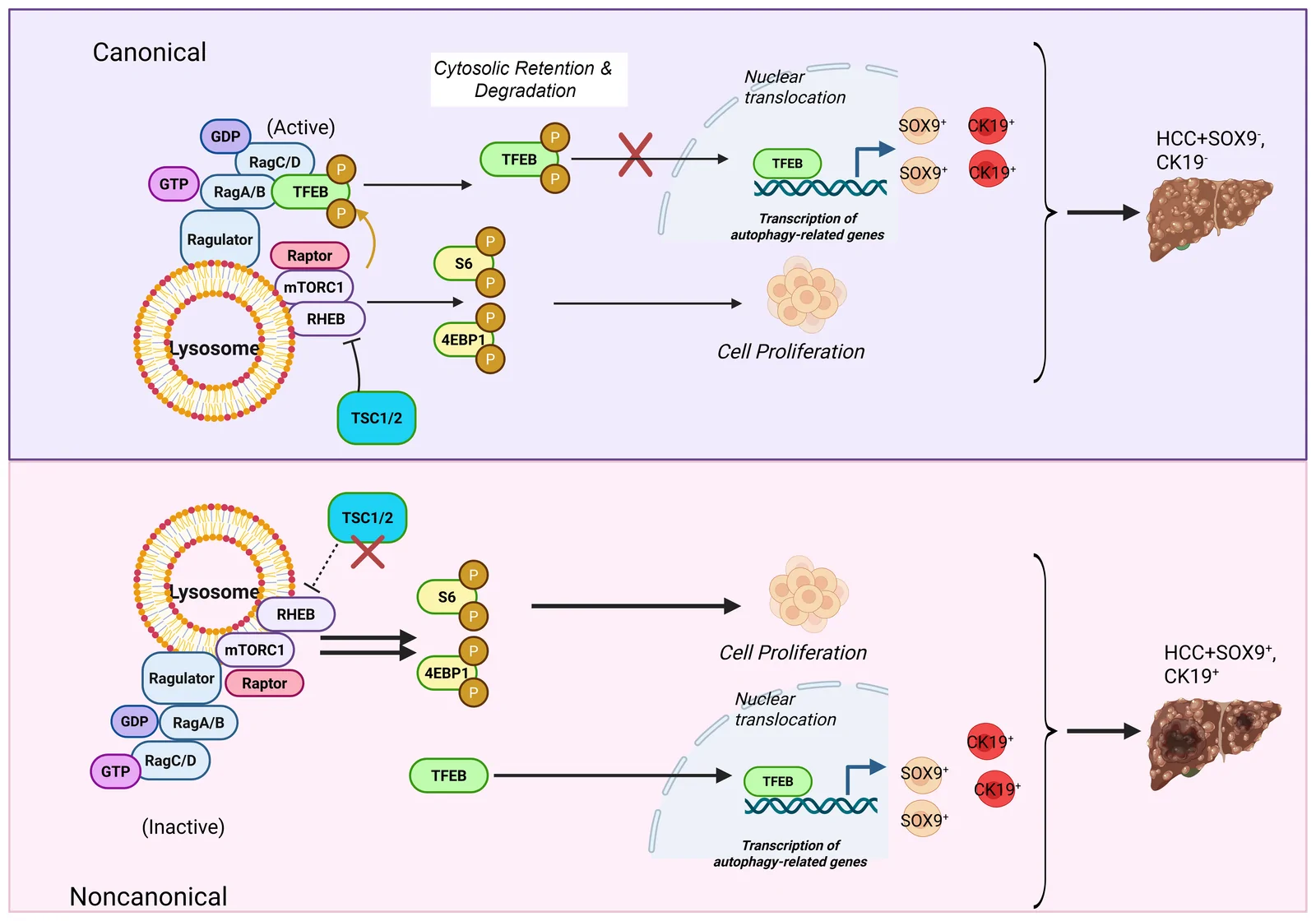

## Introduction

Liver cancer, mainly hepatocellular carcinoma (HCC) and intrahepatic cholangiocarcinoma, is the sixth most common cancer globally and a third leading cause of cancer death, with a predicted 1.3 million people expected to die from liver cancer in 2040 (1, 2). Risk factors for HCC include aging, chronic viral hepatitis, and several environmental factors such as tobacco use, aflatoxin exposure, chronic alcohol consumption, obesity, and metabolic dysfunction–associated steatotic liver disease (3). HCC is an extraordinarily heterogeneous malignant disease characterized by high hepatocyte plasticity and greater transcriptomic diversity, both of which are associated with worse patient prognosis (4, 5). Current therapeutic approaches yield only modest improvements in survival among patients with HCC, largely because our understanding of the molecular mechanisms that regulate hepatocyte fate changes and plasticity remains incomplete.

Mechanistic target of rapamycin (mTOR) is a highly conserved serine-threonine protein kinase that forms 2 structurally and functionally distinct multiprotein complexes, namely mTOR complex 1 (mTORC1) and mTORC2, which integrate growth factors and nutrient cues to regulate cell growth and metabolism (6). The tuberous sclerosis complex (TSC) is a heterodimer comprising TSC1 and TSC2 and functions as a GTPase-activating protein (GAP) for the small Ras-related GTPase Rheb (Ras homolog enriched in brain), thereby negatively regulating mTORC1 (6, 7). The activation of mTORC1 is also regulated by 4 Rag GTPases: RagA, RagB, RagC, and RagD. Specifically, GDP-bound RagC/D proteins recruit mTORC1 to lysosomes for mTORC1 activation (8, 9). Mutations in either TSC1 or TSC2 are associated with a subset of human HCC with high malignancy (10, 11). Liver-specific deletion of *Tsc1* leads to persistent mTORC1 activation and spontaneous HCC development in aged mice (12, 13). However, the downstream events of mTORC1 activation contributing to HCC remain largely unknown. Transcription factor EB (TFEB) is a master transcription factor that regulates autophagy-related and lysosomal biogenesis genes and belongs to the coordinated lysosomal expression and regulation (CLEAR) gene network (14, 15). Genetic alterations in TFEB are associated with various types of human cancers, including renal carcinomas, non–small cell lung cancer, pancreatic ductal adenocarcinoma, and breast cancer. mTORC1 phosphorylates TFEB at the lysosomal surface, leading to its cytosolic retention and degradation (16). Furthermore, TFEB is able to promote mTORC1 activity through a previously described feedback loop (17). Surprisingly, recent evidence supports a noncanonical TFEB activation pathway that is independent of traditional mTORC1 activity (18). Folliculin (FLCN) is a GAP for RagC and RagD GTPases. Active RagC and RagD enable mTORC1-mediated phosphorylation and cytoplasmic retention of TFEB and transcription factor E3 (TFE3). Both belong to the microphthalmia-associated transcription factor (MiT/TFE) family. Loss of TSC2 in the mouse kidney or FLCN in the mouse liver results in increased phosphorylation of canonical mTORC1 substrates (S6 and eukaryotic translation initiation factor 4E-binding protein 1, 4EBP1) but decreased phosphorylation of TFEB and TFE3, thereby activating TFEB and TFE3 (19, 20). Activated TFEB and TFE3 promote tissue injury and tumorigenesis in the kidney (18) and liver (19, 20) that lack either *Tsc2* or *Flcn*.

In the present study, we aimed to investigate the role and mechanisms of TFEB in the liver pathogenesis and HCC in liver-specific *Tsc1*-knockout (L-*Tsc1*–KO) mice and human HCC samples.

## Results

### Loss of hepatic TSC1 activates TFEB and increases ductular–fibrotic remodeling in the mouse livers despite mTORC1 activation.

At 2 months of age, L-*Tsc1*–KO mouse livers showed markedly increased levels of phosphorylated (p-) S6 and p-4EBP1, indicating increased mTORC1 activity (Figure 1A). Unexpectedly, despite the well-established role of mTOR in suppressing TFEB activity, Western blot analysis revealed increased hepatic levels of TFEB and TFE3, along with the protein levels of their target genes (FLCN, RagA, RagC, and many lysosomal proteins VATP6V1a, VATP6V1b2, LAMP1, LAMP2, and NPC1), in L-*Tsc1*–KO mouse livers (Figure 1A). The hepatic levels of several TFEB-mediated lysosomal proteins were moderately increased as early as day 7 in L-*Tsc1*–KO mice, even though hepatic TFEB levels decreased (Supplemental Figure 1; supplemental material available online with this article; https://doi.org/10.1172/JCI206334DS1). H&E staining showed increased ductular structures, and IHC staining showed increased TFEB and TFE3 staining in a subset of cells that are CK19 positive (Figure 1B and Supplemental Figure 2). Moreover, RNA-seq analysis showed increased expression of TFEB target genes (Figure 1C). Gene Ontology (GO) and Kyoto Encyclopedia of Genes and Genomes (KEGG) analyses revealed enrichment for small GTPase–mediated signaling pathways (Figure 1D) and the lysosomal pathway (Figure 1E) in the livers of L-*Tsc1*–KO mice. Together, these data indicate that loss of TSC1 activates TFEB-mediated lysosomal pathways despite increased mTORC1 activity in mouse livers.

IHC staining revealed an increased number of CK19-, SOX9-, and YAP-positive cells and ductular structures in 2-month (2M) L-*Tsc1*–KO mouse livers (Figure 2A). Consistent with the IHC data, Western blot analysis revealed markedly increased hepatic levels of CK19, SOX9, YAP, and α–smooth actin (α-SMA) in L-*Tsc1*–KO mouse livers (Figure 2B). RNA-seq analysis revealed a shift in liver cell identity, with gene signatures associated with hepatocytes markedly decreased. In contrast, signatures of progenitor cells/cholangiocytes, hepatic stellate cells (HSCs), and Kupffer cells (KCs)/macrophages were enriched in L-*Tsc1*–KO livers (Figure 2C). Together, these data indicate that loss of hepatic *Tsc1* induces accumulation of progenitor cells, ductular reactions (DRs), and fibrosis associated with TFEB activation, despite mTORC1 activation.

### TFEB overexpression in hepatocytes is sufficient to induce hepatocyte transdifferentiation to hepatic progenitor/biliary epithelial cells and cystogenesis in the mouse liver.

We next determined whether TFEB overexpression alone in hepatocytes would also change hepatocyte identity toward progenitor/biliary phenotypes by generating hepatocyte-specific TFEB-knockin (TFEB-KI) mice (Supplemental Figure 3A). To generate a gene dose-response for TFEB expression in the mouse liver, *Tfeb*-3xFlag^fl/fl^ (Rosa26-LSL-Tfeb-3xFlag) mice were administered different doses of AAV8-TBG-Cre. *Tfeb*-3xFlag^fl/fl^ mice receiving a high dose of AAV-TBG-Cre [TFEB-KI (H)] showed marked hepatomegaly, along with elevated serum alanine aminotransferase (ALT), alkaline phosphatase, and bile acids, without altering serum triglyceride or cholesterol. *Tfeb*-3xFlag^fl/fl^ mice receiving a low dose of AAV-TBG-Cre [TFEB-KI (L)] showed similar but more moderate phenotypes compared with TFEB-KI (H) mice (Supplemental Figure 3, B–D). RNA-seq analysis further showed decreased expression of hepatocyte gene signatures but increased expression in cholangiocyte/progenitor cells, HSCs, and KCs/macrophages (Supplemental Figure 3E). Western blot analysis showed that TFEB-KI mice had dose-dependent increased levels of proteins of TFEB target genes, including LAMP1, LAMP2, PGC1α, VATP6V1a, and VATP6V1b2, as well as autophagy markers LC3-II and p62. Similar to L-*Tsc1*–KO mice, TFEB-KI mice increased HNF4α-P2 (fetal form) and progenitor/biliary cell markers (EPCAM, CK19, SOX9, YAP, and TAZ) but decreased HNF4α-P1 (adult form), cytochrome P450 2E1 (CYP2E1), and glutathione synthetase (Supplemental Figure 3F), indicating that TFEB overexpression induced cell type identity reprogramming by decreasing mature hepatocytes while increasing the progenitor cell population in mouse livers. Similar to L-*Tsc1*–KO mice, Western blot analysis and RNA-seq analysis also showed increased YAP-TAZ and SOX9 expression in TFEB-KI mice (Figure 2, D and E). Moreover, H&E staining revealed that TFEB-KI mice had an increased number of hepatocytes with swollen, pale cytoplasm, a key feature of hepatocyte degeneration. IHC staining also revealed decreased HNF4α-P1– and glutamine synthetase–positive cells but increased YAP-positive cells in TFEB-KI mice (Supplemental Figure 3G).

To further investigate the effects of TFEB overexpression on hepatocyte cell fate, we bred *Tfeb*-3xFlag^fl/fl^ mice with mT/mG (ROSAmT/mG) reporter mice. Before Cre recombination, cells exhibited widespread cell membrane–localized tdTomato (mT) fluorescence. After AAV8-TBG-Cre injection and recombination, hepatocytes expressing Cre recombinase showed cell membrane–localized EGFP (mG) fluorescence, replacing the red fluorescence (Figure 2F). Lineage-tracing results showed that Cre-positive cells (green, TFEB overexpression) had almost no HNF4α staining but increased SOX9 staining, whereas Cre-negative cells (red, no TFEB overexpression) were HNF4α positive but SOX9 negative (Figure 2G), supporting the notion that TFEB drives mature hepatocytes to dedifferentiate into progenitor/ductular cells.

Increased ductular, CK19-positive, and SOX9-positive structures and fibrosis, along with decreased hepatocyte signature gene expression and increased biliary epithelial cells (BECs)/progenitor cells, HSCs, and KCs/macrophages, persisted in 6-month-old L-*Tsc1*–KO mice (Supplemental Figure 4, A and B). At 12 months, small, cyst-like, CK19-positive structures developed in L-*Tsc1*–KO mice (Supplemental Figure 4C). At 8–10 months, most TFEB-KI mice met humane endpoints and had to be euthanized. These mice developed large cysts concurrent with increased hepatomegaly and moderately elevated serum ALT levels (Supplemental Figure 5, A and B). Retrograde injection of ink into the biliary tree in 6-month-old mice revealed defective bile duct structures in TFEB-KI mice (Supplemental Figure 5C). H&E staining showed increased immune cells surrounding the cysts, and IHC staining revealed that the cyst-lining cells are YAP, SOX9, and CK19 positive, with fibrosis revealed by Sirius Red staining. Intriguingly, these cyst-lining cells also had much higher TFEB staining (Supplemental Figure 5D). RNA-seq analysis showed downregulation of bile acid metabolism but upregulation of cystic disease–related genes (*Pkhd1*, *Prkcsh*, *Lrp5*) as early as 2 weeks after TFEB overexpression in the mouse livers (Supplemental Figure 5E). Together, these data indicate that TFEB activation suppresses hepatocyte identity and increases progenitor/BECs (or ductular cells), resulting in cystic biliary hyperplasia in mouse livers.

### TFEB deletion partially reverses ductular–fibrotic remodeling in TSC1-deficient livers.

We next generated L-*Tsc1* *Tfeb*–DKO (double-knockout) mice to determine the role of TFEB in the pathogenesis of *Tsc1*-KO mice. The liver weight/body weight ratio and serum ALT levels did not differ significantly between the L-*Tsc1*–KO and DKO 2M and 6M mice (Figure 3A and Supplemental Figure 6A). H&E and IHC staining, as well as Sirius Red staining, showed that increased DR- and CK19-positive cells, as well as Sirius red–positive staining areas in 2M and 6M L-*Tsc1*–KO mice, were markedly attenuated in the same age of DKO mice (Figure 3, B and C, and Supplemental Figure 6B). Consistent with the histology data, Western blot analysis showed that increased CK19 and α-SMA in 2M and 6M L-*Tsc1*–KO mice were markedly attenuated in the same age of DKO mice (Figure 3D and Supplemental Figure 6C). Principal component analysis (PCA) of RNA-seq analysis of hepatic tissues clearly showed gene expression separation of WT, L-*Tsc1*–KO, and DKO mice, with DKO mice tending to shift toward WT mice (Supplemental Figure 7A). Heatmap analysis of the RNA-seq data showed that loss of hepatic TFEB partially reversed the increased expression of TFEB target genes and the changes in the liver cell identity gene expression signature in L-*Tsc1*–KO mouse livers (Figure 3E and Supplemental Figure 7, B and C). Furthermore, loss of hepatic TFEB decreased the levels of p-YAP, total YAP, TAZ, and HNF4α-P1 without affecting HNF4α-P2 and total HNF4α in L-*Tsc1*–KO mice (Supplemental Figure 7D). Increased levels of p-4EBP1 but not p-S6 were also attenuated in DKO mice (Supplemental Figure 7E). Immunoprecipitation assay showed that TFEB-3xFlag interacts with endogenous YAP and SOX9 in TFEB-KI mouse livers (Supplemental Figure 8A). Heatmap analysis of the RNA-seq data showed that loss of hepatic TSC1 increased expression of modules for several growth factors, including *Vegfb*, *Pdgfb*, and *Tgfb*, which may favor HSC activation and fibrosis. However, expression of these fibrogenic growth factors was blunted in *Tsc1* *Tfeb*–DKO mice (Supplemental Figure 8B). Knockdown of *Yap/Ta*z using a GalNAc-siRNA improved DR and fibrosis, although it had no effect on serum ALT levels in L-*Tsc1*–KO mice (Supplemental Figure 9, A–C). These data indicate that TFEB activation promotes YAP/TAZ–mediated ductular and fibrogenic changes, whereas loss of TFEB attenuates these changes in L-*Tsc1*–KO mice.

### Increased tumor burden and SOX9-positive HCC with increased CK19-positive ductular cell infiltration in L-Tsc1–deficient mice is driven by TFEB activation.

L-*Tsc1*–KO mice developed spontaneous tumors with 100% incidence in both male and female mice at 12 months of age, and deletion of hepatic TFEB did not affect the tumor incidence significantly in L-*Tsc1*–KO mice, with only 2 out of 18 DKO mice not developing tumors (Figure 4, A and B). The liver weight/body weight ratio and serum ALT levels decreased almost 50% in DKO mice compared with L-*Tsc1*–KO mice but did not reach statistical differences (Figure 4C). However, the overall tumor size was significantly decreased in DKO mice, with >60% of L-*Tsc1*–KO mice harboring tumors >20 mm, whereas >80% of DKO mice had smaller tumors (Figure 4D). Together, these data indicate that TFEB activation promotes tumor growth and overall tumor burden in the *Tsc1*-deficient conditions.

We further characterized tumor pathology in L-*Tsc1*–KO and DKO mice. We found that all the L-*Tsc1*–KO mice developed multiple HCC tumors, characterized by a typical high nuclear/cytoplasmic ratio in tumor cells with pseudogland-like structures within preexisting liver cell plates (Figure 4E and Supplemental Figure 10, A and B, black arrow). Notably, these HCC tumors are enriched with small, uniform, cuboidal cells that form tubular structures, with increased CK19^+^ ductular cells (Figure 4E and Supplemental Figure 10A). Across 10–12 months, 14/14 exhibited HCC mixed with CK19^+^ cells in L-*Tsc1*–KO mice whereas 8/10 DKO mice exhibited HCC, with only 2/10 DKO mice having HCC mixed with CK19^+^ cells (Figure 4, E and F, and Supplemental Figure 10, A and B). Relative to adjacent nontumor (“N”), increased YAP, glypican-3, and Ki67, as well as PCNA-positive cells, were observed in the tumor areas (T), consistent with malignant transformation in L-*Tsc1*–KO mice. Interestingly, increased nuclear TFEB staining and elevated lysosomal markers, including LAMP1 and NPC1, as well as TFEB target gene protein p62, were found in L-*Tsc1*–KO mouse tumors that are predominantly HCC mixed with CK19^+^ cells (Figure 5, A and B; Figure 6A; and Supplemental Figure 11, A and B). HCC cells are HepPar-1 positive, and a subset of these cells also showed increased nuclear SOX9 and TFEB staining, which were not observed in DKO mice (Figure 6B, arrow, and Figure 6C). Increased CK19-positive cells are also SOX9 and TFEB positive but are HePar-1 negative (Figure 6, B and C, and Supplemental Figure 11B), suggesting these CK19-SOX9-TFEB triple-positive cells are ductular/progenitor cells mixed with the HCC cells. Importantly, increased lysosomal markers and TFEB target proteins (LAMP1, NPC1, and p62) as well as CK19-positive ductular cells decreased in the tumors of DKO mice based on IHC and fluorescence staining, as well as Western blot analysis. NRF2 activation has been linked to liver tumorigenesis, and the levels of KEAP1, NQO1, GCLC, and GCLM increased in tumors, with no difference between L-*Tsc1*–KO and DKO mice (Supplemental Figure 12A). Moreover, levels of p-AKT, p-ERK, p-GSK3β, and p-STAT3 increased in tumors to similar levels in both L-*Tsc1*–KO and DKO mice, whereas DNA damage-related markers such as p21, p53, and γ-H2AX, as well as proliferation markers such as PCNA and Ki67, along with cleaved caspase-3, were higher in L-*Tsc1*–KO tumors than in DKO tumors (Supplemental Figure 12B). The malignant liver cancer marker glypican-3 increased in L-*Tsc1* tumors but not in DKO tumors (Figure 5B and Supplemental Figure 12B). Collectively, these results indicate that TFEB activation increased SOX9-positive HCC and created a microenvironment enriched with CK19-positive ductular cells, both associated with HCC growth and malignancy.

### Overexpression of a constitutively active RagC promotes the relocation of hepatic TFEB into the cytoplasm and attenuates DR in L-Tsc1–deficient mouse livers.

GDP-bound RagC (constitutively active, but not GTP-bound RagC) directly recruits TFEB to lysosomal mTORC1 for its phosphorylation and inactivation (21). We next investigated whether constitutively active RagC (RagC^CA^) would affect TFEB lysosomal recruitment and contribute to DR in L-*Tsc1*–KO mice. Overexpression of RagC^CA^ markedly decreased DR as revealed by the H&E and IHC staining for CK19 without affecting liver weight/body weight ratio and serum ALT levels in L-*Tsc1*–KO mice (Supplemental Figure 13, A–D). More importantly, overexpression of RagC^CA^ relocated the nuclear TFEB into the cytoplasm (Supplemental Figure 13D, arrows). Western blot analysis also showed that overexpression of RagC^CA^ decreased hepatic CK19 and α-SMA and increased HNF4α-P1 as well as the levels of p-S6 and p-4EBP1 but had little effect on the levels of TFEB and 2 lysosomal markers (VATP6V1a and VATP6V1b2) (Supplemental Figure 13E). Overexpression of RagC^CA^ shifted TFEB from nucleus to cytosol and increased TFEB localization to LAMP1-positive lysosomes (Supplemental Figure 13, F and G). Together, these data indicate that overexpression of RagC^CA^ partially inhibits TFEB activation, thereby attenuating TFEB-dependent DR in L-*Tsc1*–KO mice.

### TFEB deletion partially restores metabolic homeostasis in TSC1-deficient livers.

PCA of untargeted metabolomics showed clear separation of overall metabolites among WT, TFEB-KI, L-*Tsc1*–KO, and DKO mice, except that TFEB-KO mice are very similar to the WT mice (Supplemental Figure 14A). Interestingly, the overall metabolite profiles of DKO mice tended to shift toward WT and TFEB-KO mice. Further analysis of these metabolic pathways showed that increased fatty acid and amino acid metabolism in L-*Tsc1*–KO mice was largely corrected by the loss of hepatic TFEB (Supplemental Figure 14, B and C). A global heatmap analysis likewise revealed widespread reversion of metabolite levels toward WT levels in DKO mice compared with L-*Tsc1*–KO mice. TFEB overexpression showed a pattern of changes similar to, but stronger than, the metabolite profiles in the L-*Tsc1*–KO mice (Figure 7A). Increased tryptophan, γ-glutamyl amino acid, ceramide, and nucleotide metabolism is often associated with increased liver cancer cell growth and poor prognosis, and metabolism of all was increased in L-*Tsc1*–KO mice and further enhanced in TFEB-KI mice but generally diminished in the DKO mice (Figure 7, B–D, and Supplemental Figure 15, A–C). Transcriptomic analysis showed decreased expression of kynurenine core genes (Figure 7C) and altered nucleotide metabolism (Figure 7D) and purine pathway gene expression (Figure 7E and Supplemental Figure 15D) in L-*Tsc1*–KO mice, which were generally corrected in DKO mice. Polyamine-related metabolites such as putrescine, spermidine, and N-acetyl-isoputreanine that can promote cell proliferation and growth were markedly elevated in TFEB-KI and moderately elevated in L-*Tsc1*–KO mice (Supplemental Figure 16A). Transcriptomics analysis showed decreased expression of breakdown and utilization but increased gene expression of transport of polyamine (Supplemental Figure 16B). L-*Tsc1*–KO and TFEB-KI mice had increased, but DKO mice had decreased, lysosomal bis(monoacylglycero)phosphate (BMP) lipids (Supplemental Figure 17A), which is consistent with a recent report that hepatic TFEB activation increases BMP (22). Altered BMP metabolism gene expression in L-*Tsc1*–KO mice was also corrected in DKO mice (Supplemental Figure 17B). L-*Tsc1*–KO and TFEB-KI mice had increases in several TCA cycle metabolites, which are slightly corrected in DKO mice (Supplemental Figure 18A). In contrast, there were marked gene expression changes in the electron transport chain and TCA core genes in L-*Tsc1*–KO mice that were markedly reversed in DKO mice (Supplemental Figure 18B). L-*Tsc1*–KO mice also had increased levels of several bile acids, including deoxycholate, taurodeoxycholate, and taurolithocholate, which were attenuated in DKO mice (Supplemental Figure 19). Notably, increased tryptophan, γ-glutamyl amino acid, ceramide, nucleotide, and polyamine metabolism and several TCA cycle metabolites in L-*Tsc1*–KO and TFEB-KI mice were generally correlated at the transcriptomic level of the key genes for these metabolic pathways, suggesting that TFEB activation drives metabolic rewiring and that TFEB can directly or indirectly regulate the expression of these metabolic genes. Together, these data indicate that hepatic TFEB acts as a metabolic amplifier, rewiring central carbon, lipid, and nucleotide pathways that favor tumor cell growth in the TSC1-deficient liver with hyperactive mTORC1. Removing TFEB dampens this metabolic reprogramming and partially restores homeostasis, supporting the notion that lysosomal signaling feeds back onto cytosolic metabolism and proliferative readiness for tumor cell growth.

### Increased hepatic TFEB is correlated with higher grade malignant human HCC and associated with expression of biliary/progenitor genes.

Bioinformatics analysis using The Cancer Genome Atlas (TCGA), the LCI database, and the Mongolia database indicated considerable heterogeneity in TFEB-CLEAR network activity in both normal human liver and HCC tissues, with TFEB-CLEAR network activity significantly higher in tumors than in normal tissues (Figure 8A). Moreover, the TFEB-CLEAR network activity increased with advancing clinical stage, suggesting a positive association with HCC aggressiveness (Figure 8B). We next examined TFEB protein expression in clinical samples from 2 tissue microarrays (TMAs). One TMA (LV2089) contains 16 normal human liver samples along with 3 stage I, 63 stage II, and 112 stage III HCC samples and 12 bile duct adenoma (Supplemental Figure 20). Another TMA (LV2089a) contains 82 stage I–II and 38 stage III–IV HCC and 9 HCC and cholangiocarcinoma mixed tumors (Supplemental Figure 21). Similar to the TFEB-CLEAR activity, IHC staining for TFEB revealed significant heterogeneity in its expression between normal liver and HCC tissues, with no substantial differences observed between the normal and HCC stage I and II, but a decrease in HCC stage III (Supplemental Figure 20, A and B). The levels of p-S6 can be used to correlate with mTORC1 activity, and our previous IHC staining for p-S6 also revealed large heterogeneity of p-S6 staining among HCCs (13). We found that more than 58% of stage III HCC samples, whereas only 33.3% of stage I HCC, had high p-S6 and high TFEB staining. In contrast, more than 66.6% of stage I HCC, whereas only 7.8% of stage III HCC, had high p-S6 and low TFEB (Supplemental Figure 20C). There were no significant differences in TFEB staining between stage I–II and stage III–IV HCC and cholangiocarcinoma (CCA), but the TFEB staining was significantly increased in mixed tumors (Supplemental Figure 21, A and B, and Supplemental Figure 22, A and B). High-magnification imaging analysis revealed more cytosolic TFEB staining in stage I, mixed cytosolic and nuclear TFEB staining in stage II, and increased nuclear TFEB staining in stage III HCC (Supplemental Figure 21C). However, both nuclear and cytosolic TFEB staining increased in CCA regardless of the stage (Supplemental Figure 22C). This increased nuclear TFEB staining in more aggressive-stage HCC is consistent with the TFEB-CLEAR network activity data (Figure 8, A and B). Moreover, TCGA survival analysis revealed that patients with high TFEB expression exhibited poorer OS than those with low TFEB (log-rank *P* < 0.05), and a TFEB-CLEAR network target–based module score stratified survival in a similar manner (log-rank *P* < 0.05) (Figure 8, C and D). We recently published a spatial, multiregional, single-cell transcriptomic profiling of HCC (GSE189903) (23), in which cells were isolated from 3 tumor cores (T1, T2, and T3), a tumor border (B), and adjacent normal tissue (N). TFEB-CLEAR network genes were most prominent in tumor regions, with comparatively lower activity in adjacent normal tissue, and this enrichment remained evident after collapsing T1, T2, and T3 into a single merged tumor group (Figure 8, E–G).

Because TFEB induces hepatocyte plasticity and differentiation in mice, we next asked whether TFEB transcriptional activity also affects hepatocyte lineage plasticity in human liver cancers. Using the combined TCGA-LIHC (liver hepatocellular carcinoma) and TCGA-CHOL (cholangiocarcinoma) expression matrix, we found that tumors with high TFEB activity showed coordinated upregulation of lysosomal and endolysosomal genes, together with enrichment of a YAP and SOX9 progenitor-like program, whereas expression of hepatocyte markers such as CYP7A1, HNF4A, ALB, and APOA1 was markedly decreased (Figure 9A). Consistent with this pattern, TFEB-CLEAR activity scores were positively correlated with biliary/progenitor genes including *SOX9*, *EPCAM*, and *KRT19* while showing an inverse relationship with hepatocyte lineage factors (Figure 9A). Accordingly, *SOX9*, *EPCAM*, and *KRT19* expressions were significantly higher in TFEB-high tumors than in TFEB-low tumors (Figure 9B). Further TMA staining for HepPar-1 and CK19 showed decreased HepPar-1 and increased CK19 staining in high-stage HCC compared with stage I HCC, but no significant differences in HepPar-1 or CK19 were observed among stage II–IV HCC (Figure 9, C–E). These data suggest that HCC is associated with greater loss of hepatocyte identity and an increase in CK19-positive cells, but the roles of these changes in HCC progression remain unclear and warrant further investigation. Together, these analyses indicate that elevated TFEB-CLEAR network activity in liver cancers aligns with a transcriptional state marked by increased lysosome biogenesis and progenitor-like features, consistent with enhanced hepatocyte plasticity and higher grade malignancy observed in mice.

## Discussion

Similar to kidney-specific TSC2-KO mice, we found that loss of hepatic TSC1 led to increased phosphorylation of S6 and 4EBP1, as well as increased TFEB nuclear translocation and activation in mouse livers, indicating that the uncoupling of mTORC1 and TFEB in the absence of TSC1/2 is conserved across tissues. These paradoxical findings in mTORC1-mediated TFEB regulation likely arise from mTORC1’s preferential access to and phosphorylation of distinct substrates (e.g., S6 vs. TFEB) under specific conditions. In the absence of TSC1/2, the hyperactivated mTORC1 can access and phosphorylate S6 but not TFEB. We found that overexpression of RagC^CA^ (GDP-bound) was partially able to relocalize TFEB to the cytoplasm in TSC1-deficient mouse hepatocytes, likely through increased TFEB phosphorylation at the mTORC1 sites. Similarly, overexpression of FLCN, a GAP for RagC, has been reported to increase TFEB phosphorylation and promote TFEB relocalization to the cytoplasm in TSC2-deficient cells (21). These findings support the notion that the activation status (or nucleotide state) of RagC is critical for mTORC1’s access to TFEB in the absence of TSC1/2 (Supplemental Figure 19).

Lysosomes are the final stage of autophagy and are critical to its completion by fusing with autophagosomes to degrade autophagic cargo and by serving as essential cellular quality control and survival mechanisms in response to various stress conditions (24). Hence, increasing autophagy activity by improving TFEB-mediated lysosomal biogenesis has long been considered beneficial for maintaining cellular balance and protecting against various harmful factors and diseases (15, 25, 26). These include aging, neurodegenerative diseases, as well as various liver diseases such as drug-induced liver injury, early-stage alcohol-associated liver disease (steatosis and hepatocyte death), and metabolic dysfunction–associated steatotic liver disease. For instance, overexpression of HLH-30 (the mammalian homolog of TFEB) extends life span in *C*. *elegans* (27). Moderate overexpression of TFEB using an adenovirus-TFEB protected against high-fat diet– and alcohol-induced steatosis as well as acetaminophen-induced liver injury in mice (28–31). In addition to these beneficial effects of TFEB (the bright side), constitutive activation of TFEB induces cell fate changes. It promotes cell proliferation in several types of cancer (the dark side) (18, 21, 32–34). Earlier studies show that chromosomal translocations lead to the overexpression of either TFEB or the TFEB paralog TFE3, causing renal cell carcinoma (35). Furthermore, loss-of-function mutations of the RagC/D GAP FLCN and TSC2 lead to constitutive activation of TFEB in kidney-specific mouse models of Birt-Hogg-Dube syndrome, respectively. Genetic deletion of TFEB rescues renal cystogenesis and kidney cancer in these mouse models (20). In the present study, we found that overexpression of TFEB in hepatocytes directly induces hepatocyte transdifferentiation into BEC-like cells. Liver-specific TFEB-transgenic mice develop increased liver cyst formation and cholangiocarcinoma-like phenotypes at around 8 months of age, with an inferior health status that necessitates euthanasia. Removing TFEB dampens this metabolic reprogramming, partially restores homeostasis, and inhibits tumor progression in L-*Tsc1*–KO mice, consistent with a model in which lysosomal signaling feeds back onto cytosolic metabolism, proliferative readiness, and cell plasticity. Current evidence strongly supports the notion that TFEB plays a dual role in regulating cellular homeostasis, cell fate (de)differentiation, and cancer. In the liver, short-term, mild overexpression or activation of TFEB is likely beneficial, as it increases lysosome biogenesis and autophagy. In contrast, chronic, higher levels of TFEB activation will primarily affect liver cell fate and induce cell type repopulation, resulting in liver failure or liver cancer.

HCC is a highly heterogeneous tumor with high cellular diversity, a key factor in therapeutic failures. TFEB directly promotes a shift in hepatocyte identity toward YAP/TAZ–, SOX9-, and CK19-positive BEC-like cells, which may contribute to tumor growth and high malignancy grade, as TFEB deletion inhibited cell proliferation and reduced tumor size in mice. Furthermore, TFEB-CLEAR network signature genes are enriched with YAP/TAZ and SOX9 signature genes in HCC, which also have high cell plasticity, heterogeneity, and malignancy with a poor free survival rate (4, 36). CK19- and SOX9-positive BEC-like cells are known to secrete various cytokines and chemokines that recruit immune cells to the tumor, alter the tumor microenvironment and its diversity, and promote tumor cell growth and enhanced malignancy. These increased cell plasticity and diversity in HCC may account for the overall low survival observed in HCC with high TFEB expression and TFEB-CLEAR activity. Future work is needed to identify and manipulate the possible TFEB-mediated secreted cytokines and chemokines from BEC-like cells in HCC pathogenesis. Moreover, TFEB activation broadly influences metabolic rewiring in *Tsc1*-deficient livers, including lysosomal lipid metabolism, amino acid and tryptophan metabolism, nucleotide metabolism, and polyamine pathways. These metabolic changes may also contribute to HCC malignancy, although our study does not identify a single metabolic pathway as the sole driver of hepatocyte fate change.

In summary, we delineate a subtype of TSC1-loss HCC characterized by noncanonical RagC-dependent TFEB activation and high TFEB-CLEAR network activity, promoting cell plasticity and heterogeneity and associated with poor outcomes. Identifying this subset of patients with HCC for precision medicine by inhibiting TFEB and its homologs (such as TFE3) may yield beneficial treatments.

## Methods

### Sex as a biological variable.

Males have a higher incidence of HCC than females. Our study examined both male and female animals, and results are reported for both sexes.

### Animal experiments.

*Tfeb*^fl/fl^ (C57BL/6J) mice were generated as described previously (28, 29). *Tsc1*^fl/fl^ (C57BL/6J) and albumin-Cre (Alb-Cre; C57BL/6J, stock 003574) mice were obtained from The Jackson Laboratory. We crossed *Tfeb*^fl/fl^ and *Tsc1*^fl/fl^ lines with Alb-Cre to obtain liver-specific knockout cohorts, L-*Tsc1*–KO, L-*Tfeb*–KO, and L-*Tsc1* *Tfeb*–DKO mice. Mice were maintained under specific pathogen–free conditions in a barrier facility, and all procedures were approved by the University of Kansas Medical Center IACUC (protocol 24-04-387). Animals were euthanized at 2, 6, or 12 months of age (2M, 6M, 12M). For the GalNAc-siRNA treatment, 2-month-old L-*Tsc1*–KO mice were injected with GalNAc-siRNA control and GalNAc-siRNA *Yap/Taz* (10 mg/kg, subcutaneous) for 10 days. All animals were fasted for 6 hours before tissue collection.

### RNA extraction and library preparation.

Liver tissue from 2-month-old male WT, L-*Tsc1*–KO, and L-*Tsc1 Tfeb*–DKO mice (following a 6-hour fast) was homogenized in TRIzol (Invitrogen). RNA quantity and integrity were evaluated by Qubit assays and Agilent TapeStation, respectively. For library construction, 1 μg total RNA per sample was processed with the NEBNext Ultra II Directional RNA Library Prep Kit for Illumina (New England Biolabs). The workflow included oligo-dT bead capture of poly(A)^+^ RNA, controlled fragmentation, strand-specific cDNA synthesis, end repair/A-tailing, adaptor ligation, and limited-cycle PCR. Libraries passed quality control (Qubit/TapeStation), then were pooled on an equal-mass basis, and the pool concentration was verified by KAPA Library Quantification qPCR (Roche). Indexed libraries were sequenced in multiplex on an Illumina NextSeq 550 to generate 75 bp single-end reads. Primary base calling used RTA, and bcl2fastq2 was used for demultiplexing to FASTQ.

### RNA-seq data processing.

Reads were aligned to *Mus musculus* reference GRCm38 (Ensembl r90) using HISAT2 v2.1.0.13. Gene-level counts were obtained with HTSeq-count v0.6.0. Differential expression was computed in DESeq2 (R), with Benjamini-Hochberg adjustment; transcripts with FDR (*q*) < 0.05 were considered significant. Downstream visualization and enrichment (heatmaps, PCA, GO/KEGG) were performed in R as detailed in *Bioinformatics statistical analysis and software*.

### Liver metabolomics.

Untargeted metabolomics was performed by Metabolon as described previously (13), yielding 907 annotated small molecules. Data were log-transformed; when needed, missing values were imputed to the per-metabolite minimum. Group differences were assessed with Welch’s *t* test. The number of metabolites meeting *P* ≤ 0.05 (and 0.05 < *P* < 0.10) is reported, and pathway-level summaries are shown in the figures.

### Liver injury assays and tissue lysates.

Hepatic injury was assessed by serum ALT measurement and H&E histology using standard procedures. Whole-liver lysates were prepared in RIPA (1% NP-40, 0.5% sodium deoxycholate, 0.1% SDS) supplemented with protease inhibitors.

### IHC and Sirius Red.

Paraffin sections were deparaffinized and rehydrated, endogenous peroxidase was quenched (Sigma), and sections were incubated with primary antibodies (typical dilution 1:200) followed by HRP detection (ImmPACT NovaRED, Vector SK-4805, or DAB, Vector SK-4100) and hematoxylin counterstain. Antibodies included F4/80, CK19, TFEB, KEAP1, glutamine synthetase, YAP, HNF4A, SOX9, glypican-3, and p62 (full details in Supplemental Table 1). For fibrosis, sections were stained with Sirius Red, and the positive area was quantified in ImageJ (NIH).

### Immunofluorescence staining.

Paraffin sections were deparaffinized and rehydrated, followed by heat-induced (98°C) epitope retrieval in citrate buffer (pH 6.0) for 30 minutes. Slides were allowed to cool (~2 hours at room temperature), then blocked in 5% BSA and incubated overnight at 4°C with primary antibodies (typically 1:100). After PBS washes, species-appropriate secondary antibodies (Jackson ImmunoResearch; typically, 1:500) were applied for 1 hour at room temperature. To suppress background signal, sections were treated with Vector TrueVIEW Autofluorescence Quenching Reagent (SP-8400-15) according to the manufacturer’s instructions, rinsed, and coverslipped. Images were acquired on a CSU-W1 spinning-disk confocal microscope with SoRa optics under identical exposure/laser settings across groups. Quantification was performed in Fiji: nuclear-to-cytoplasmic ratios were computed from user-defined nuclear masks, and overlap analyses were derived from binary masks of the indicated channels (area fraction of overlap per cell).

### Antibodies.

Antibodies against GCLC and GCLM were gifts from Terry Kavanagh (University of Washington, Seattle, Washington, USA). A custom rabbit polyclonal anti-LC3B was generated against the N-terminal 14 aa of human LC3B plus cysteine (PSEKTFKQRRTFEQC). Commercial antibodies and sources are listed in Supplemental Table 1.

### Isolation of hepatocytes and nonparenchymal cells.

Cell isolation used retrograde, non-recirculating liver perfusion with 0.05% Collagenase IV (Sigma C5138) as previously described (37). Erythrocytes were removed with RBC Lysis Buffer (Sigma R7757). KCs were enriched from the nonparenchymal fraction by Percoll gradient centrifugation (GE 17-0891-01) following published protocols.

### TCGA data acquisition and preprocessing.

Transcriptomic and clinical data for hepatocellular carcinoma (LIHC) and intrahepatic cholangiocarcinoma (CHOL) were obtained from TCGA using the TCGA biolinks R package. RNA-seq data were downloaded as HTSeq-derived gene expression quantification files generated using the STAR workflow.

Only primary tumor samples (TCGA barcode “TP”) and matched normal tissues (“NT”) were included in downstream analyses. Gene expression values were extracted as TPM and log_2_-transformed [log_2_(TPM + 1)] prior to analysis. Ensembl gene identifiers were mapped to official gene symbols using the org.Hs.eg.db annotation database, and genes without valid symbols or duplicated mappings were removed.

Clinical annotations including tumor stage (American Joint Committee on Cancer pathologic stage), tumor grade, vital status, and OS time were retrieved from TCGA clinical metadata and curated for consistency across cohorts.

### Definition of TFEB-CLEAR network and YAP–SOX9 gene signatures.

A curated TFEB-CLEAR network signature was assembled from established targets involved in lysosomal biogenesis, autophagy, and endolysosomal trafficking, including lysosomal membrane proteins (for example, LAMP1/2), v-ATPase subunits, cathepsins and other lysosomal hydrolases, core autophagy regulators, and MiT/TFE family transcription factors (TFEB, TFE3, MITF). A YAP–SOX9 progenitor-like signature was compiled from published liver cancer and ductal/progenitor studies and included canonical YAP targets, SOX9/Notch pathway components, cholangiocyte markers, stem-like HCC markers, and proliferation-associated genes. A hepatocyte differentiation signature was defined using established hepatocyte transcription factors, metabolic enzymes, and liver-enriched secretory genes. Only genes present in the combined TCGA expression matrix were retained.

### TFEB activity scoring by ssGSEA.

TFEB transcriptional activity was quantified using ssGSEA implemented in the gene set variation analysis package. Log_2_-transformed TPM expression matrices were used as input, and ssGSEA enrichment scores were computed for each tumor sample using the TFEB-CLEAR network signature. Tumors were stratified into TFEB-high, TFEB-intermediate, and TFEB-low groups using tertiles of the TFEB ssGSEA score distribution.

### Correlation analysis.

Pearson’s correlation coefficients were calculated between TFEB ssGSEA scores and expression of individual genes across tumor samples. Correlation values were used for visualization and descriptive purposes.

### Heatmap visualization.

*Z*-score–normalized expression of selected TFEB-CLEAR, YAP–SOX9 progenitor, and hepatocyte marker genes was visualized using ComplexHeatmap. Samples were ordered by TFEB group and descending TFEB ssGSEA score. Genes were grouped by functional category, with hierarchical clustering performed within each gene group to preserve biological structure. Heatmaps were annotated with cancer type (LIHC versus CHOL), TFEB group, tumor stage, and tumor grade, and color scales were centered at 0.

### Tumor stage and grade association analyses.

To evaluate the relationship between TFEB expression or TFEB activity and tumor progression, samples were grouped by AJCC stage and tumor grade. For statistical robustness, advanced stages (stage III and IV) were combined when appropriate.

Differences in expression or ssGSEA scores between normal tissue and tumor stages were assessed using the Wilcoxon rank-sum test. Data are presented as box plots with individual sample points overlaid.

### Survival analysis.

OS was defined as the time from diagnosis to death or last follow-up. Kaplan-Meier survival curves were generated using the survival packages. Statistical significance was assessed using the log-rank test, and hazard-independent survival differences are reported.

### Bioinformatics statistical analysis and software.

All analyses were performed in R (version ≥4.2). Statistical tests were 2-sided, and *P* values less than 0.05 were considered statistically significant unless otherwise stated. Data visualization was performed using ggplot2, Complex Heatmap, and associated R packages.

### Microarrays.

TMAs comprising normal liver, HCC, and intrahepatic cholangiocarcinoma cores were obtained from Biomax (LV2089a, LV2089; BC03118b). Slides were processed for IHC or immunofluorescence staining for CK19, HePar-1 and TFEB using the protocol above. Staining intensity was quantified in ImageJ; each plotted point corresponds to a single core. Sample sizes were *n* = 16, 3, 63, 110 for normal and tumor stages I–III on LV2089 and HCC stage I, *n* = 2; stage II, *n* = 80; stage III, *n* = 31; stage IV, *n* = 7; CCA stage I, *n* = 21; stage II, *n* = 26; stage III, *n* = 15; stage IV, *n* = 13. Mixed carcinoma, *n* = 9 on LV2089a.

### Statistics.

Unless indicated otherwise, results are presented as mean ± SEM. Comparisons between 2 groups used 2-sided Student’s *t* tests; multigroup comparisons used 1-way ANOVA with Tukey’s multiple comparisons test. Multiple-comparison control used Benjamini-Hochberg where applicable. Significance was set at *P* < 0.05 (or *q* < 0.05 for FDR-controlled analyses). Exact *n*, statistical tests, and effect sizes are detailed in the figure legends.

### Study approval.

All procedures were approved by the Institutional Animal Care and Use Committee of the University of Kansas Medical Center (IACUC protocol: IPROTO2024-387).

### Data availability.

The RNA-seq data have been deposited in NCBI’s GEO (GSE318879) and are publicly available. All values underlying graphed data are available in the Supporting Data Values file.

## Author contributions

WXD conceived and supervised the project. CZ, XC, SNW, AMD, A Bajracharya, MH, ND, WL, and HMN performed experiments and analyzed the data. A Ballabio provided TFEB^fl/fl^ mice and revised the manuscript. LM contributed bioinformatic, single-cell, and TCGA analysis. KU and MS contributed GalNAc-siRNA treatment. HMN and WXD conceived and designed the experiments. WL and CZ analyzed the RNA-seq data. CZ and WXD analyzed data and wrote the manuscript.

## Conflict of interest

A Ballabio is cofounder and shareholder of CASMA Therapeutics.

## Funding support

This work is the result of NIH funding, in whole or in part, and is subject to the NIH Public Access Policy. Through acceptance of this federal funding, the NIH has been given a right to make the work publicly available in PubMed Central.

- NIH National Institute on Alcohol Abuse and Alcoholism awards R37 AA020518, R21 AA030617 (WXD).
- NIH National Institute of Diabetes and Digestive and Kidney Diseases R01 DK124612 and R01 DK106540 (WL), R01 DK134737 (HMN).
- NIH National Institute of Environmental Health Sciences R01 ES033623 (WL).
- KU Cancer Center William Warner Abercrombie endowment (WXD).

## Supplementary Material

Supplemental data

Unedited blot and gel images

Supporting data values

## Figures and Tables

**Figure 1:**
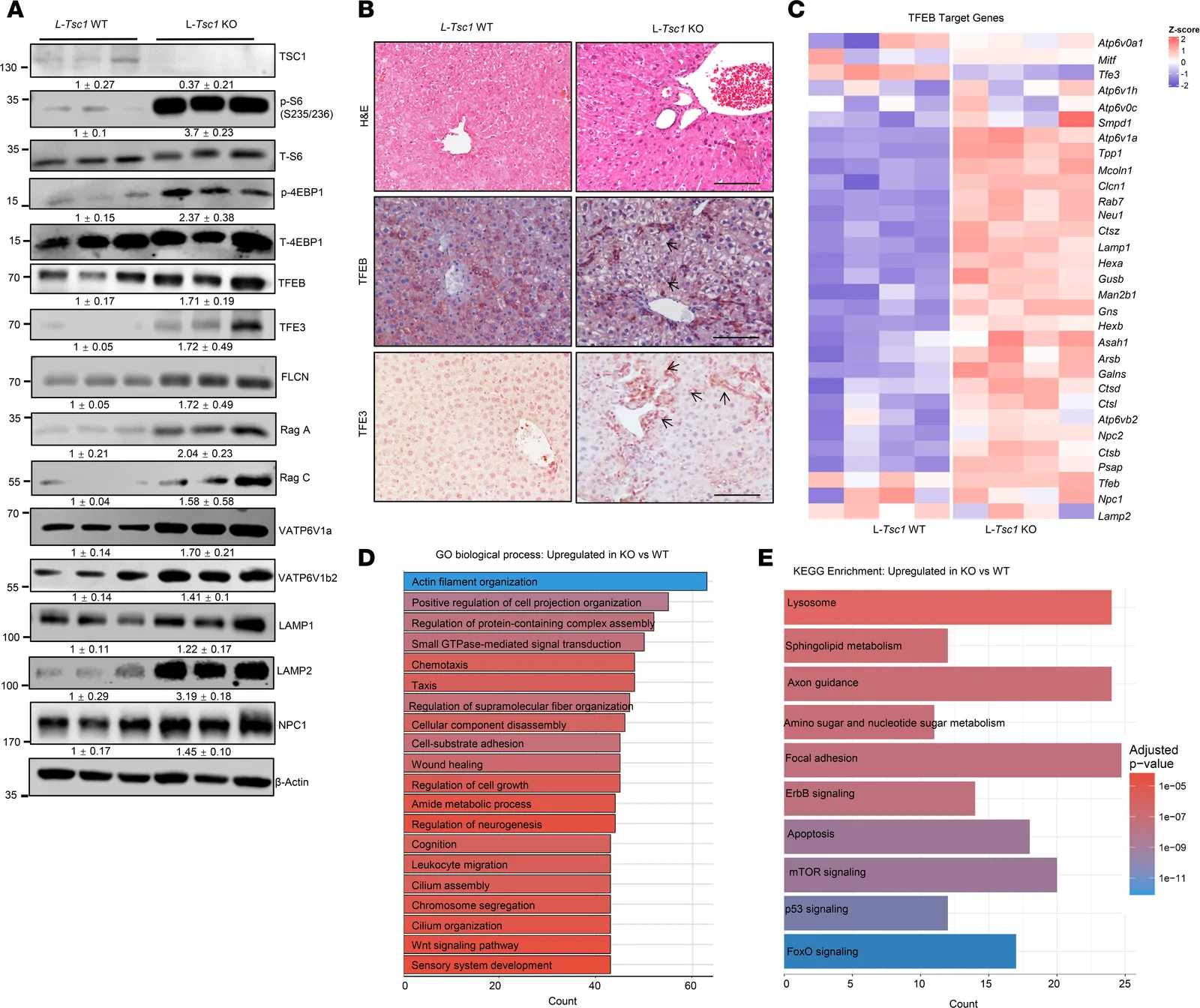
Loss of hepatic TSC1 activates TFEB and increases ductular–fibrotic remodeling in the mouse livers despite mTORC1 activation. (**A**) Immunoblotting of total liver lysates from 2-month-old mice of the indicated genotypes. (**B**) Representative images of H&E and IHC staining for TFEB and TFE3 of 2-month-old mice. Scale bars, 100 μm. (**C**) Heatmap of TFEB target genes from bulk RNA-seq (2-month-old livers; *n* = 4 per genotype). (**D**) GO (Biological Process) enrichment for genes upregulated in *Tsc1*-KO versus WT livers. Dot size indicates the number of genes contributing to each term; color denotes adjusted *P* values (Benjamini-Hochberg). (**E**) KEGG pathway enrichment of genes upregulated in *Tsc1*-KO versus WT. Bar size reflects gene count, and color indicates adjusted *P* values (Benjamini-Hochberg).

**Figure 2:**
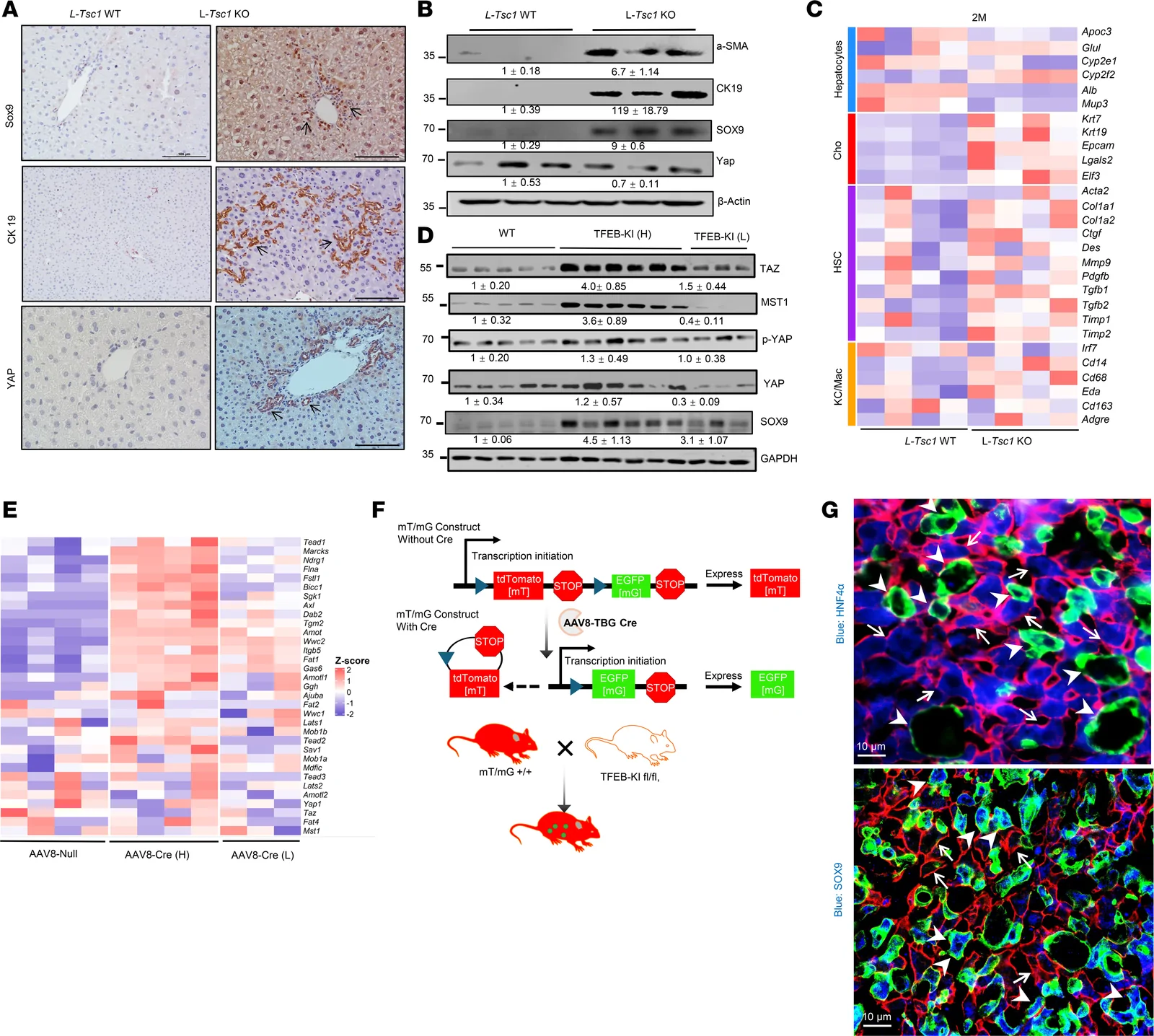
TFEB overexpression in hepatocytes is sufficient to induce hepatocyte transdifferentiation to hepatic progenitor/biliary epithelial cells. (**A**) Representative images of IHC staining of liver sections for CK19, SOX9, and YAP (2-month-old mice of the indicated genotypes). Scale bars, 100 μm. (**B**) Immunoblot analysis of total liver lysates from 2-month-old male mice. (**C**) Heatmap of cell type marker signatures from bulk RNA-seq of livers (*n* = 4 per genotype per time point of 2-month-old mice). (**D**) Immunoblot analysis of livers from WT and TFEB-KI mice 2 weeks after AAV8-TBG-Cre injection at the indicated doses (high dose: 2 × 10¹^0^ or low dose: 5 × 10^9^ GC/mouse). (**E**) Heatmap of YAP pathway genes from bulk RNA-seq of the same cohorts. (**F**) Schematic of mT/mG reporter activation by Cre. (**G**) Two-month-old Rosa26-LSL-Tfeb-3xFlag mT/mG reporter mice were injected with AAV8-TBG-Cre (1 × 10¹^0^ GC/mouse for 1 month). Cryosectioned/frozen liver tissues were subjected to fluorescence staining for SOX9 and HNF4α. Representative fluorescence images are shown. Arrows indicate Cre-negative cells (red membrane label, no TFEB overexpression), which are HNF4α-positive/SOX9-negative hepatocytes; arrowheads indicate Cre-positive cells (green membrane label, TFEB overexpression), which are SOX9-positive/HNF4α-negative cells.

**Figure 3:**
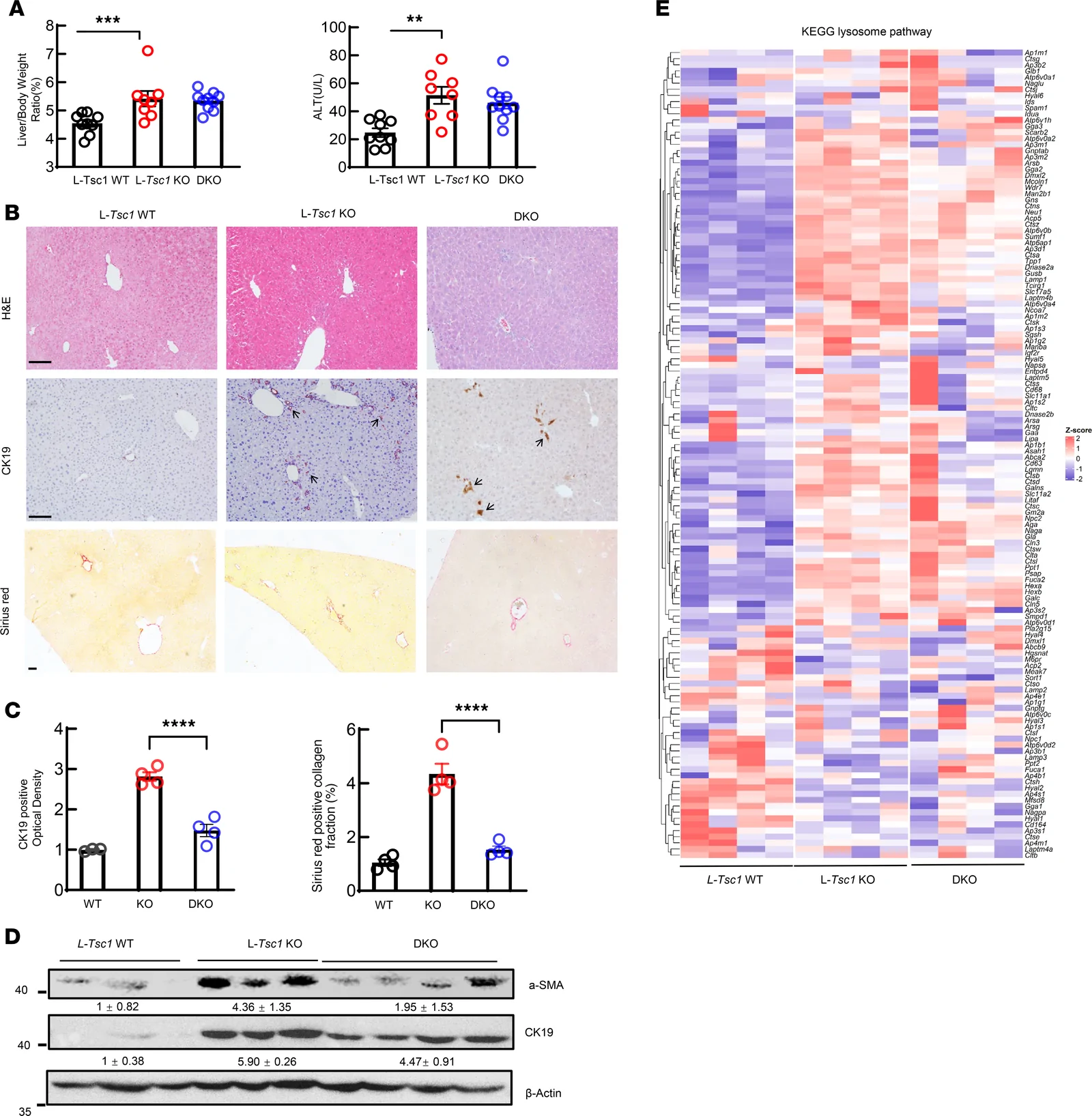
TFEB deletion partially reverses ductular–fibrotic remodeling in TSC1-deficient livers. (**A**) Liver weight–to–body weight (LW/BW) ratio and serum ALT in 2-month-old mice of the indicated genotypes. Data are shown as mean ± SEM (*n* = 8–10). **P < 0.01, ***P < 0.001. One-way ANOVA with Tukey’s multiple-comparison test. (**B**) Representative images of H&E, Sirius Red, and IHC staining for CK19 in 2-month-old livers. Scale bars, 100 μm. (**C**) OD for CK19 and Sirius Red collagen fraction was quantified in Fiji/ImageJ, then normalized to WT mean. Data are mean ± SEM (*n* = 4). ****P < 0.0001, 1-way ANOVA with Tukey’s multiple-comparison test. (**D**) Total liver lysates were subjected to immunoblot analysis. Densitometry analysis was quantified in ImageJ and normalized to the WT mean. (**E**) KEGG lysosomal pathway enrichment of genes in L-*Tsc1* *Tfeb*–DKO and L-*Tsc1*–KO versus WT.

**Figure 4:**
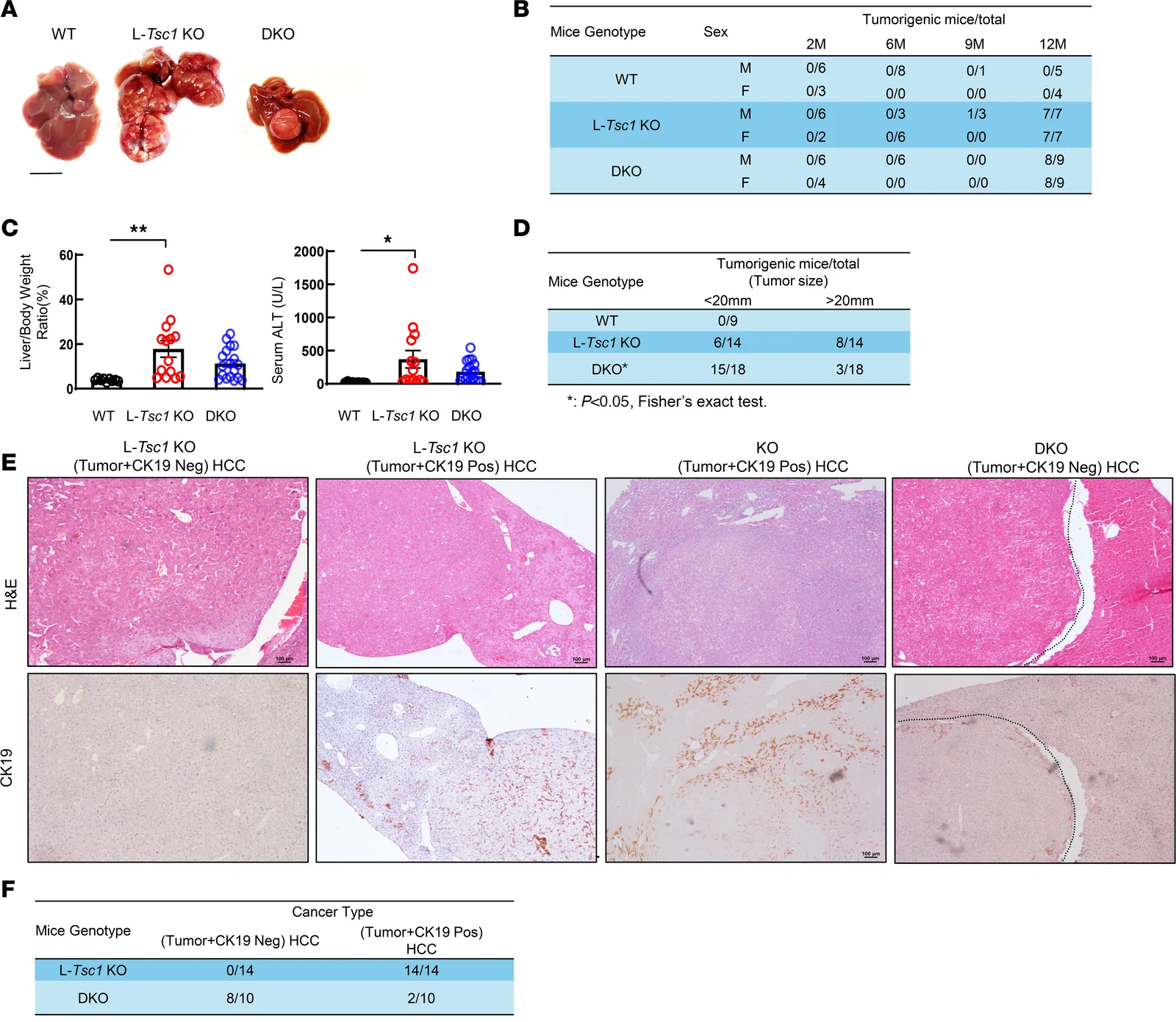
Increased tumor burden and SOX9-positive HCC with increased CK19-positive ductular cell infiltration in L-*Tsc1*–deficient mice are driven by TFEB activation. (**A**) Representative gross images of livers from 12-month-old mice of the indicated genotypes. (**B**) Summary of tumor formation. (**C**) Quantification of LW/BW ratio, serum ALT. Data are mean ± SEM (*n* = 9–18). *P < 0.05, **P < 0.01. One-way ANOVA with Tukey’s multiple-comparison test. (**D**) Tumor burden and size summary. *P < 0.05, Fisher’s exact test. (**E**) Representative images of H&E and CK19 IHC staining from the same tumors illustrating (tumor+CK19 negative) HCC or (tumor+CK19 positive) HCC. Scale bars, 100 μm. (**F**) Summary of the distribution of tumor types in L-*Tsc1*–KO and L-*Tsc1 Tfeb*–DKO cohorts.

**Figure 5:**
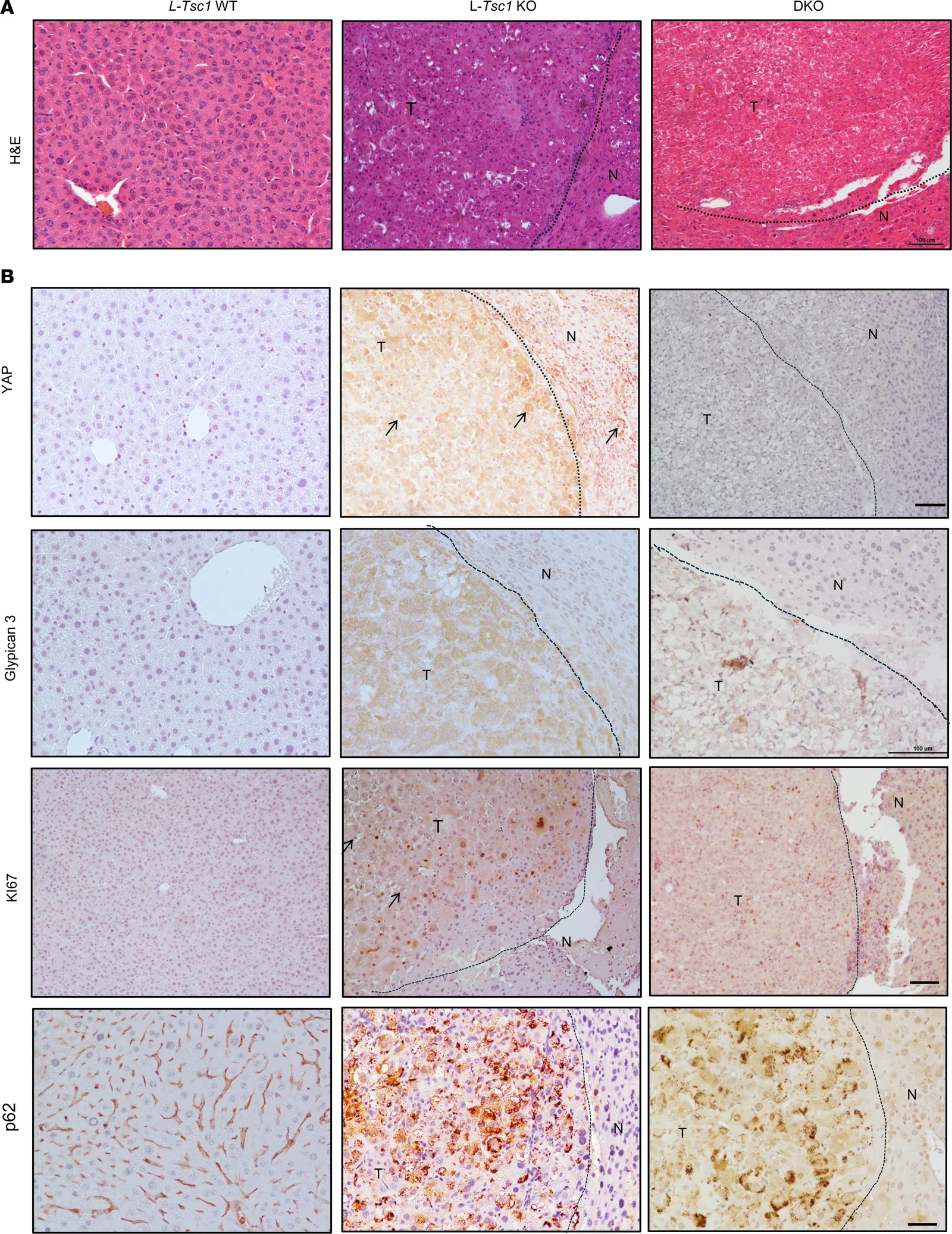
Increased tumor markers in L-*Tsc1*–deficient mice. (**A** and **B**) Representative H&E and IHC staining of liver sections for YAP, glypican-3, Ki67, and p62 from 12-month-old mice of the indicated genotypes. Scale bars, 100 μm. The arrows in panel **B** indicate representative YAP-positive cells (YAP row) and representative Ki67-positive proliferating nuclei (Ki67 row) within the tumor region. N, nontumor; T, tumor.

**Figure 6:**
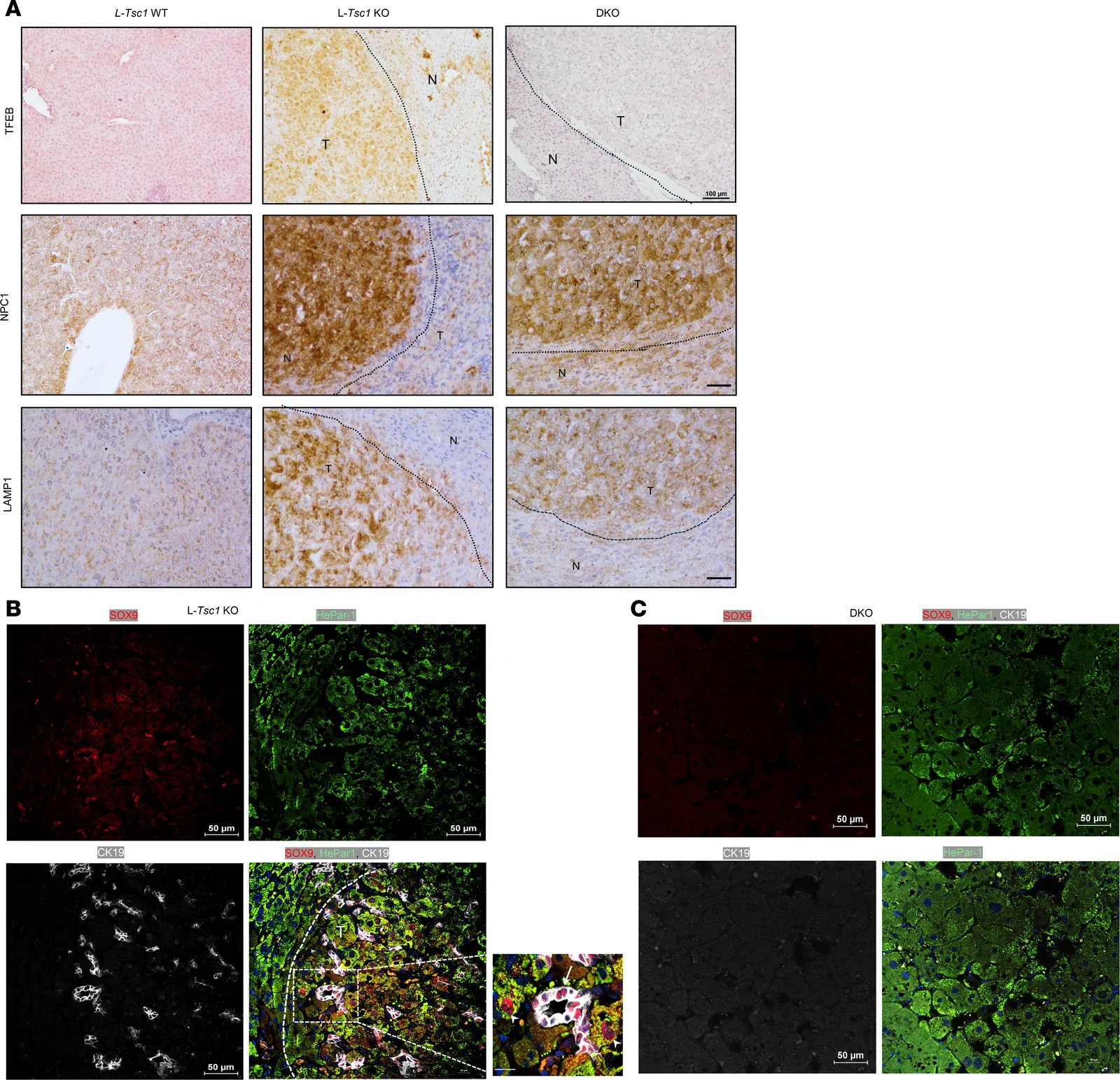
Increased SOX9-positive HCC with increased CK19-positive ductular cell infiltration in L-*Tsc1*–deficient mice is driven by TFEB activation. (**A**) Representative IHC staining of liver sections for TFEB, NPC1, and LAMP1 from 12-month-old mice of the indicated genotypes. Scale bars, 100 μm. (**B**) Representative confocal immunofluorescence images showing HepPar-1 (green), SOX9 (red), and CK19 (white) in 12-month-old L-*Tsc1*–KO liver (**B**) and L-*Tsc1* *Tfeb*–DKO liver (**C**). Scale bars, 50 μm; inserted image 15 µm. In the inset, the arrow indicates a representative CK19/SOX9/TFEB triple-positive ductular/progenitor cell, and the arrowhead indicates an adjacent HepPar-1–positive HCC cell shown for comparison.

**Figure 7:**
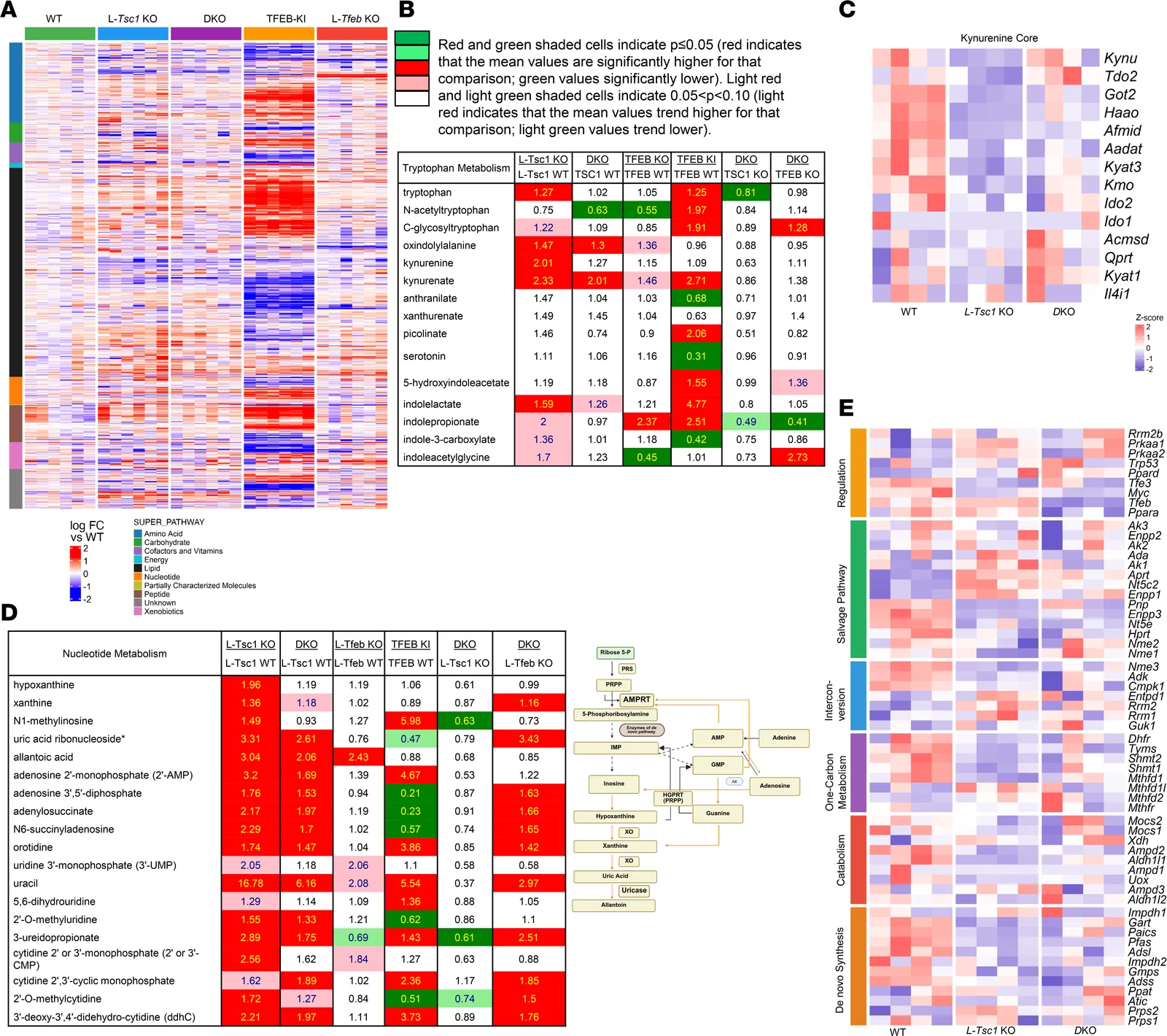
TFEB deletion partially restores metabolic homeostasis in TSC1-deficient livers. (**A**) Global metabolite heatmap (2-month livers; *n* = 6) comparing L-*Tsc1*–KO, L-*Tsc1* *Tfeb*–DKO, TFEB overexpression (TFEB-KI), and L-*Tfeb*–KO with WT mice. (**B**) Heatmap of tryptophan metabolism of metabolomics analysis of indicated mouse liver tissues (*n* = 6). (**C**) Heatmap of kynurenine core genes from bulk RNA-seq of livers at 2 months (*n* = 4). (**D**) Heatmap of nucleotide metabolites (*n* = 6). (**E**) Heatmap of purine pathway gene expression from bulk RNA-seq (*n* = 4).

**Figure 8:**
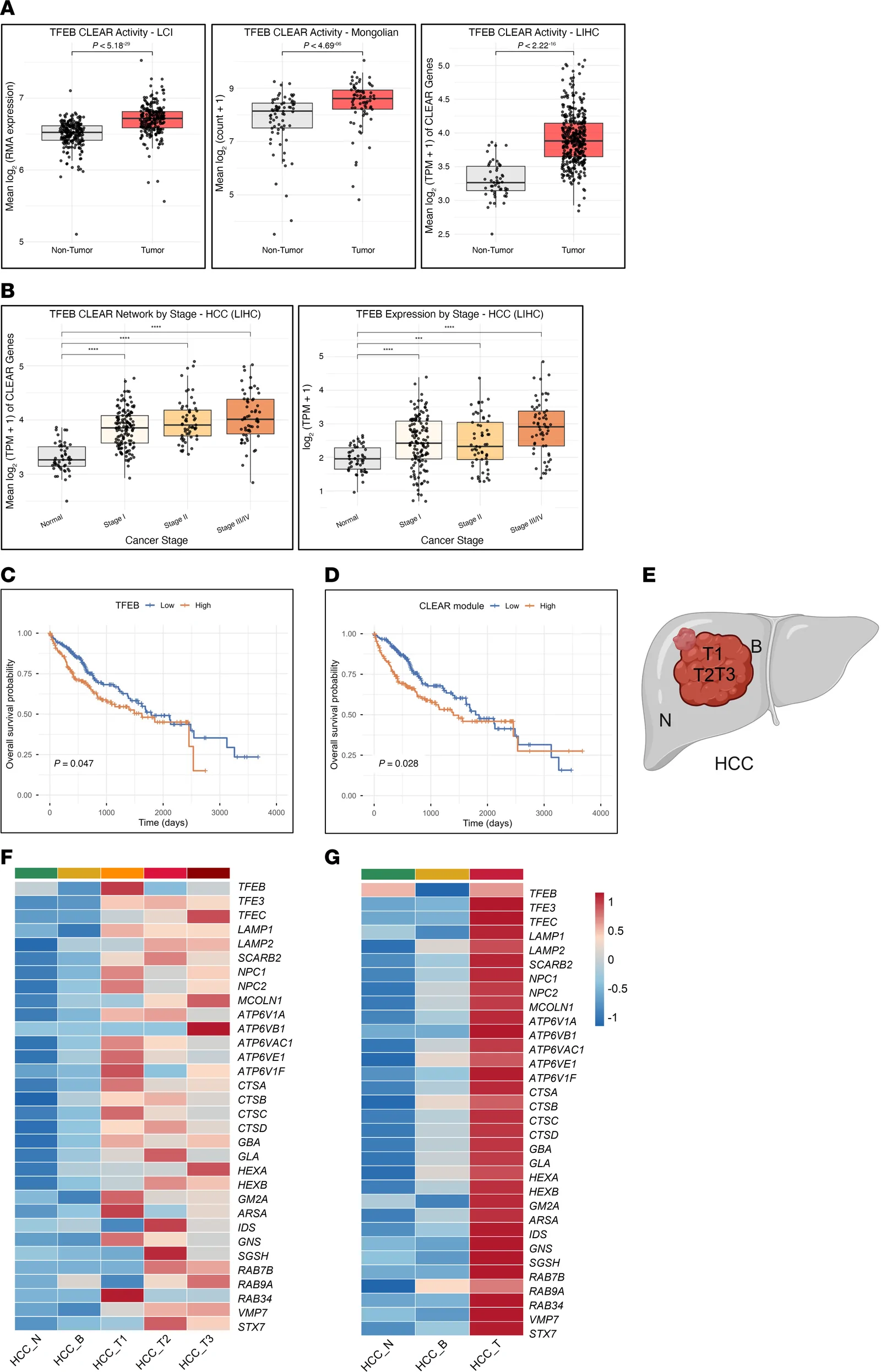
TFEB-CLEAR network activity is elevated in human liver cancer and is associated with stage, survival, spatial tumor regions, and malignant cells. (**A**) TFEB-CLEAR network activity (mean expression/module score of curated CLEAR genes) in nontumor liver versus tumor across independent human cohorts, including the LCI dataset, the Mongolia dataset, and TCGA-LIHC (HCC). *P* values were determined by 2-sided Wilcoxon’s rank-sum test. (**B**) TFEB-CLEAR network score and TFEB expression stratified by clinical stage in TCGA-LIHC. ***P < 0.001, ****P < 0.0001. *P* values were determined by Kruskal-Wallis test with multiple-comparison adjustment. (**C**) TFEB expression in each TCGA-LIHC tumor sample was transformed as log2(TPM + 1). Patients were then dichotomized into TFEB-high and TFEB-low groups using the cohort median TFEB expression value as the cutoff, and Kaplan-Meier OS of TCGA-LIHC patients was stratified by TFEB expression (high versus low). (**D**) Log2(TPM + 1)–transformed expression values were standardized separately for each gene, and the CLEAR score was calculated as the mean of the genewise *z* scores across a curated gene panel related to lysosomal biogenesis, autophagy, and intracellular trafficking. Patients were subsequently classified into CLEAR-high and CLEAR-low groups using the cohort median CLEAR score as the cutoff. Kaplan-Meier overall survival of TCGA-LIHC patients stratified by ssGSEA-based TFEB-CLEAR module activity (high versus low). *P* value was determined by log-rank test. (**E**) Schematic of multiregional sampling in the single-cell dataset (GSE189903) study, including adjacent normal tissue (N), tumor border (B), and 3 tumor cores (T1, T2, and T3). (**F**) Heatmap showing regional expression of TFEB-CLEAR network genes across N, B, and individual tumor cores (T1–T3) in GSE189903. Values represent averaged (*z*-score–scaled) expression per region. (**G**) Same as **F**, with tumor cores T1–T3 merged into a single tumor group to summarize tumor versus border versus normal differences. TPM, transcripts per million; ssGSEA, single-sample gene set enrichment analysis; OS, overall survival.

**Figure 9:**
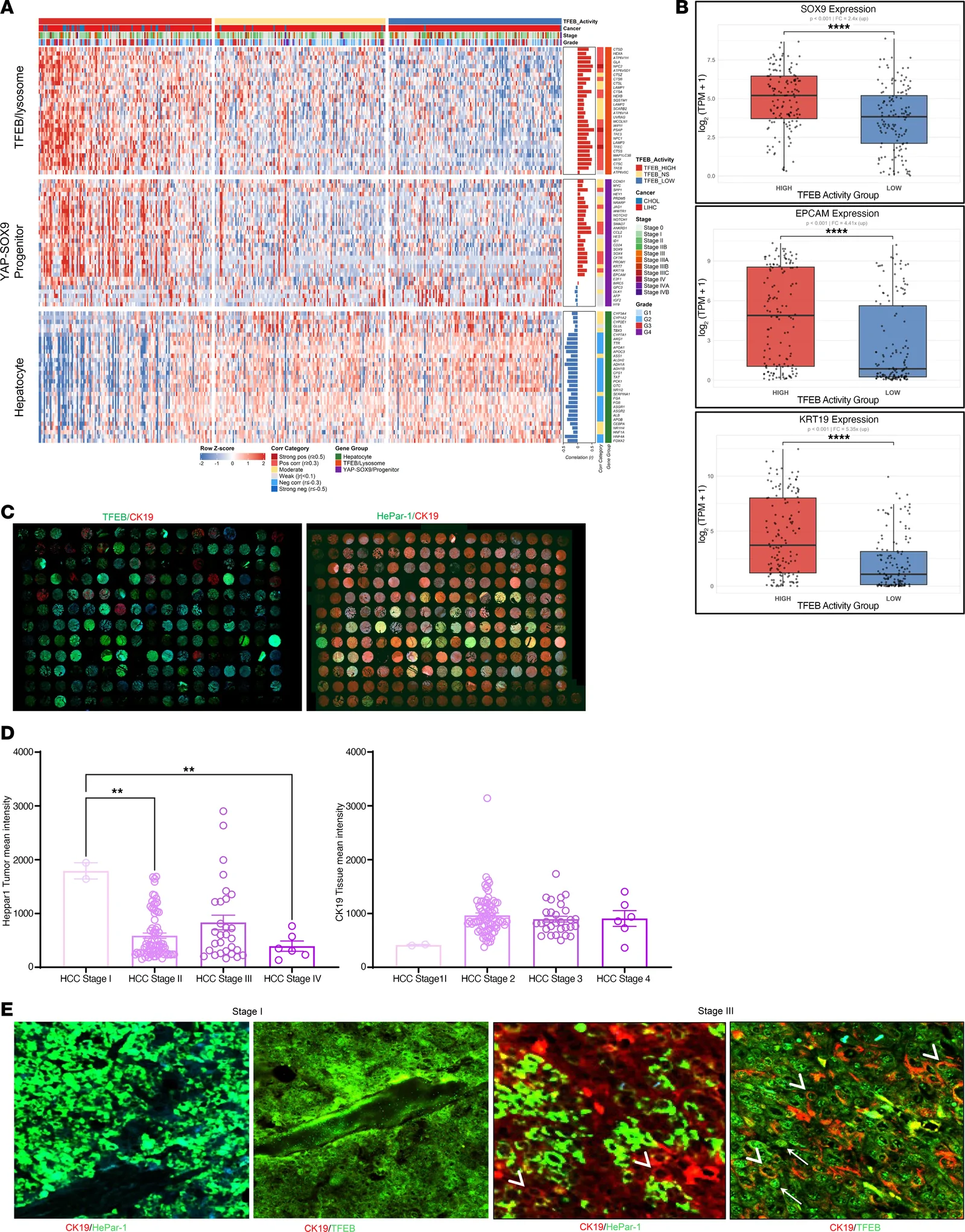
TFEB transcriptional activity is associated with progenitor-like programs in liver cancers. (**A**) Heatmap analysis of z-score–normalized expression for selected genes from the TFEB-CLEAR network, YAP–SOX9 progenitor-like signature, and hepatocyte differentiation signature across the combined TCGA-LIHC and TCGA-CHOL cohorts. Samples were ordered by TFEB ssGSEA score and stratified into TFEB-high, TFEB-intermediate, and TFEB-low groups using tertiles of the TFEB ssGSEA distribution. Top annotations indicate TFEB group, cancer type (LIHC vs. CHOL), tumor stage, and grade. Pearson’s correlation coefficients shown at right reflect association between individual gene expression and TFEB ssGSEA scores. (**B**) Expression of representative progenitor/biliary markers (SOX9, EPCAM, KRT19) in TFEB-high versus TFEB-low tumors. Expression of SOX9, EPCAM, and KRT19 (log2[TPM+1]) was compared between groups using a 2-sided Wilcoxon’s rank-sum test. ****P < 0.0001. (**C**) Immunofluorescence costaining of TFEB (green) with CK19 (red) and HepPar-1 (green) with CK19 (red) on liver carcinoma tissue microarray, containing 75 cases of intrahepatic cholangiocarcinoma, 120 HCC, and 9 mixed carcinomas. (**D**) The mean intensity of a single core per case was quantified using Qupath. **P < 0.01, 1-way ANOVA with Tukey’s multiple-comparison test. (**E**) Representative image of costaining of TFEB (green) with CK19 (red) and HepPar-1 (green) with CK19 (red) on HCC.

## Version 1 08/06/2026

In-Press Preview

## Version 2 10/01/2026

Electronic publication
