# Supplementary material for: Noncanonical mTORC1-TFEB activation promotes hepatocyte plasticity and high-grade malignancy in hepatocellular carcinoma
Source: J Clin Invest. 2026 Aug 6;136(19):e206334. doi: 10.1172/JCI206334 (PMC13626827; doi:10.1172/JCI206334)
Supplement: Supplementary file 1 [file jci-136-206334-s116.pdf]

Non-canonical mTORC1-TFEB activation promotes hepatocyte plasticity and high-grade malignancy in hepatocellular carcinoma

Chen Zhang[1], Xiaojuan Chao[1], Sha Neisha Williams[1], Xiaoli Wei[1], Anthony Michael DiGirolamo[1], Alisha Bajracharya[1], Lichun Ma[2,3], Ming Huang[1], Nicholas Dunn[1], Wanqing Liu[4,5], Kaito Ueda[6], Masayuki Sugimoto[6], Andrea Ballabio[7, 8, 9], Hong-Min Ni[1] and Wen-Xing Ding[1,10*]

Supplemental Figure Legend

Supplementary Figure 1. Changes of hepatic TFEB and its target genes in Day 7 old L-*Tsc1* KO mouse livers

Total liver lysates from Day 7 old L-*Tsc*1 KO mouse livers were subjected to western blot analysis. Densitometry analysis was performed using ImageJ and normalized to the WT mean (set to 1.0); values are shown beneath each lane (mean ± SD).

Supplementary Figure 2. Increased CK19-positive cells with nuclear TFEB in L-*Tsc1* KO mouse livers

(A) Immunofluorescence for TFEB (green) and CK19(red) in 2-month-old L-*Tsc1* KO mouse liver. The lower panels show enlarged photographs of the boxed regions. Arrow denotes cells with increased nuclear TFEB. Scale bar, 10 μm.

Supplementary Figure 3. TFEB overexpression perturbs hepatocyte identity and promotes the development of ductular features.

(A) Schematic of the conditional Rosa26 Tfeb-3×Flag knock-in allele (Tfeb-3×Flag^fs/fs), in which a loxP-flanked STOP cassette blocks CAG promoter–driven TFEB-3×Flag until Cre recombination. (B) Representative gross liver morphology 2 weeks after tail-vein injection of AAV8-TBG-Null or AAV8-TBG-Cre at the indicated doses (2×10^10 vs 5×10^9 genome copies (GC)/mouse). (C) Serum biochemistry and liver/body-weight ratio 2 weeks after injection. ALT, liver/body-weight ratio. Data are mean ± SE. (n=6-9). ** p<0.01, *p< 0.001. Ordinary one-way ANOVA with Turkey's multiple comparison test. (D)ALP, total bile acids, triglycerides, and cholesterol in WT and TFEB-KI mice receiving low- or high-dose AAV8-TBG-Cre. Data are shown as mean ± SEM with individual mice. Data are mean ± SE. (n=3-6). ** p<0.01, *p< 0.001. Ordinary one-way ANOVA with Turkey's multiple comparison test. (E) Heatmap of "Liver cell cluster" marker matrix summarizing lineage signatures from bulk RNA-seq of livers in A. Values are variance-stabilized and Z-scored per gene. Note the dose-dependent loss of hepatocyte markers and gain of cholangiocyte markers with TFEB overexpression.

(F) Immunoblot of liver lysates demonstrating dose-dependent TFEB-3×Flag overexpression and associated changes in TFE3, lysosomal markers (LAMP1, LAMP2, V-ATP6V1A, V-ATP6V1B2), mitochondrial regulator PGC1α, autophagy markers LC3 and p62 and hepatocyte markers HNF4α P1, HNF4α P2, the P1/P2 ratio, CYP2E1, and glutamine synthetase, together with biliary/ductular and fibrotic markers EPCAM, CK19, and α-SMA; GAPDH serves as loading control. Densitometry analysis was performed using ImageJ and normalized to the WT mean (set to 1.0); values are shown beneath each lane (mean ± SD). (G) Representative liver

sections from WT and TFEB-KI mice 2 weeks after high- or low-dose AAV8-TBG-Cre. Shown are H&E, Sirius Red, and IHC for HNF4α P1, glutamine synthetase and YAP. Scale bars, 100 μm.

Supplementary Figure 4. Progressive liver injury and fibrosis in 6-month-old L-*Tsc1* KO mice.
(A) Representative histological and immunohistochemical staining of liver sections from 6-month-old mice of the indicated genotypes. Representative images of hematoxylin and eosin (H&E), Sirius Red, and immunostaining for CK19 and SOX9 are shown. scale bars, 100 μm. (B) Heatmap of cell identity genes. Z-scores are shown per gene with hierarchical clustering. (C) Representative histological and immunohistochemical staining of liver sections from 12-month-old mice of the indicated genotypes. Shown are hematoxylin and eosin (H&E) and immunostaining for CK19. scale bars, 100 μm.

Supplementary Figure 5. Long-term TFEB overexpression induces biliary cystic disease.
(A) Gross liver morphology from WT (10 months) and TFEB-KI mice treated with low-dose AAV8-TBG-Cre (5×10^9 GC/mouse, collected at 8–10 months). AAV-Null–treated TFEB-KI mice (5×10^9 GC/mouse, 8–10 months) serve as vector controls. (B) Liver-to-body-weight ratio and serum ALT activity in WT and TFEB-KI mice (5×10^9 GC/mouse, 8–10 months). Data are mean ± SEM; Data are mean ± SEM (WT=3, Low dose=12). (C) Representative liver images of retrograde ink injection of the biliary tree in WT (6 months) and TFEB-KI mice (1×10^{10} GC/mouse, 6 months). (D) Representative liver sections from WT and TFEB-KI mice (5×10^9 GC/mouse, 8–10 months). Representative images of H&E, Sirus Red, YAP, SOX9 and TFEB staining, highlighting multifocal biliary cysts lined by $YAP^+/SOX9^+$ epithelium in TFEB-KI livers are shown. Scale bars, 100 μm. (E) Heatmaps of bile-acid metabolism genes and cyst formation genes from bulk RNA-seq of livers with indicated genotypes. Values are variance-stabilized and Z-scored per gene.

Supplementary Figure 6. Progressive liver injury and fibrosis in 6-month-old L-*Tsc1* KO mice.
(A) Liver/body weight ratio and serum alanine aminotransferase (ALT) levels in 6-month-old mice of the indicated genotypes. Data represent mean ± SE. (n=6-9). ** p<0.01, ***p< 0.001. Ordinary one-way ANOVA with Tukey's multiple comparison test. (B) Representative images of histological and immunohistochemical staining of liver sections from 6-month-old mice of the indicated genotypes. Shown are hematoxylin and eosin (H&E), Sirius Red, and immunostaining for CK19. scale bars, 100 μm. (C) Immunoblot analysis of total liver lysates from 6-month-old mice of the indicated genotypes. Densitometry analysis was performed using ImageJ and normalized to the WT mean (set to 1.0); values are shown beneath each lane (mean ± SD).

Supplementary Figure 7. Loss of TFEB loss reshapes TSC1 deficiency-driven transcriptomic programs.
(A) Principal-component analysis (PCA) of bulk RNA-seq from livers of 2-month-old mice (WT, L-*Tsc1* KO, and L-*Tsc1,Tfeb* DKO; n=4 per genotype). Each point is one sample; axes show variance explained. (B) Heatmap of TFEB CLEAR network genes. Z-scores are shown per gene with hierarchical clustering. (C) Heatmap of liver cell identity cluster genes. Z-scores are shown per gene with hierarchical clustering. (D) Immunoblot analysis of total liver lysates from 2-month-old mice of the indicated genotypes. (E) Immunoblot analysis of total liver lysates from 6-month-old mice of the indicated genotypes. Densitometry analysis was performed using ImageJ and normalized to the WT mean (set to 1.0); values are shown beneath each lane (mean ± SD).

Supplementary Figure 8. Hepatic TFEB directs interacts with YAP and SOPX9 and loss of TFEB attenuates gene expression of fibrogenic growth factors.
(A) Two-months-old Rosa26 Tfeb-3×Flag^fs/fs) mice were injected with AAV8-TBG-Null or AAV8-TBG-Cre (2×10^10 genome copies (GC)/mouse) for 2 weeks and total liver lysates were subjected to immunoprecipitation with anti-Flag antibody. Original lysates (input) and the immunoprecipitation samples were then subjected to immunoblot analyses. Representative images are shown. (B) Heatmap analysis of RNAseq dataset for fibrogenic growth factors. Z-scores are shown per gene with hierarchical clustering.

Supplementary Figure 9. YAP/TAZ drives ductular reactions/progenitor cells and liver fibrosis in L-*Tsc1*-KO mice.
Two-months-old L-*Tsc1* KO mice were injected with GalNAc-siRNA control and GalNAc-siRNA Yap/Taz (10 mg/kg, subcutaneously) for 10 days. (A) Representative images of H&E, Sirius red and IHC staining for CK19 and SOX9 are shown. scale bars, 100 μm. (B) Serum ALT levels were quantified and staining intensity of CK19, Sirius red and SOX9 were quantified using Fiji. Data are mean ± SE (n=3-4). * $p<0.05$ ** $p<0.01$, as determined by an unpaired, 2-tailed Welch's t test. (C) Total liver lysates were subjected to western blot analysis. Densitometry analysis was performed using ImageJ and normalized to the WT mean (set to 1.0); values are shown beneath each lane (mean ± SD).

Supplementary Figure 10. TFEB drives CK19-positive cells in HCC of L-*Tsc1*-KO mice.
(A) Representative images of H&E and matched CK19 IHC staining from the same tumors illustrating (Tumor+CK19 Negative) HCC or (Tumor+CK19 Positive) in L-*Tsc1* KO HCC; scale bars, 100 μm. The H&E image of the HCC+CK19+ cells (L-*Tsc1* KO) and the CK19 image were from a similar area of Figure 4E at higher magnification. (B) Representative images of H&E and matched CK19 IHC staining from the same tumors illustrating (Tumor+CK19 Negative) HCC in L-*Tsc1, Tfeb* DKO HCC. scale bars, 100 μm.

Supplementary Figure 11. TFEB drives hepatocyte transdifferentiation to BEC-like cells in HCC.
(A) Representative images of IHC staining for PCNA, r-H2A and KEAP1 from the same tumors illustrating (Tumor+CK19 Negative) HCC or (Tumor+CK19 Positive) in L-*Tsc1* KO HCC; scale bars, 100 μm. (B) Representative images of confocal microscopy analysis of immunofluorescence of TFEB (green) and CK19(red) staining in 12-month-old L-*Tsc1* KO liver. Top shows non-tumor and bottom shows tumor area. Right panels are images with higher magnification of boxed regions. Arrows denote nuclear TFEB.

Supplementary Figure 12. TFEB promotes tumorigenesis-associated protein expression in L-*Tsc1* KO livers.
(A, B) Immunoblot analysis of total liver lysates from 12-month-old mice of the indicated genotypes. Band intensities were quantified in ImageJ and normalized to the WT mean (set to 1.0); values are shown beneath each lane (mean ± SD).

Supplementary Figure 13. Overexpression of constitutive active RagC (RagC^{CA}) enhances TFEB–lysosome coupling and partially relocate TFEB into the cytosol in L-*Tsc1* KO mouse liver.
(A) Schematic experiment design. (B) Liver/body weight ratio of indicated genotypes (n=3-6).
(C) Representative images of H&E staining of livers from L-*Tsc1* KO mice injected with Ad-Null

or Ad-RagC^{CA} (5x10 ^{9} PFU/ mouse, iv) for 2 weeks. scale bars, 100 µm. (D) Confocal microscopy analysis of immunofluorescence staining for TFEB (red), CK19 (white), and nuclei (Hoechst 33342, blue) in L-*Tsc1* KO livers expressing Ad-Null or Ad-RagC^{CA},; scale bars, 20 µm. (E) Immunoblot analysis of total liver lysates from L-*Tsc1* KO mice injected in *vivo* with Ad-Null or Ad-RagC^{CA} and paired WT mice injected in *vivo* with Ad-Null. (F) Confocal microscopy analysis of immunofluorescence staining for TFEB (red), LAMP1 (green), and nuclei (DAPI, blue) in primary WT hepatocytes and L-*Tsc1* KO hepatocytes infected with livers expressing Ad-Null or Ad-RagC^{CA} (5 MOI for 24 hours). (G) Quantification of panel (F) using Fiji, showing TFEB nuclear/cytosol ratio and TFEB lysosome translocation score; each dot represents a single cell. Data are pooled from three independent experiments (n=38 cells total). * $p<0.05$, **** $p<0.0001$, Ordinary one-way ANOVA with Tukey's multiple comparison test.

Supplementary Figure 14. TFEB reshapes the metabolomic landscape in L-*Tsc1* KO mouse liver.
(A) Principal component analysis (PCA) of untargeted liver metabolomics from 2-month-old mice of the indicated genotypes (WT, L-*Tsc1* KO, L-*Tsc1, Tfeb* DKO, hepatocyte TFEB overexpression (TFEB-KI), and L-*Tfeb* KO; each point represents one mouse (n=6 in each group). (B) Pathway-level summary of metabolites altered in L-*Tsc1* KO versus WT liver. Dots represent metabolic pathways, with red indicating net increases and green indicating net decreases in KO; dot size reflects the number of significantly changed metabolites within each pathway. (C) The impact of TFEB on the L-*Tsc1* KO metabolomic phenotypes. Each dot represents a pathway, plotted as the percentage of metabolites whose levels are shifted toward WT ("rescued by TFEB loss", green) or further away from WT ("worsened by TFEB loss", red) in L-*Tsc1,Tfeb* DKO relative to *L-Tsc1* KO.

Supplementary Figure 15. TFEB activation on γ-glutamyl amino-acid and ceramide related metabolism.
(A) Heatmap analysis of γ-glutamyl amino-acid metabolites from the global metabolomics dataset in 2-month-old mice (n = 6 per group); each row is an individual metabolite, displayed as log fold-change relative to WT. (B) A simplified schematic of the γ-glutamyl amino-acid pathway. (C) Heatmap analysis of ceramides metabolites from the global metabolomics dataset in 2-month-old mice (n = 6 per group); each row is an individual metabolite, displayed as log fold-change relative to WT. (D) Heatmap of a ceramide metabolism pathway from bulk RNA-seq (2M; n = 4 per group); values are variance-stabilized and shown as module Z-scores.

Supplementary Figure 16. Impact of TFEB on Polyamine metabolism.
(A) Heatmap analysis of polyamine metabolites from liver metabolomics in 2-month-old mice (n = 6 per group). Values are shown as fold-change relative to the indicated control for each comparison. Schematic of the core polyamine pathway from arginine to putrescine, spermidine, spermine, and 1,3-diaminopropane. (B) Heatmap of a polyamine-metabolism from bulk RNA-seq (2M; n = 4 per group); values are Z-scores.

Supplementary Figure 17. Impact of TFEB on BMP metabolism.
(A) Heatmap analysis of bis(monoacylglycero)phosphate (BMP) species metabolites from liver metabolomics in 2-month-old mice (n = 6 per group). Values are shown as fold-change relative to the indicated control for each comparison. (B) Heatmap of BMP-metabolism gene from bulk RNA-seq (2M; n = 4 per group); values are Z-scores.

Supplementary Figure 18. Impact of TFEB on TCA cycle metabolism.

(A) Heatmap analysis of TCA cycle from liver metabolomics in 2-month-old mice (n = 6 per group). Values are shown as fold-change relative to the indicated control for each comparison. Schematic of the core TCA cycle pathway. (B) Heatmap of a curated TCA cycle gene module from bulk RNA-seq (2M; n = 4 per group); values are Z-scores.

Supplementary Figure 19. Impact of TFEB on bile acid metabolism.
Heatmap analysis of bile acid metabolites from liver metabolomics in 2-month-old mice (n = 6 per group). Values are shown as fold-change relative to the indicated control for each comparison.

Supplementary Figure 20. Heterogeneity of TFEB staining in human liver specimens.
(A) Representative images of the immunohistochemistry staining for TFEB from LV2089 of human HCC tissue microarray cores spanning malignant tumor stages I–IV. Staining intensity was quantified in Qupath, each dot represents the optical density of a single core (B). LV2089 contains 16 normal liver tissues and 176 HCC cases, including HCC stage I, n = 3; stage II, n = 63; and stage III, n = 110. The array also contains 4 mixed hepatocellular carcinoma cases and 12 bile duct adenocarcinomas. * $p<0.05$, Ordinary one-way ANOVA with Tukey's multiple comparison test.. (C) The IHC staining for p-S6 indicating mTORC1 activity was done as described previously (PMID: 34710483) and reanalyzed with TFEB staining. The Summary of samples with high, normal, and low intensity of TFEB and p-S6 staining. Based on analyzable cores used for staining quantification, cases were grouped as normal, n = 16; malignant tumor stage I, n = 3; stage II, n = 66; and stage III, n = 114.

Supplementary Figure 21. Heterogeneity of TFEB staining in human liver specimens.
(A) Images of TFEB immunohistochemistry staining on an independent human liver tissue microarray (LV2089a) in human HCC tissue microarray cores spanning malignant tumor stages I–IV. (B) Staining intensity was quantified in Qupath; each dot represents the optical density of a single core. *** $p<0.001$, **** $p<0.0001$, Ordinary one-way ANOVA with Tukey's multiple comparison test.. (C) Representative high magnification images of H&E and IHC staining for TFEB showing different Stage HCC.

Supplementary Figure 22. Heterogeneity of TFEB staining in human CCA liver specimens.
(A) Images of TFEB immunohistochemistry staining on an independent human liver tissue microarray (LV2089a) in human CCA tissue microarray cores spanning malignant tumor stages I–IV. (B) Staining intensity was quantified in Qupath; each dot represents the optical density of a single core. ** $p<0.01$, *** $p<0.001$, **** $p<0.0001$, Ordinary one-way ANOVA with Tukey's multiple comparison test. (C) Representative high magnification images of H&E and IHC staining for TFEB showing different Stage CCA are shown.

sFigure 1

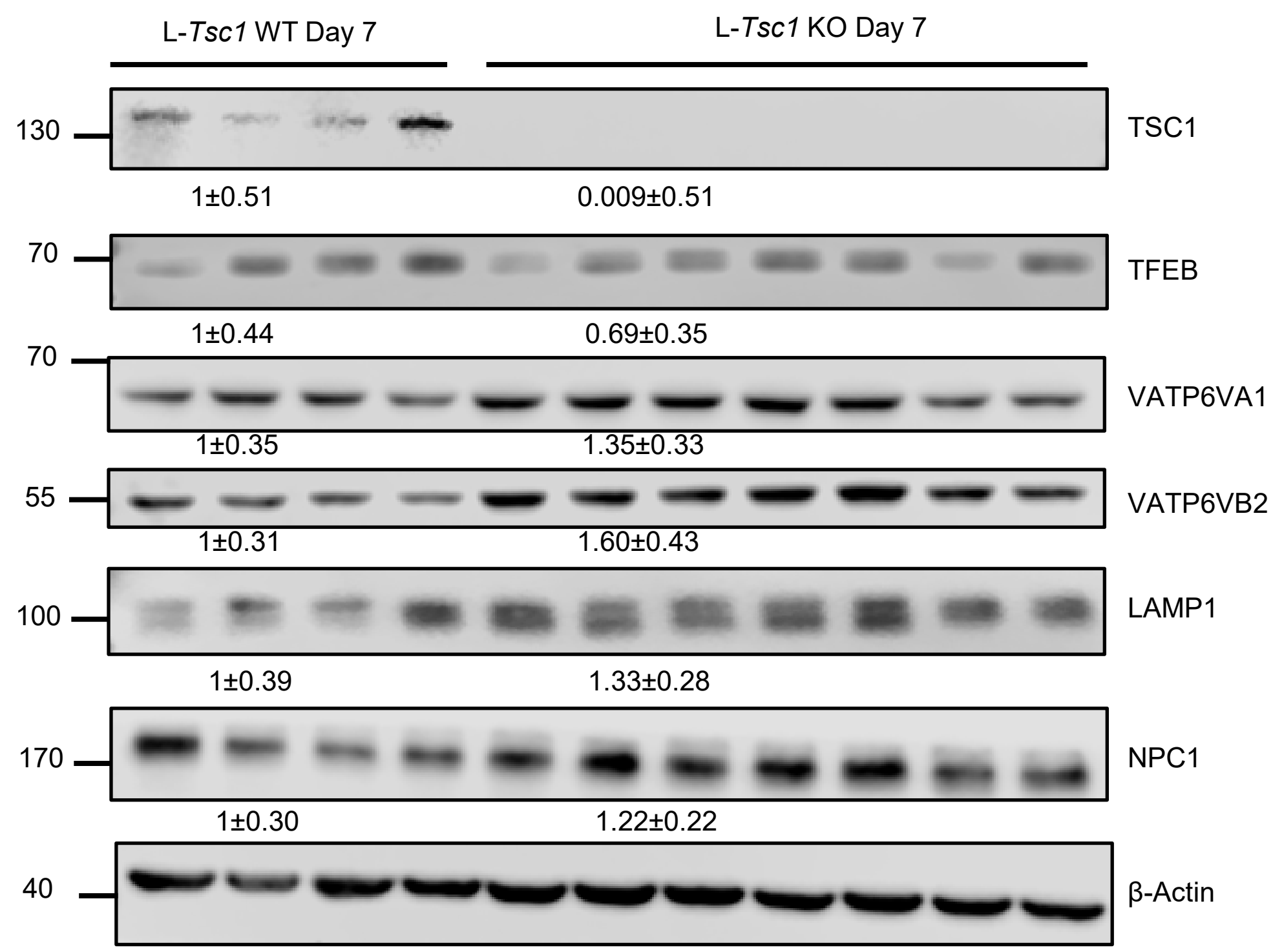

L-*Tsc1* WT Day 7
L-*Tsc1* KO Day 7
130
TSC1
1±0.51
0.009±0.51
70
TFEB
1±0.44
0.69±0.35
70
VATP6VA1
1±0.35
1.35±0.33
55
VATP6VB2
1±0.31
1.60±0.43
100
LAMP1
1±0.39
1.33±0.28
170
NPC1
1±0.30
1.22±0.22
40
β-Actin

sFigure 2

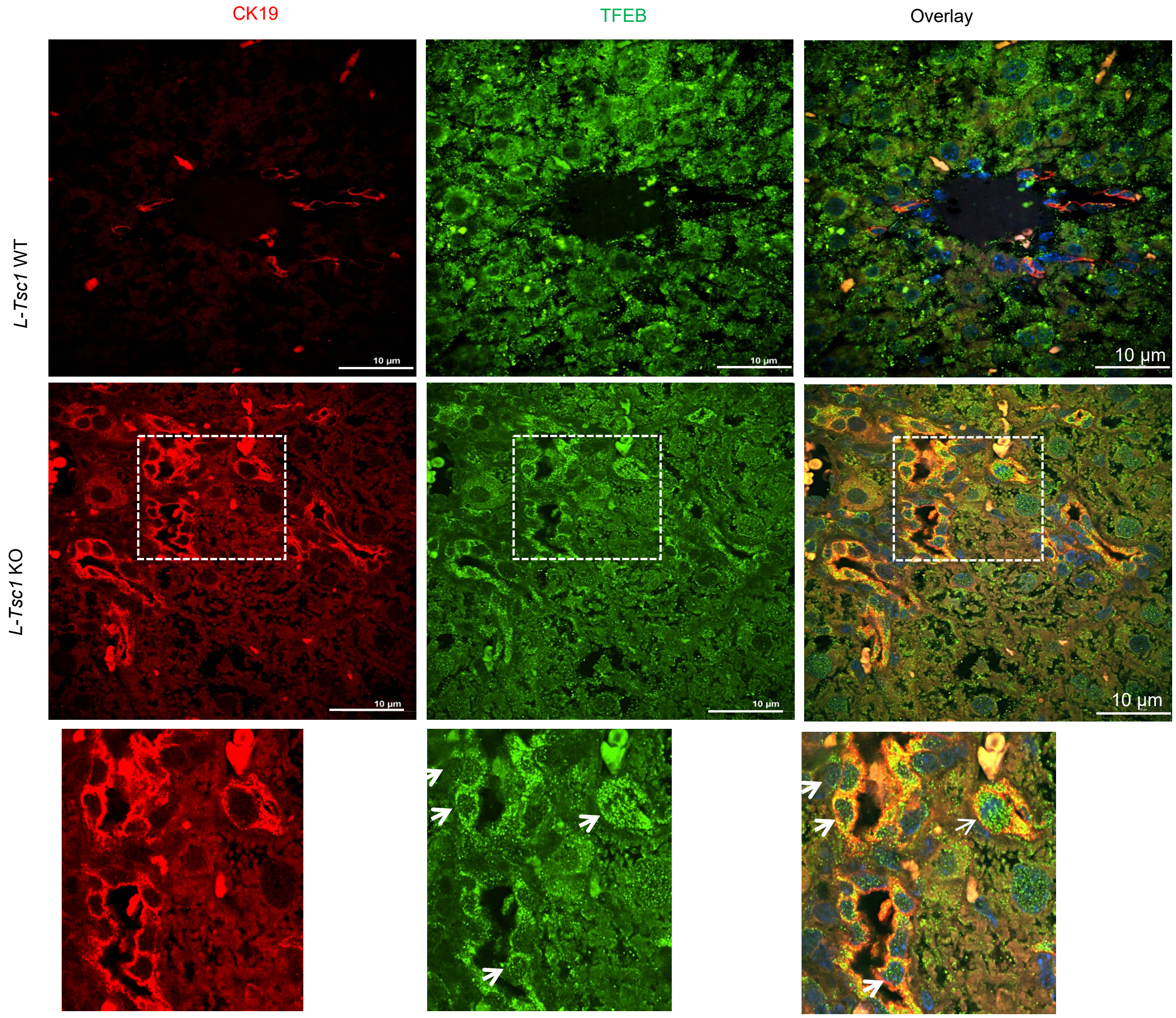

sFigure 3

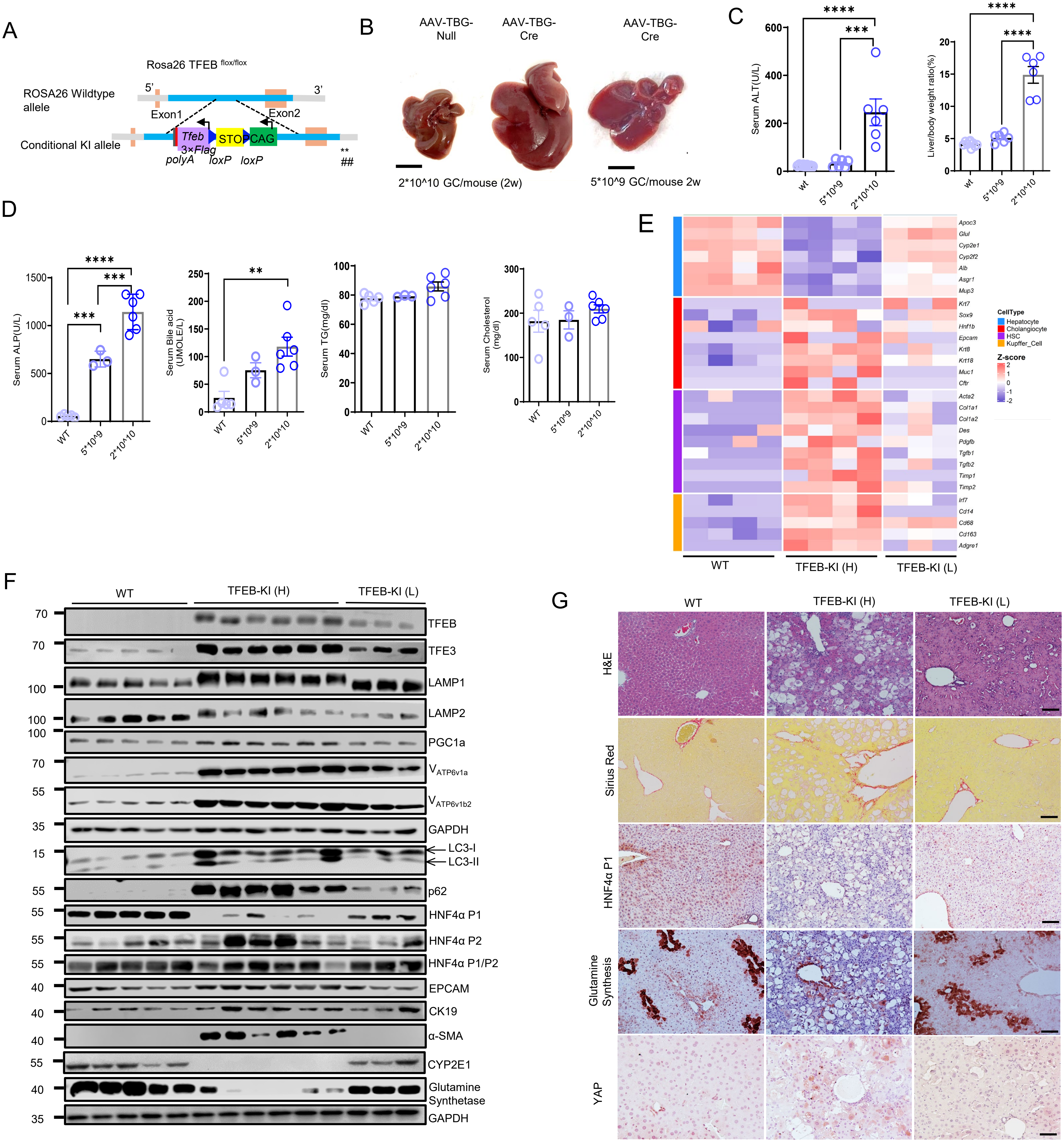
A
Rosa26 TFEB flox/flox
ROSA26 Wildtype allele
Exon1
Exon2
Conditional KI allele
polyA
Tfeb 3×Flag
STOP
CAG
loxP
B
AAV-TBG-Null
AAV-TBG-Cre
2*10^10 GC/mouse (2w)
5*10^9 GC/mouse 2w
C
Serum ALT(U/L)
Liver/body weight ratio(%)
wt
5*10^9
2*10^10
D
Serum ALP(U/L)
Serum Bile acid (UMOLE/L)
Serum TG(mg/dl)
Serum Cholesterol (mg/dl)
WT
E
CellType
Hepatocyte
Cholangiocyte
HSC
Kupffer_Cell
Z-score
TFEB-KI (H)
TFEB-KI (L)
F
TFEB
TFE3
LAMP1
LAMP2
PGC1a
VATP6v1a
VATP6v1b2
GAPDH
LC3-I
LC3-II
p62
HNF4α P1
HNF4α P2
HNF4α P1/P2
EPCAM
CK19
α-SMA
CYP2E1
Glutamine Synthetase
G
H&E
Sirius Red
Glutamine Synthesis
YAP

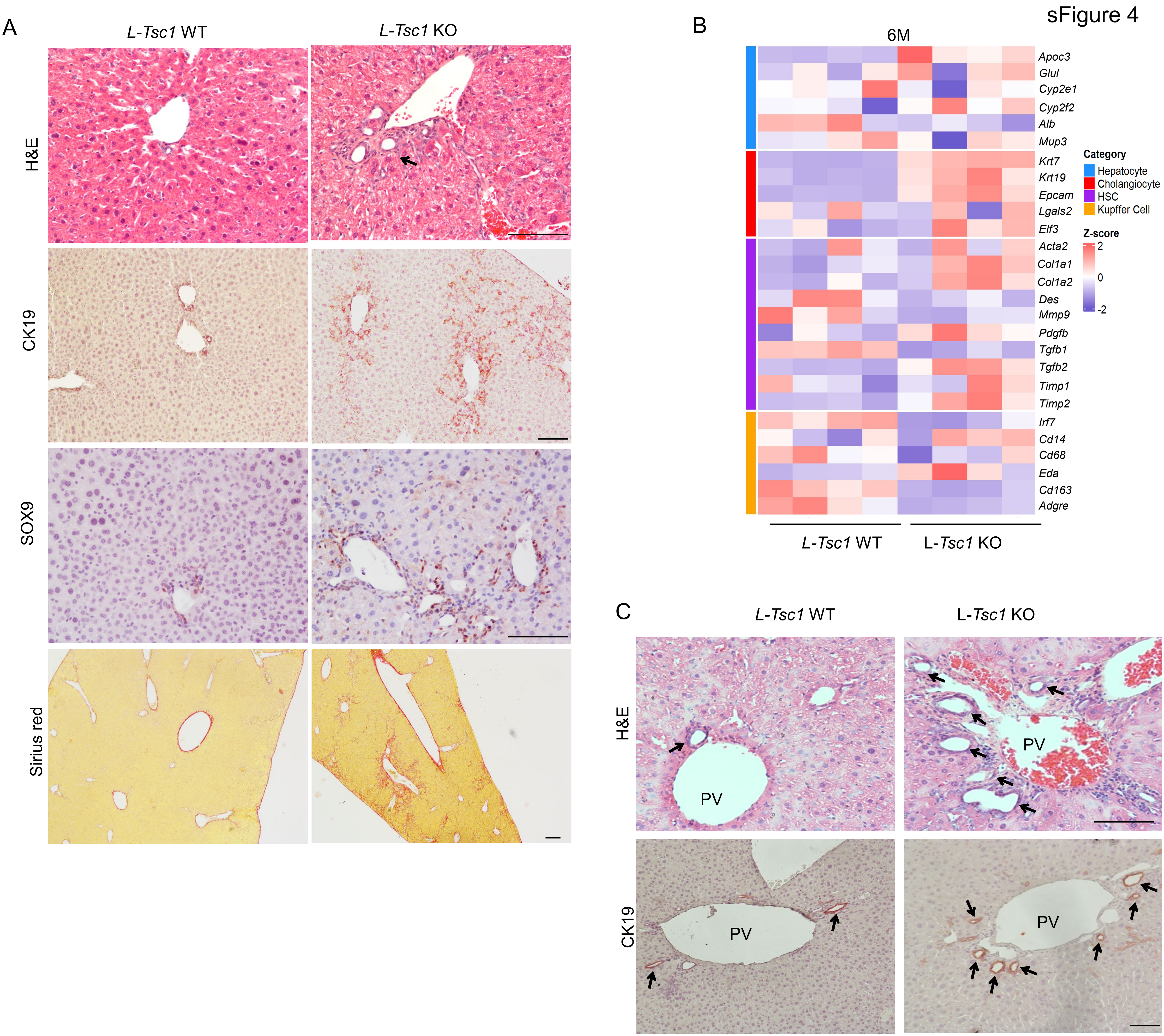
sFigure 4
A
L-Tsc1 WT
L-Tsc1 KO
H&E
CK19
SOX9
Sirius red
B
6M
Apoc3
Glul
Cyp2e1
Cyp2f2
Alb
Mup3
Krt7
Krt19
Epcam
Lgals2
Elf3
Acta2
Col1a1
Col1a2
Des
Mmp9
Pdgfb
Tgfb1
Tgfb2
Timp1
Timp2
Irf7
Cd14
Cd68
Eda
Cd163
Adgre
Category
Hepatocyte
Cholangiocyte
HSC
Kupffer Cell
Z-score
2
0
-2
L-Tsc1 WT
L-Tsc1 KO
C
L-Tsc1 WT
L-Tsc1 KO
H&E
CK19
PV

sFigure 5

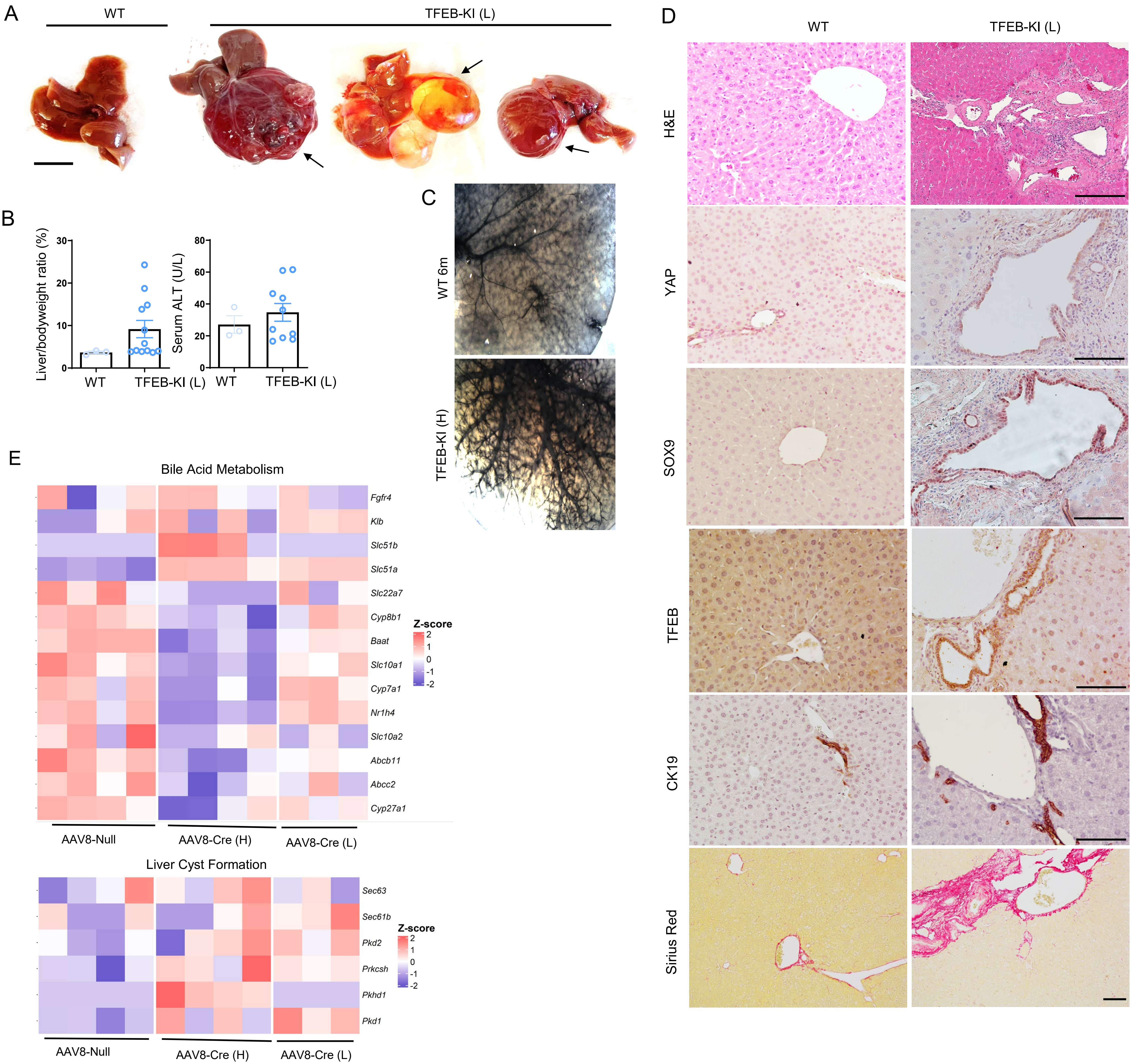
A
WT
TFEB-KI (L)
B
Liver/bodyweight ratio (%)
Serum ALT (U/L)
WT
TFEB-KI (L)
C
WT 6m
TFEB-KI (H)
D
WT
TFEB-KI (L)
H&E
YAP
SOX9
TFEB
CK19
Sirius Red
E
Bile Acid Metabolism
Fgfr4
Klb
Slc51b
Slc51a
Slc22a7
Cyp8b1
Baat
Slc10a1
Cyp7a1
Nr1h4
Slc10a2
Abcb11
Abcc2
Cyp27a1
Z-score
AAV8-Null
AAV8-Cre (H)
AAV8-Cre (L)
Liver Cyst Formation
Sec63
Sec61b
Pkd2
Prkcsh
Pkhd1
Pkd1
Z-score
AAV8-Null
AAV8-Cre (H)
AAV8-Cre (L)

sFigure 6

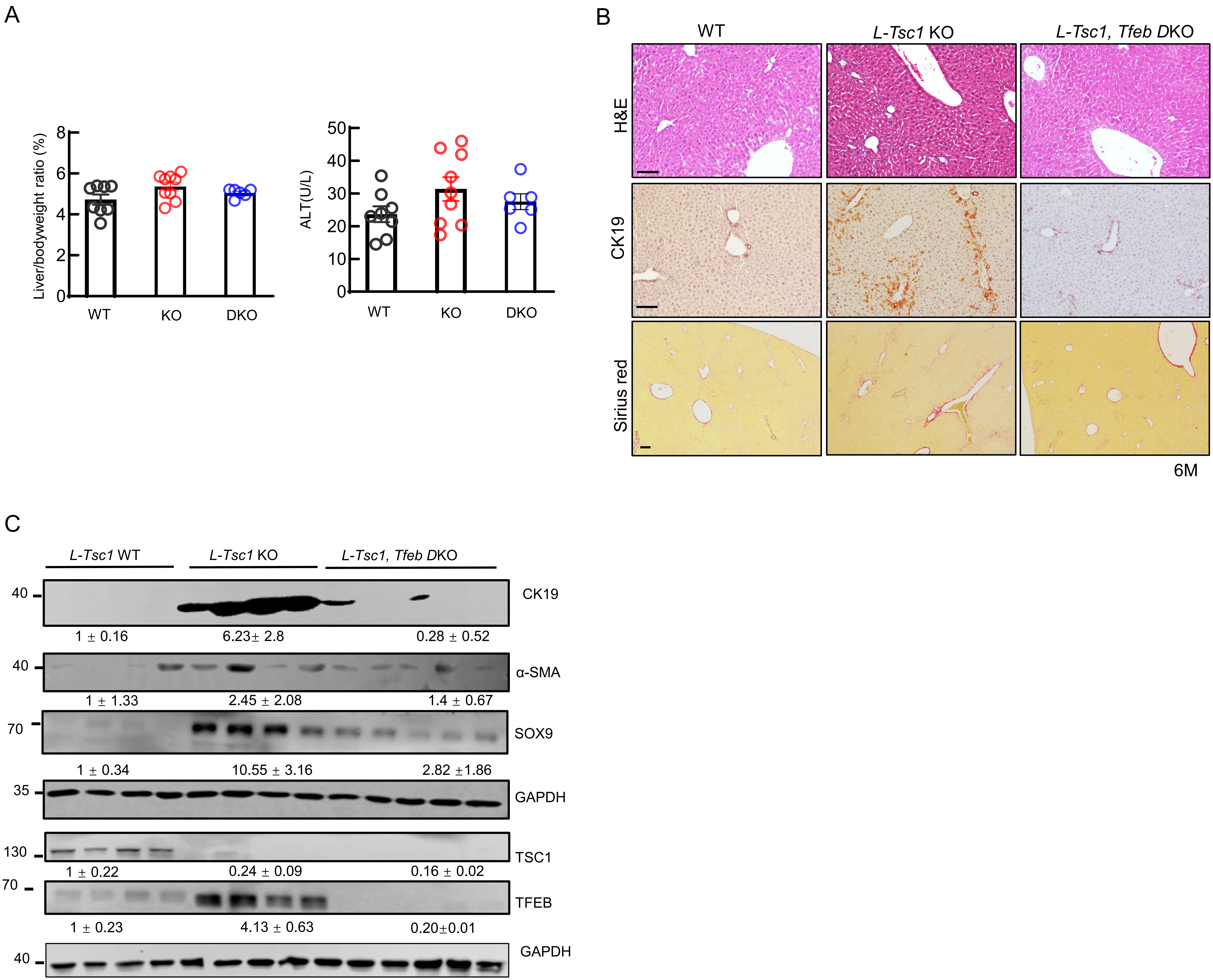

A
Liver/bodyweight ratio (%)
WT
KO
DKO
ALT(U/L)
B
WT
L-Tsc1 KO
L-Tsc1, Tfeb DKO
H&E
CK19
Sirius red
6M
C
L-Tsc1 WT
L-Tsc1 KO
L-Tsc1, Tfeb DKO
CK19
1 ± 0.16
6.23± 2.8
0.28 ± 0.52
α-SMA
1 ± 1.33
2.45 ± 2.08
1.4 ± 0.67
SOX9
1 ± 0.34
10.55 ± 3.16
2.82 ±1.86
GAPDH
TSC1
1 ± 0.22
0.24 ± 0.09
0.16 ± 0.02
TFEB
1 ± 0.23
4.13 ± 0.63
0.20±0.01
GAPDH

sFigure 7

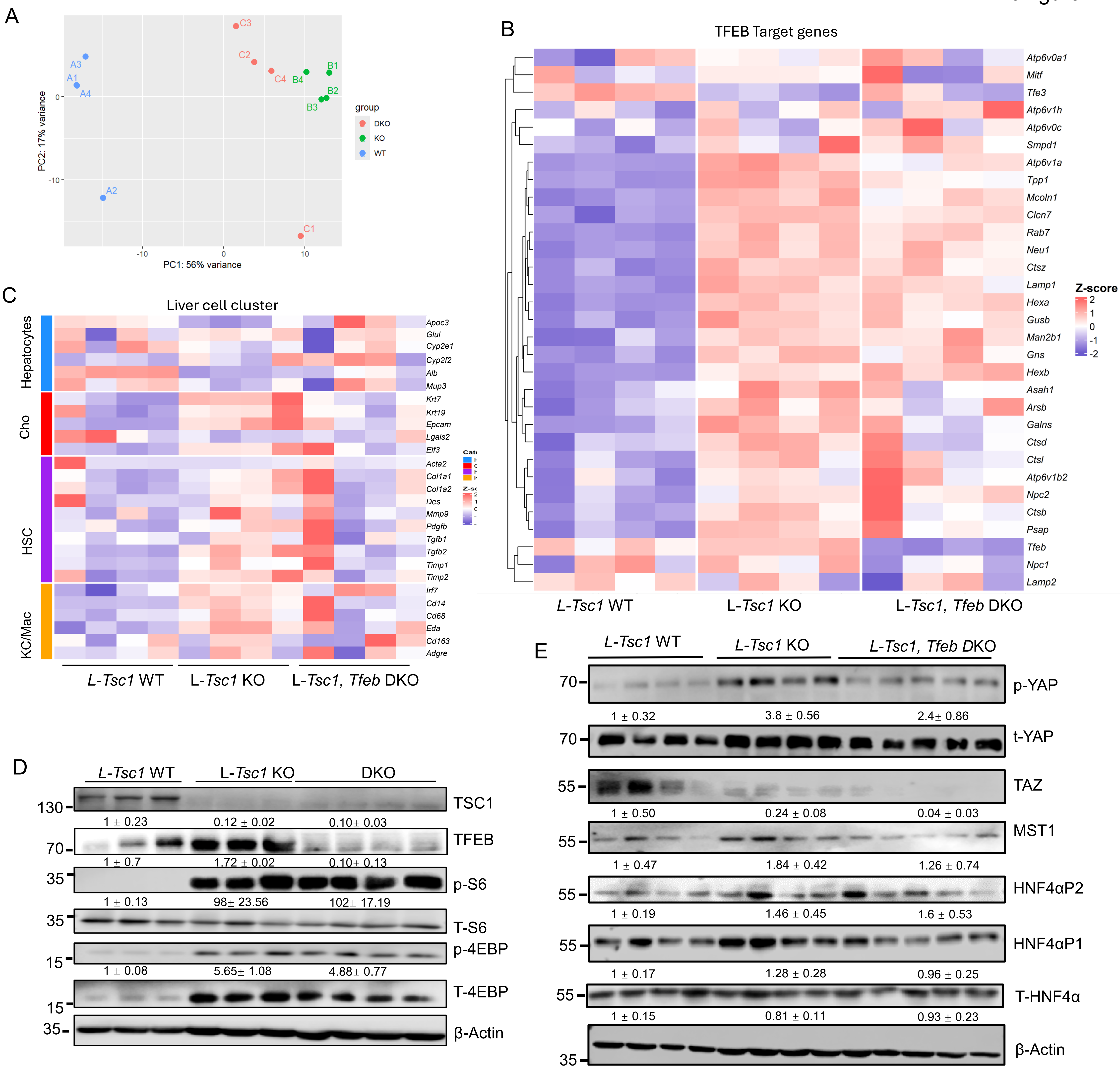
A
C3
C2
C4
B4
B1
B2
B3
A3
A1
A4
A2
C1
PC2: 17% variance
PC1: 56% variance
group
DKO
KO
WT
B
TFEB Target genes
Atp6v0a1
Mitf
Tfe3
Atp6v1h
Atp6v0c
Smpd1
Atp6v1a
Tpp1
Mcoln1
Clcn7
Rab7
Neu1
Ctsz
Lamp1
Hexa
Gusb
Man2b1
Gns
Hexb
Asah1
Arsb
Galns
Ctsd
Ctsl
Atp6v1b2
Npc2
Ctsb
Psap
Tfeb
Npc1
Lamp2
Z-score
L-Tsc1 WT
L-Tsc1 KO
L-Tsc1, Tfeb DKO
C
Liver cell cluster
Hepatocytes
Cho
HSC
KC/Mac
Apoc3
Glul
Cyp2e1
Cyp2f2
Alb
Mup3
Krt7
Krt19
Epcam
Lgals2
Elf3
Acta2
Col1a1
Col1a2
Des
Mmp9
Pdgfb
Tgfb1
Tgfb2
Timp1
Timp2
Irf7
Cd14
Cd68
Eda
Cd163
Adgre
L-Tsc1 WT
L-Tsc1 KO
L-Tsc1, Tfeb DKO
D
L-Tsc1 WT
L-Tsc1 KO
DKO
TSC1
1 ± 0.23
0.12 ± 0.02
0.10± 0.03
TFEB
1 ± 0.7
1.72 ± 0.02
0.10± 0.13
p-S6
1 ± 0.13
98± 23.56
102± 17.19
T-S6
p-4EBP
1 ± 0.08
5.65± 1.08
4.88± 0.77
T-4EBP
β-Actin
E
L-Tsc1 WT
L-Tsc1 KO
L-Tsc1, Tfeb DKO
p-YAP
1 ± 0.32
3.8 ± 0.56
2.4± 0.86
t-YAP
TAZ
1 ± 0.50
0.24 ± 0.08
0.04 ± 0.03
MST1
1 ± 0.47
1.84 ± 0.42
1.26 ± 0.74
HNF4αP2
1 ± 0.19
1.46 ± 0.45
1.6 ± 0.53
HNF4αP1
1 ± 0.17
1.28 ± 0.28
0.96 ± 0.25
T-HNF4α
1 ± 0.15
0.81 ± 0.11
0.93 ± 0.23
β-Actin

sFigure 8

A

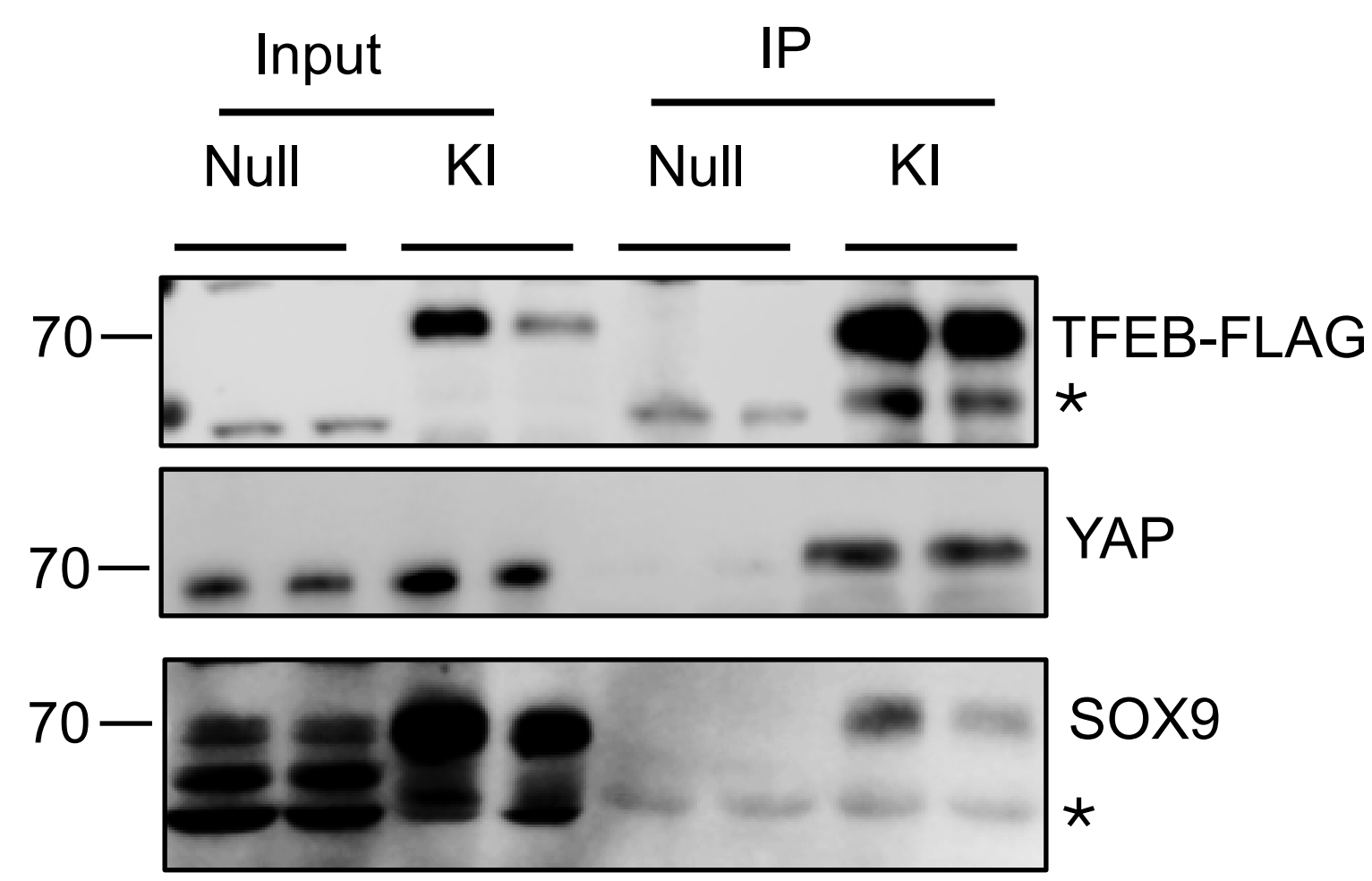
Input
IP
Null
KI
Null
KI
70—
TFEB-FLAG
*
70—
YAP
70—
SOX9
*

B

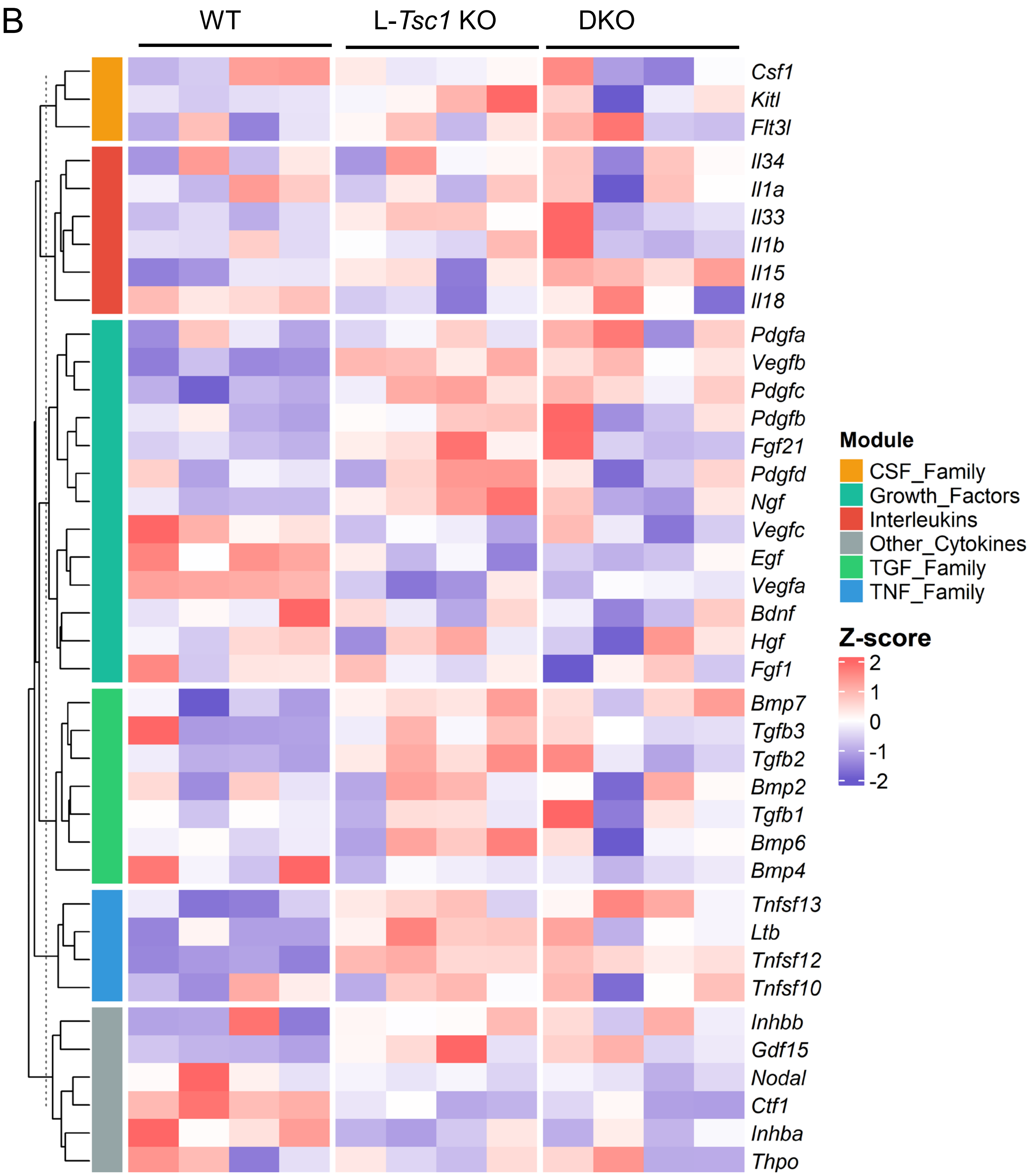
WT
L-*Tsc1* KO
DKO
Csf1
Kitl
Flt3l
Il34
Il1a
Il33
Il1b
Il15
Il18
Pdgfa
Vegfb
Pdgfc
Pdgfb
Fgf21
Pdgfd
Ngf
Vegfc
Egf
Vegfa
Bdnf
Hgf
Fgf1
Bmp7
Tgfb3
Tgfb2
Bmp2
Tgfb1
Bmp6
Bmp4
Tnfsf13
Ltb
Tnfsf12
Tnfsf10
Inhbb
Gdf15
Nodal
Ctf1
Inhba
Thpo
Module
CSF_Family
Growth_Factors
Interleukins
Other_Cytokines
TGF_Family
TNF_Family
Z-score
2
1
0
-1
-2

sFigure 9

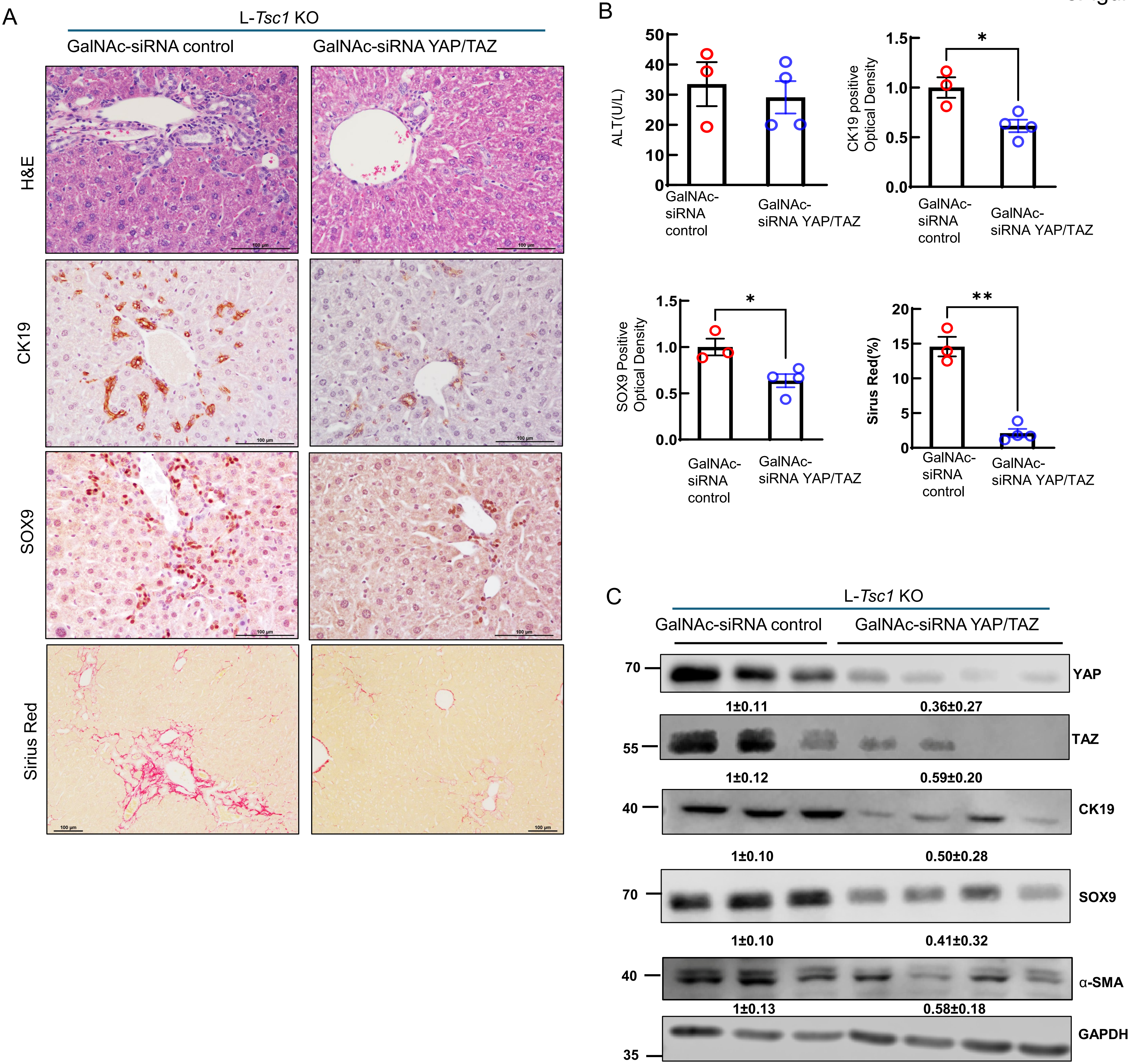
A
L-Tsc1 KO
GalNAc-siRNA control
GalNAc-siRNA YAP/TAZ
H&E
CK19
SOX9
Sirius Red
B
ALT(U/L)
CK19 positive Optical Density
SOX9 Positive Optical Density
Sirus Red(%)
GalNAc-siRNA control
GalNAc-siRNA YAP/TAZ
*
**
C
L-Tsc1 KO
GalNAc-siRNA control
GalNAc-siRNA YAP/TAZ
YAP
1±0.11
0.36±0.27
TAZ
1±0.12
0.59±0.20
CK19
1±0.10
0.50±0.28
SOX9
1±0.10
0.41±0.32
α-SMA
1±0.13
0.58±0.18
GAPDH
70
55
40
70
40
35

sFigure 10

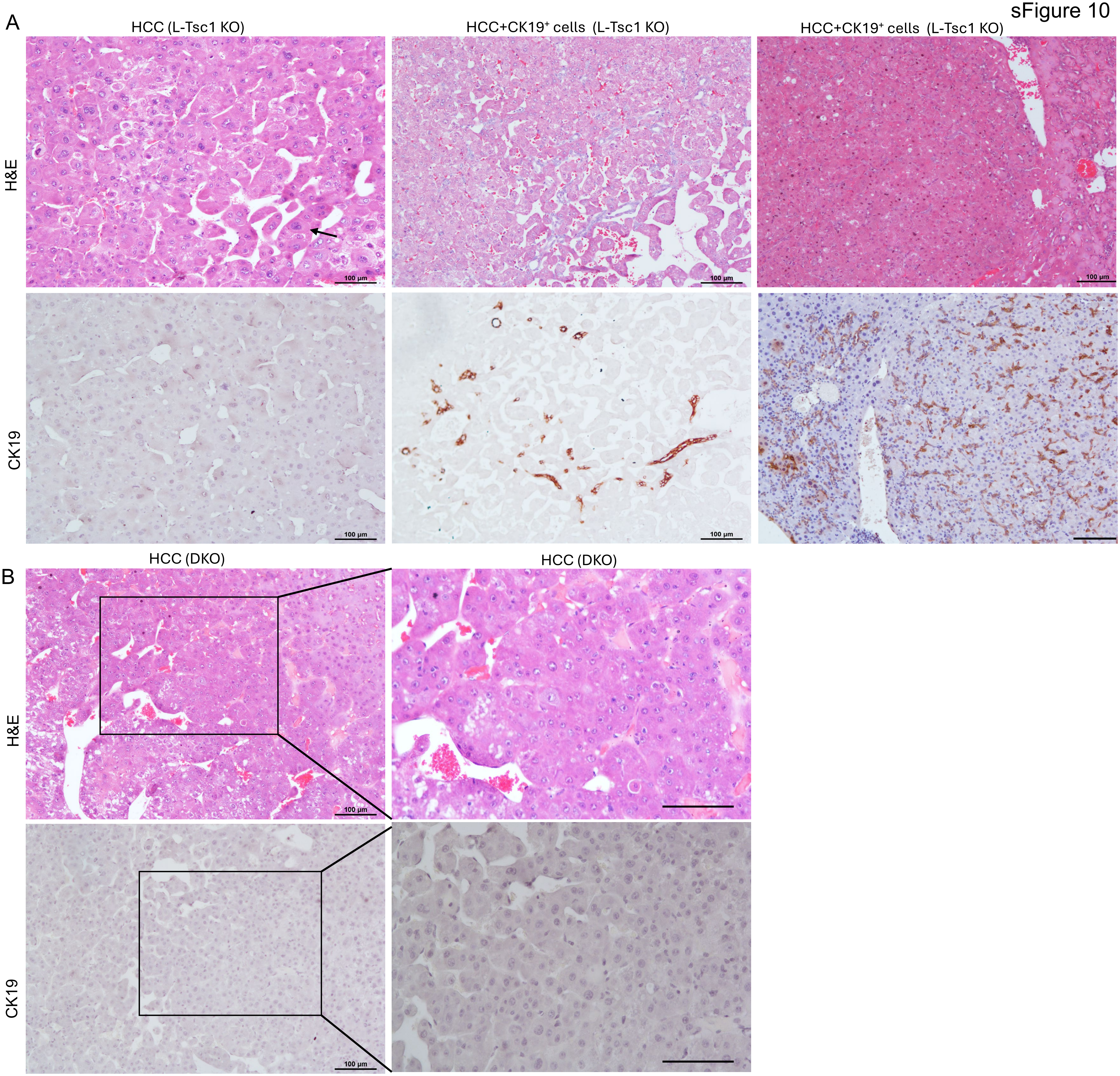
A
HCC (L-Tsc1 KO)
HCC+CK19⁺ cells (L-Tsc1 KO)
HCC+CK19⁺ cells (L-Tsc1 KO)
H&E
CK19
100 µm
B
HCC (DKO)
HCC (DKO)
H&E
CK19
100 µm

sFigure 11

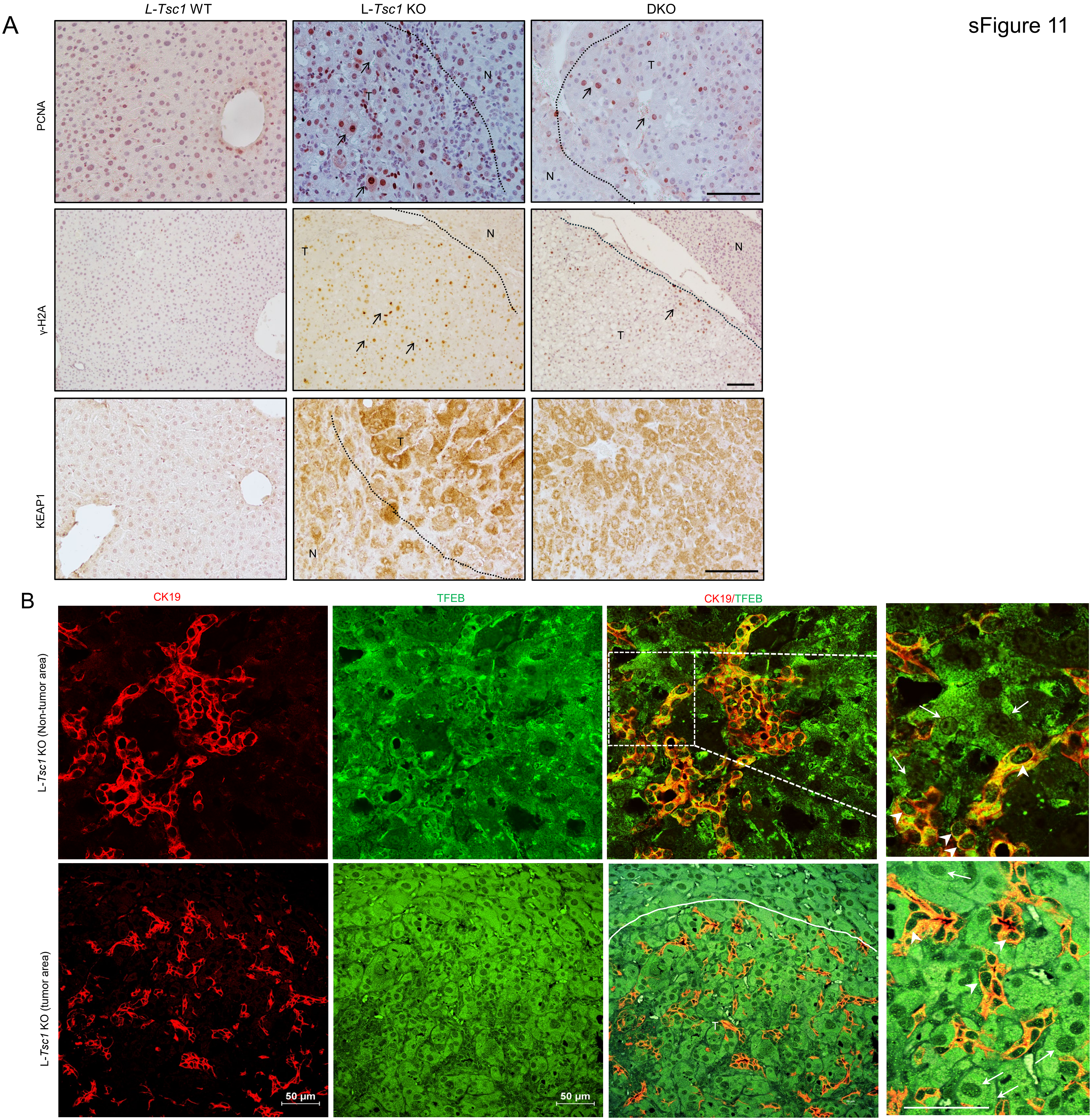
A
L-Tsc1 WT
L-Tsc1 KO
DKO
PCNA
γ-H2A
KEAP1
T
N
B
CK19
TFEB
CK19/TFEB
L-Tsc1 KO (Non-tumor area)
L-Tsc1 KO (tumor area)
50 μm
50 μm

sFigure 12

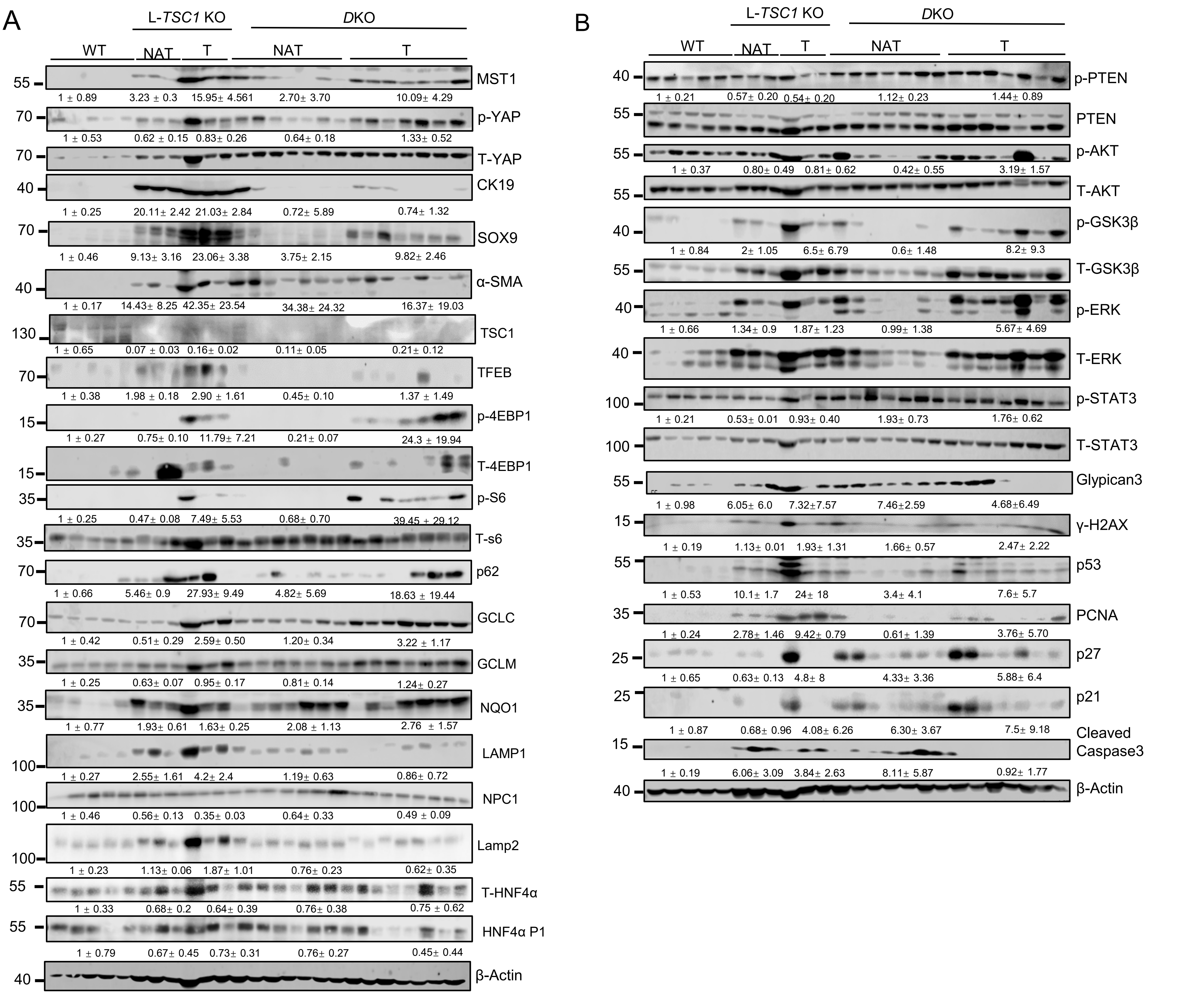

A
L-TSC1 KO
DKO
WT
NAT
T
NAT
T
MST1
p-YAP
T-YAP
CK19
SOX9
α-SMA
TSC1
TFEB
p-4EBP1
T-4EBP1
p-S6
T-s6
p62
GCLC
GCLM
NQO1
LAMP1
NPC1
Lamp2
T-HNF4α
HNF4α P1
β-Actin
B
p-PTEN
PTEN
p-AKT
T-AKT
p-GSK3β
T-GSK3β
p-ERK
T-ERK
p-STAT3
T-STAT3
Glypican3
γ-H2AX
p53
PCNA
p27
p21
Cleaved Caspase3

sFigure 13

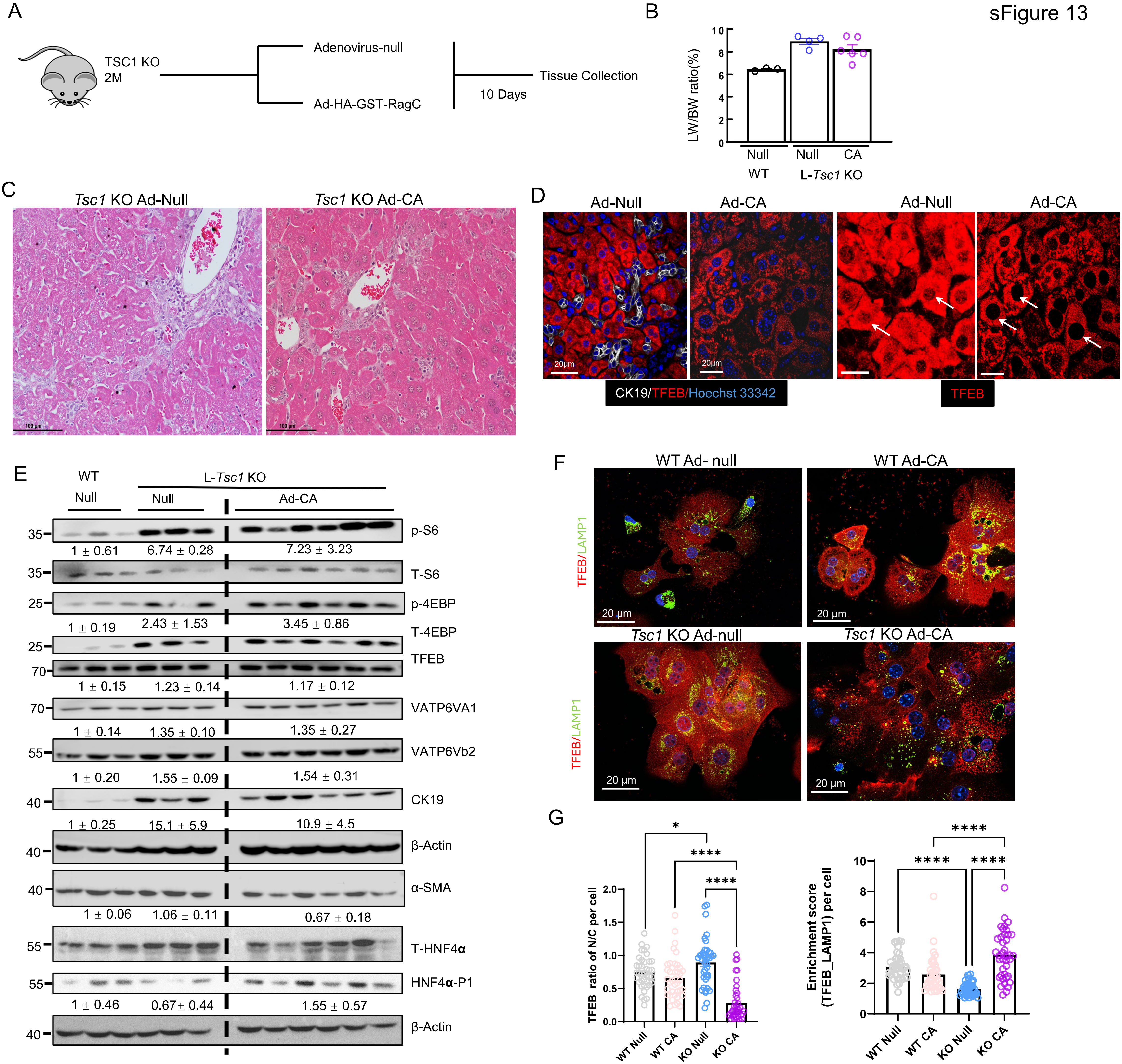
A
TSC1 KO
2M
Adenovirus-null
Ad-HA-GST-RagC
10 Days
Tissue Collection
B
LW/BW ratio(%)
Null
WT
Null
CA
L-Tsc1 KO
C
Tsc1 KO Ad-Null
Tsc1 KO Ad-CA
D
Ad-Null
Ad-CA
Ad-Null
Ad-CA
20μm
20μm
CK19/TFEB/Hoechst 33342
TFEB
E
WT
Null
L-Tsc1 KO
Null
Ad-CA
p-S6
1 ± 0.61
6.74 ± 0.28
7.23 ± 3.23
T-S6
p-4EBP
1 ± 0.19
2.43 ± 1.53
3.45 ± 0.86
T-4EBP
TFEB
1 ± 0.15
1.23 ± 0.14
1.17 ± 0.12
VATP6VA1
1 ± 0.14
1.35 ± 0.10
1.35 ± 0.27
VATP6Vb2
1 ± 0.20
1.55 ± 0.09
1.54 ± 0.31
CK19
1 ± 0.25
15.1 ± 5.9
10.9 ± 4.5
β-Actin
α-SMA
1 ± 0.06
1.06 ± 0.11
0.67 ± 0.18
T-HNF4α
HNF4α-P1
1 ± 0.46
0.67± 0.44
1.55 ± 0.57
β-Actin
F
WT Ad- null
WT Ad-CA
Tsc1 KO Ad-null
Tsc1 KO Ad-CA
TFEB/LAMP1
20 μm
G
TFEB ratio of N/C per cell
Enrichment score (TFEB_LAMP1) per cell
WT Null
WT CA
KO Null
KO CA

sFigure 14

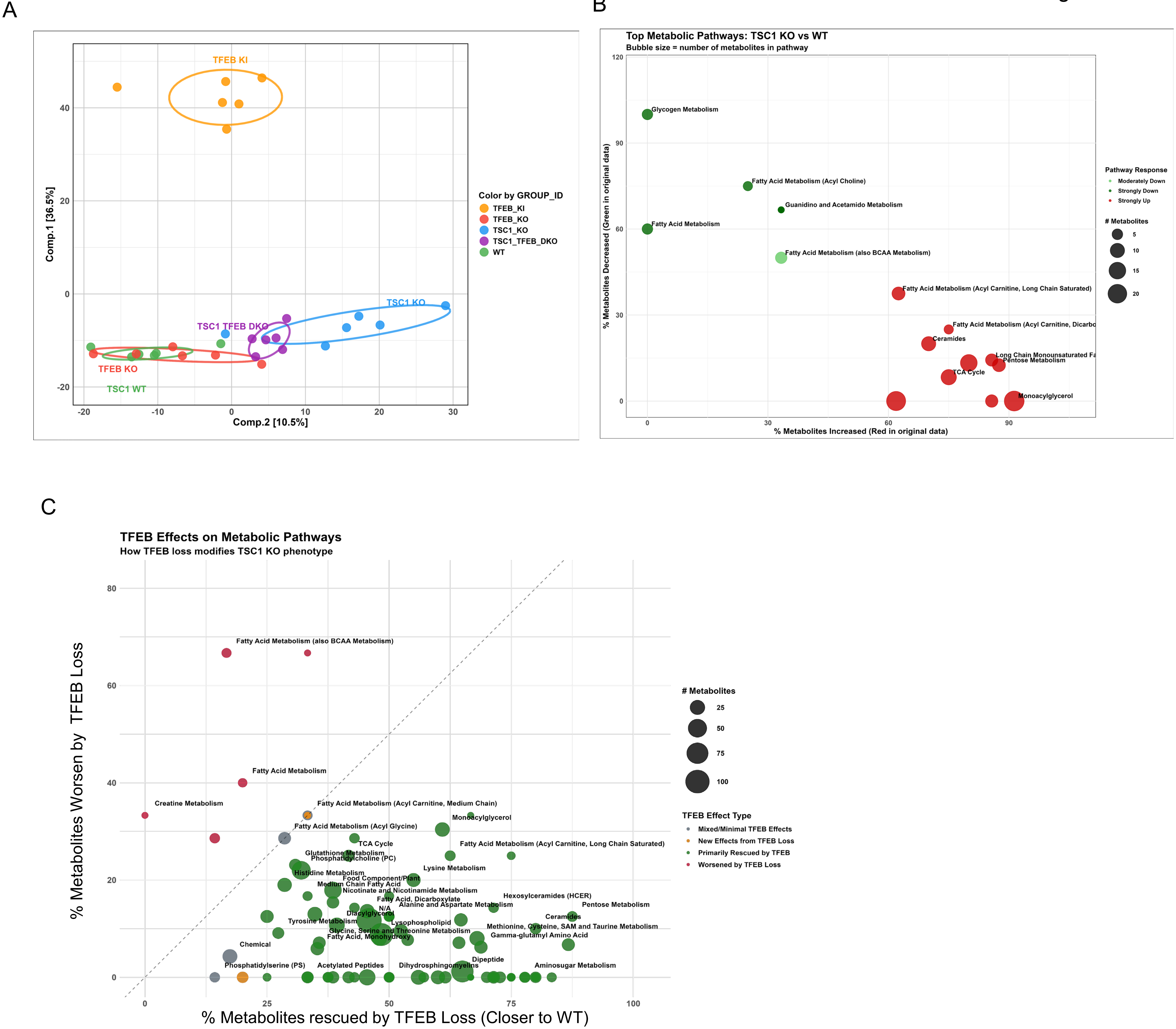

A
TFEB KI
TSC1 KO
TSC1 TFEB DKO
TFEB KO
TSC1 WT
Comp.1 [36.5%]
Comp.2 [10.5%]
Color by GROUP_ID
TFEB_KI
TFEB_KO
TSC1_KO
TSC1_TFEB_DKO
WT
B
Top Metabolic Pathways: TSC1 KO vs WT
Bubble size = number of metabolites in pathway
% Metabolites Decreased (Green in original data)
% Metabolites Increased (Red in original data)
Glycogen Metabolism
Fatty Acid Metabolism (Acyl Choline)
Guanidino and Acetamido Metabolism
Fatty Acid Metabolism
Fatty Acid Metabolism (also BCAA Metabolism)
Fatty Acid Metabolism (Acyl Carnitine, Long Chain Saturated)
Ceramides
TCA Cycle
Pentose Metabolism
Monoacylglycerol
Pathway Response
Moderately Down
Strongly Down
Strongly Up
Metabolites
C
TFEB Effects on Metabolic Pathways
How TFEB loss modifies TSC1 KO phenotype
% Metabolites Worsen by TFEB Loss
% Metabolites rescued by TFEB Loss (Closer to WT)
Fatty Acid Metabolism (also BCAA Metabolism)
Fatty Acid Metabolism
Creatine Metabolism
Fatty Acid Metabolism (Acyl Carnitine, Medium Chain)
Fatty Acid Metabolism (Acyl Glycine)
Monoacylglycerol
TCA Cycle
Fatty Acid Metabolism (Acyl Carnitine, Long Chain Saturated)
Glutathione Metabolism
Phosphatidylcholine (PC)
Lysine Metabolism
Histidine Metabolism
Food Component/Plant
Medium Chain Fatty Acid
Nicotinate and Nicotinamide Metabolism
Hexosylceramides (HCER)
Fatty Acid, Dicarboxylate
Alanine and Aspartate Metabolism
Pentose Metabolism
Diacylglycerol
Ceramides
Tyrosine Metabolism
Lysophospholipid
Methionine, Cysteine, SAM and Taurine Metabolism
Glycine, Serine and Threonine Metabolism
Gamma-glutamyl Amino Acid
Fatty Acid, Monohydroxy
Chemical
Dipeptide
Phosphatidylserine (PS)
Acetylated Peptides
Dihydrosphingomyelins
Aminosugar Metabolism
Metabolites
TFEB Effect Type
Mixed/Minimal TFEB Effects
New Effects from TFEB Loss
Primarily Rescued by TFEB
Worsened by TFEB Loss

sFigure 15

A

Red and green shaded cells indicate p≤0.05 (red indicates that the mean values are significantly higher for that comparison; green values significantly lower). Light red and light green shaded cells indicate 0.05<p<0.10 (light red indicates that the mean values trend higher for that comparison; light green values trend lower).

Gamma-glutamyl Amino Acid	L-Tsc1 KO / L-Tsc1 WT	DKO / L-Tsc1 WT	L-Tfeb KO / L-Tfeb WT	TFEB KI / TFEB WT	DKO / L-Tsc1 KO	DKO / L-Tfeb KO
gamma-glutamylalanine	1.24	0.96	1	0.81	0.78	0.97
gamma-glutamylcysteine	0.44	0.31	1.33	2.18	0.7	0.23
gamma-glutamylglutamate	1.11	0.84	0.93	0.88	0.76	0.91
gamma-glutamylglutamine	1.55	1.38	0.92	5.87	0.89	1.5
gamma-glutamylglycine	0.76	0.89	0.57	0.46	1.17	1.57
gamma-glutamylhistidine	1.52	1.23	0.91	5.93	0.81	1.35
gamma-glutamylisoleucine*	1.89	1.45	1.04	2.13	0.77	1.4
gamma-glutamylleucine	1.65	1.36	1.01	2.42	0.83	1.35
gamma-glutamyl-epsilon-lysine	1.79	1.29	1.02	16.78	0.72	1.27
gamma-glutamylphenylalanine	1.23	1.14	1.04	3.7	0.92	1.09
gamma-glutamylthreonine	0.81	0.83	0.74	1.03	1.03	1.12
gamma-glutamyltryptophan	1.13	0.85	1.07	6.81	0.75	0.8
gamma-glutamyltyrosine	0.51	0.59	0.78	4.81	1.16	0.76
gamma-glutamylvaline	1.49	1.24	0.85	0.75	0.83	1.46
gamma-glutamylserine	0.65	0.71	0.5	2.78	1.1	1.42
gamma-glutamyl-2-aminobutyrate	1.12	0.33	0.27	0.56	0.29	1.22

B

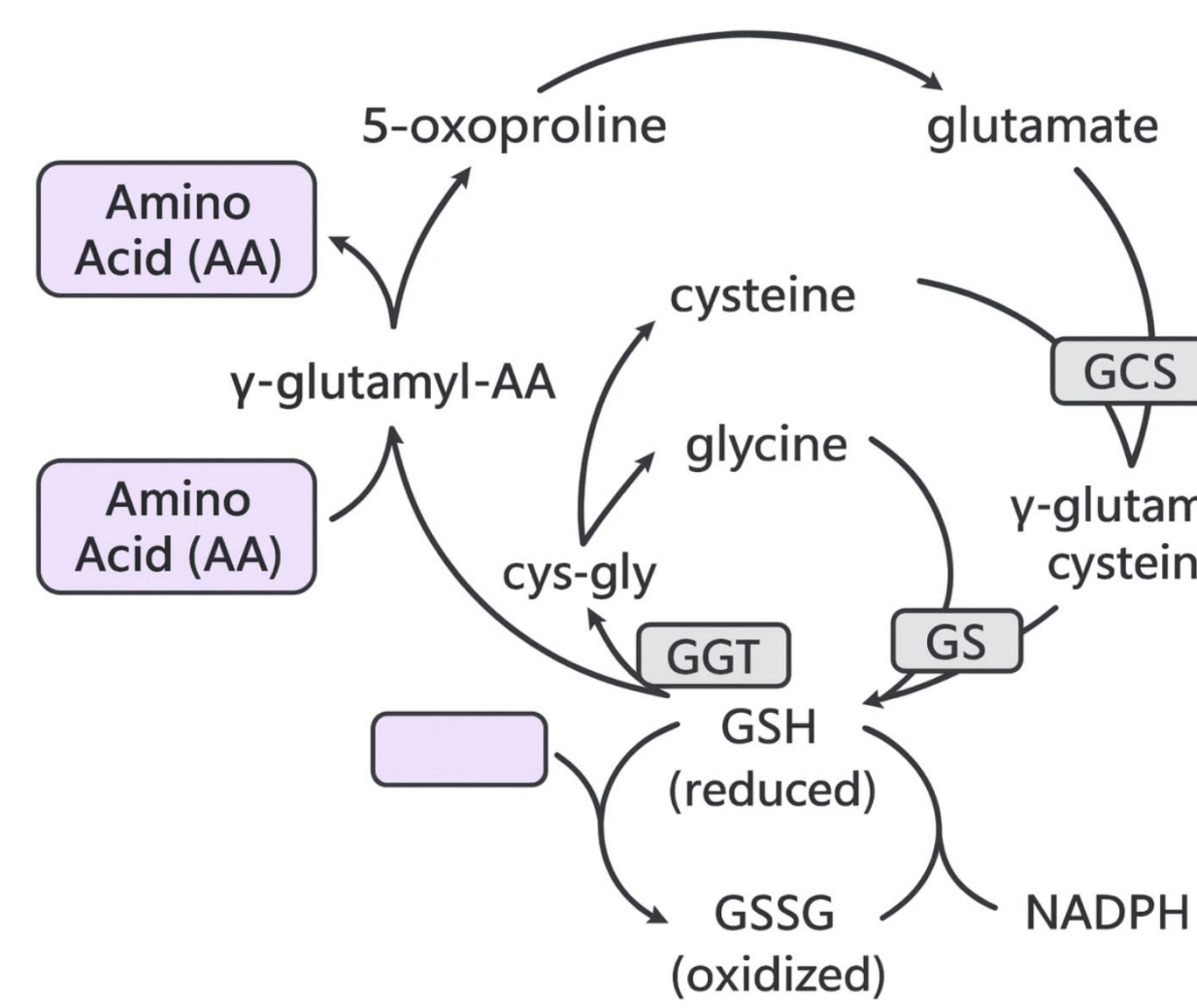

C

Ceramides	L-Tsc1 KO / L-Tsc1 WT	DKO / L-Tsc1 WT	L-Tfeb KO / L-Tfeb WT	TFEB KI / TFEB WT	DKO / L-Tsc1 KO	DKO / L-Tfeb KO
N-palmitoyl-sphingosine (d18:1/16:0)	1.9	1.82	1.5	8.61	0.96	1.21
N-stearoyl-sphingosine (d18:1/18:0)*	3.06	2.41	2.31	2.56	0.79	1.04
N-palmitoyl-sphingadienine (d18:2/16:0)*	2.09	1.92	1.34	8.67	0.92	1.43
N-behenoyl-sphingadienine (d18:2/22:0)*	0.58	0.66	0.78	0.78	1.14	0.84
N-palmitoyl-heptadecasphingosine (d17:1/16:0)*	1.66	1.64	1.41	1.94	0.98	1.16
ceramide (d18:1/14:0, d16:1/16:0)*	2.38	2.06	1.7	2.27	0.87	1.22
ceramide (d18:1/17:0, d17:1/18:0)*	3.3	2.53	2.51	5.84	0.77	1.01
ceramide (d18:1/20:0, d16:1/22:0, d20:1/18:0)*	1.08	1.16	1.14	1.03	1.08	1.02
ceramide (d16:1/24:1, d18:1/22:1)*	0.75	0.79	0.57	0.4	1.06	1.39
ceramide (d18:2/24:1, d18:1/24:2)*	1.37	1.64	1.23	4.57	1.19	1.33

D

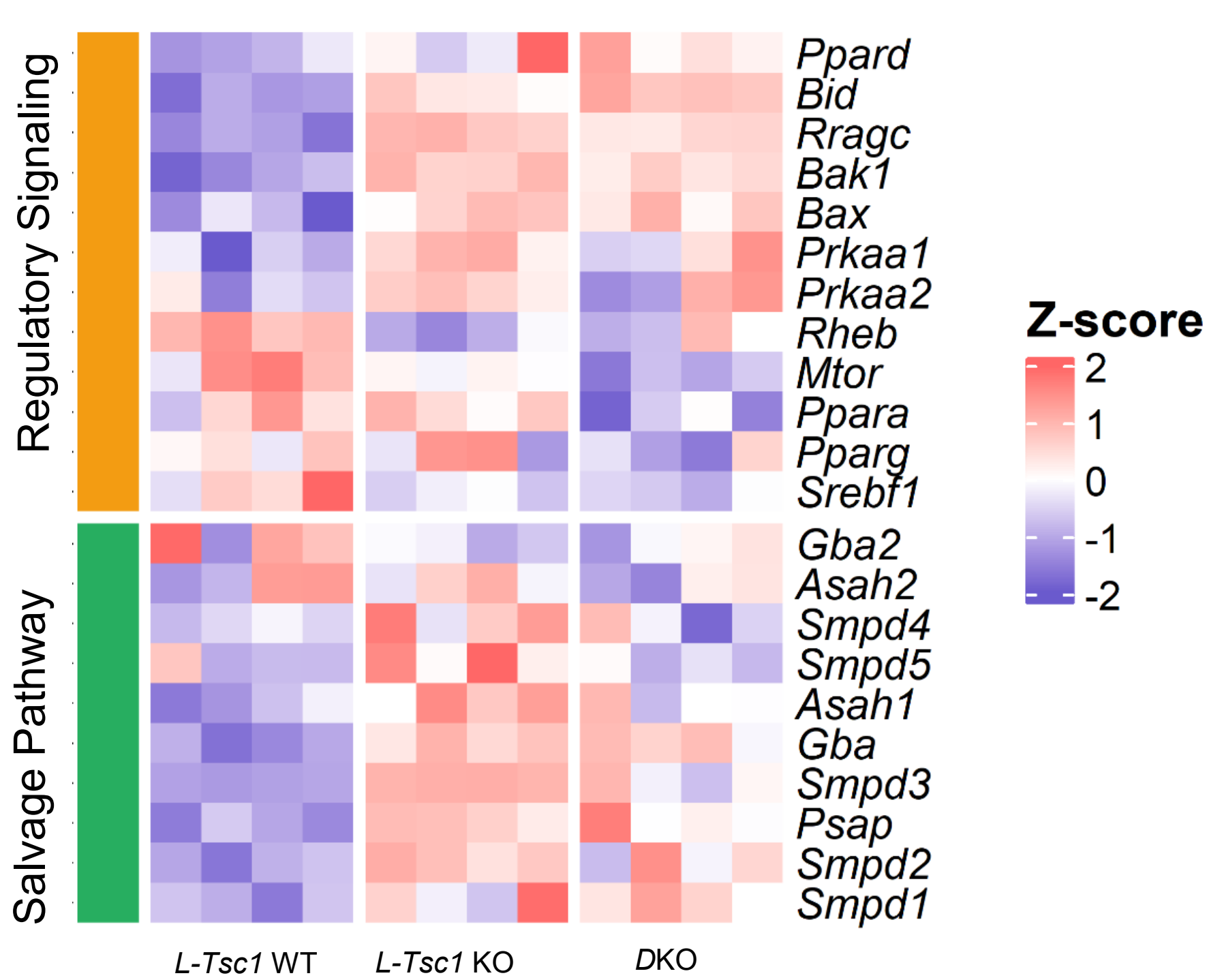

sFigure 16

A

Red and green shaded cells indicate p≤0.05 (red indicates that the mean values are significantly higher for that comparison; green values significantly lower). Light red and light green shaded cells indicate 0.05<p<0.10 (light red indicates that the mean values trend higher for that comparison; light green values trend lower).

Polyamine Metabolism	L-Tsc1 KO / L-Tsc1 WT	DKO / L-Tsc1 WT	L-Tfeb KO / L-Tfeb WT	TFEB KI / TFEB WT	DKO / L-Tsc1 KO	DKO / L-Tfeb KO
putrescine	2.14	1.34	1.08	14.18	0.63	1.24
N-acetylputrescine	1.09	0.87	1.1	10.85	0.8	0.79
N-acetyl-isoputreanine	1.96	1.59	0.99	2.98	0.81	1.61
spermidine	1	1.01	0.93	2.48	1.01	1.09
(N(1) + N(8))-acetylspermidine	2.02	1.13	0.77	6.58	0.56	1.47
5-methylthioadenosine (MTA)	1.05	0.98	1.06	1.26	0.93	0.93
guanidine	1.05	1	1	1	0.96	1

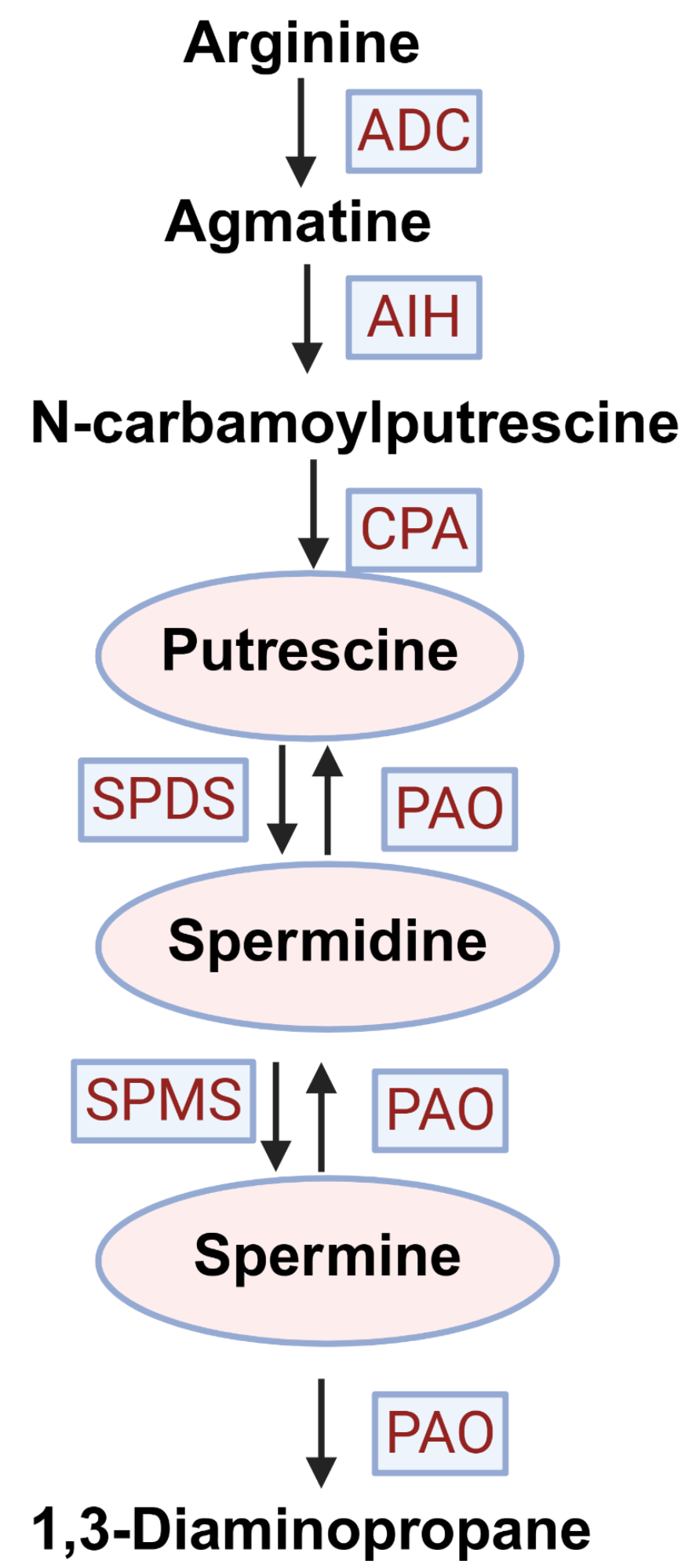

B

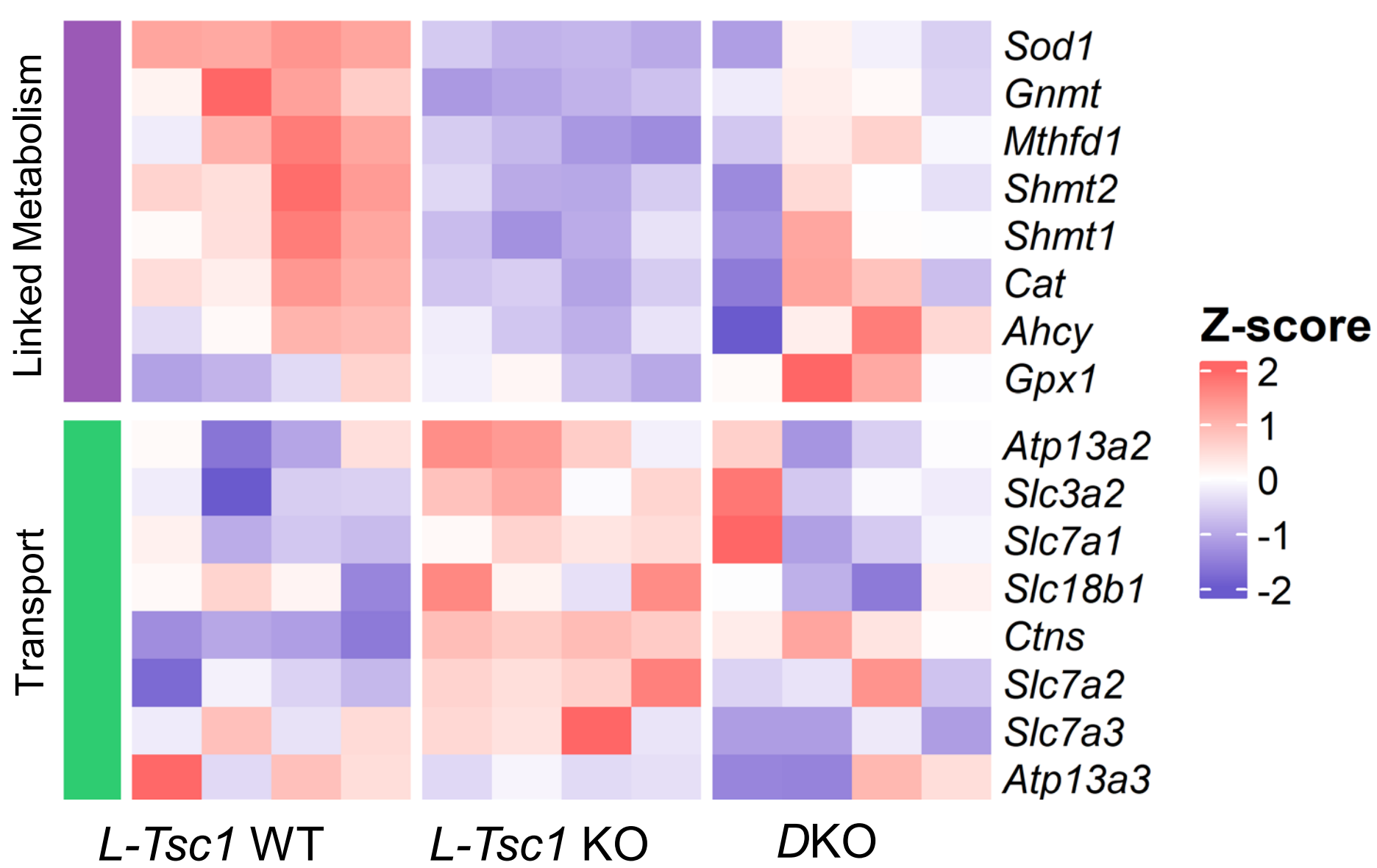

sFigure 17

A

Red and green shaded cells indicate p≤0.05 (red indicates that the mean values are significantly higher for that comparison; green values significantly lower). Light red and light green shaded cells indicate 0.05<p<0.10 (light red indicates that the mean values trend higher for that comparison; light green values trend lower).

Bis(monoacylglycero)phosphate (BMP)	L-Tsc1 KO / L-Tsc1 WT	DKO / L-Tsc1 WT	L-Tfeb KO / L-Tfeb WT	TFEB KI / TFEB WT	DKO / L-Tsc1 KO	DKO / L-Tfeb KO
BMP(18:1/18:1) [1]	2.32	1.54	0.78	5.01	0.66	1.98
BMP(18:1/18:1) [2]	1.67	0.8	0.6	4.3	0.48	1.33

B

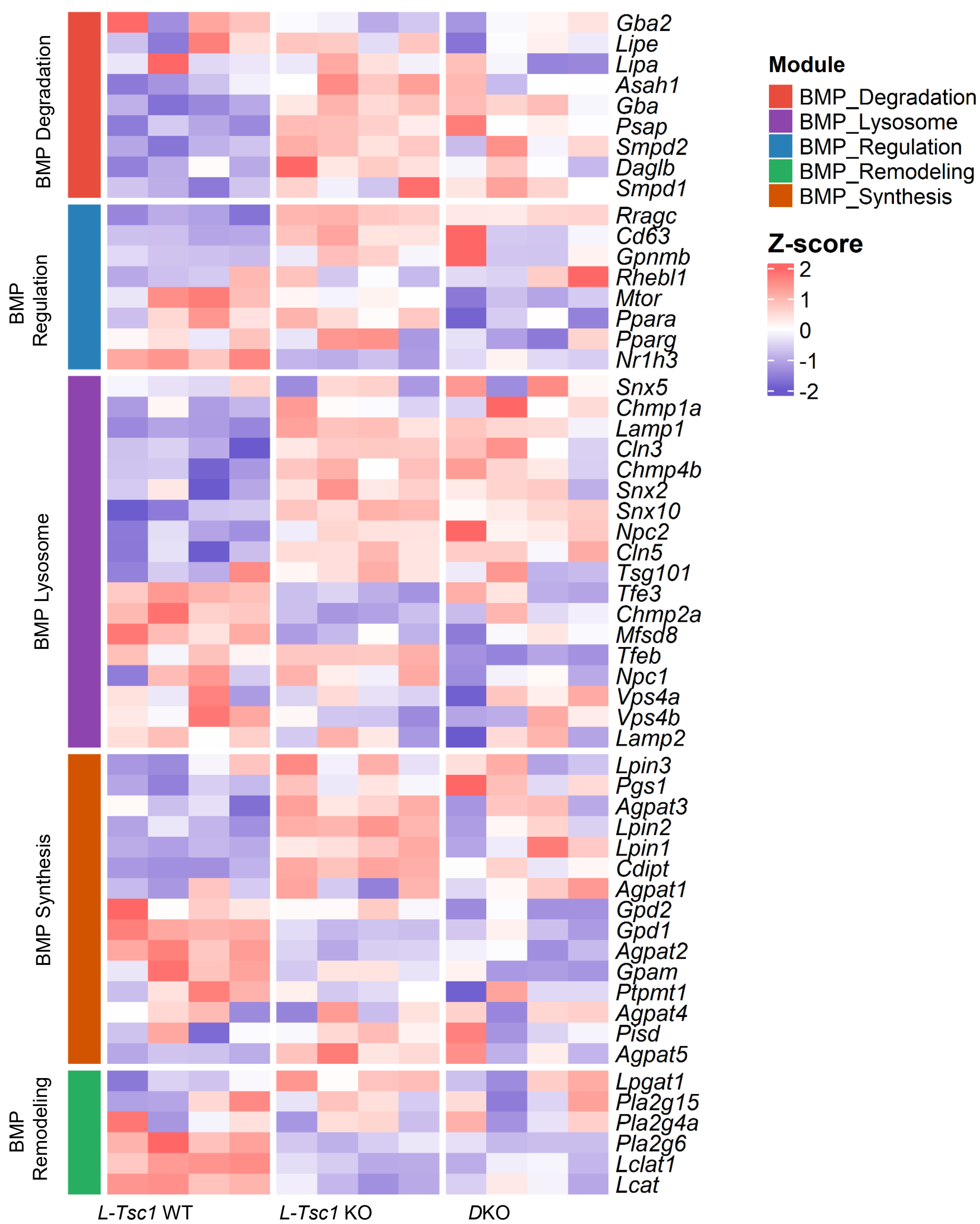

sFigure 18

A

Red and green shaded cells indicate p≤0.05 (red indicates that the mean values are significantly higher for that comparison; green values significantly lower). Light red and light green shaded cells indicate 0.05<p<0.10 (light red indicates that the mean values trend higher for that comparison; light green values trend lower).

TCA Cycle	L-Tsc1 KO / L-Tsc1 WT	DKO / L-Tsc1 WT	L-Tfeb KO / L-Tfeb WT	TFEB KI / TFEB WT	DKO / L-Tsc1 KO	DKO / L-Tfeb KO
citrate	1.2	1.48	1.28	6.05	1.24	1.15
aconitate [cis or trans]	1.51	1.76	1	5.04	1.17	1.75
isocitrate	0.95	1.04	1.08	1.12	1.1	0.96
alpha-ketoglutarate	0.85	0.58	0.68	3.84	0.68	0.85
succinylcarnitine (C4-DC)	0.82	0.73	0.75	2.1	0.88	0.97
succinate	1.25	1.31	0.9	1.17	1.05	1.46
fumarate	2.51	2.19	1.02	0.35	0.87	2.15
malate	1.24	1.31	1.11	1.11	1.06	1.18
itaconate	3.79	2.19	1.69	6.15	0.58	1.29
tricarballylate	3.52	3.17	1.31	1.7	0.9	2.43
2-methylcitrate/homocitrate	1.75	1.27	0.96	0.44	0.73	1.33
mesaconate (methylfumarate)	2.73	1.07	1.07	5.6	0.39	1.01

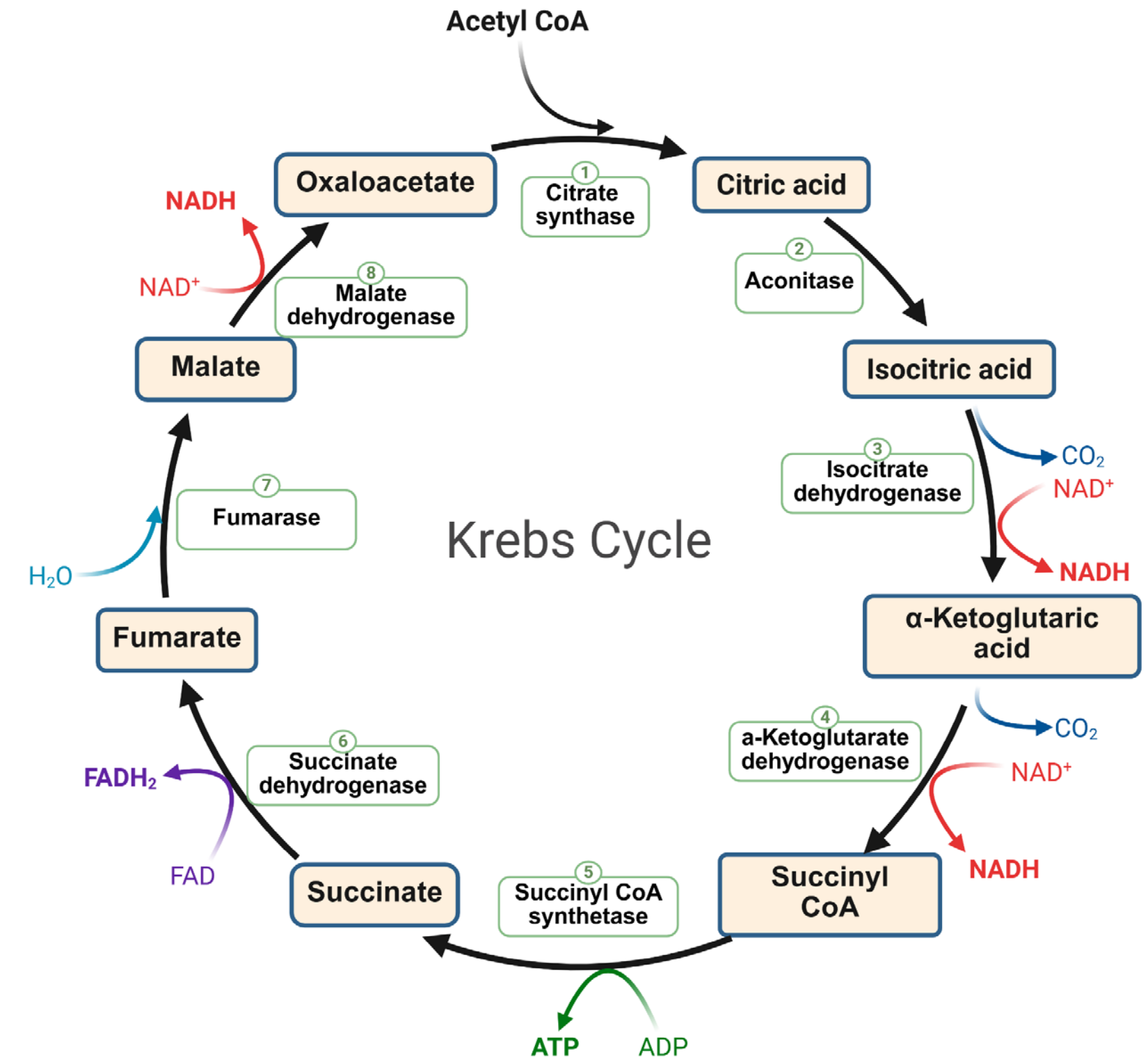

B

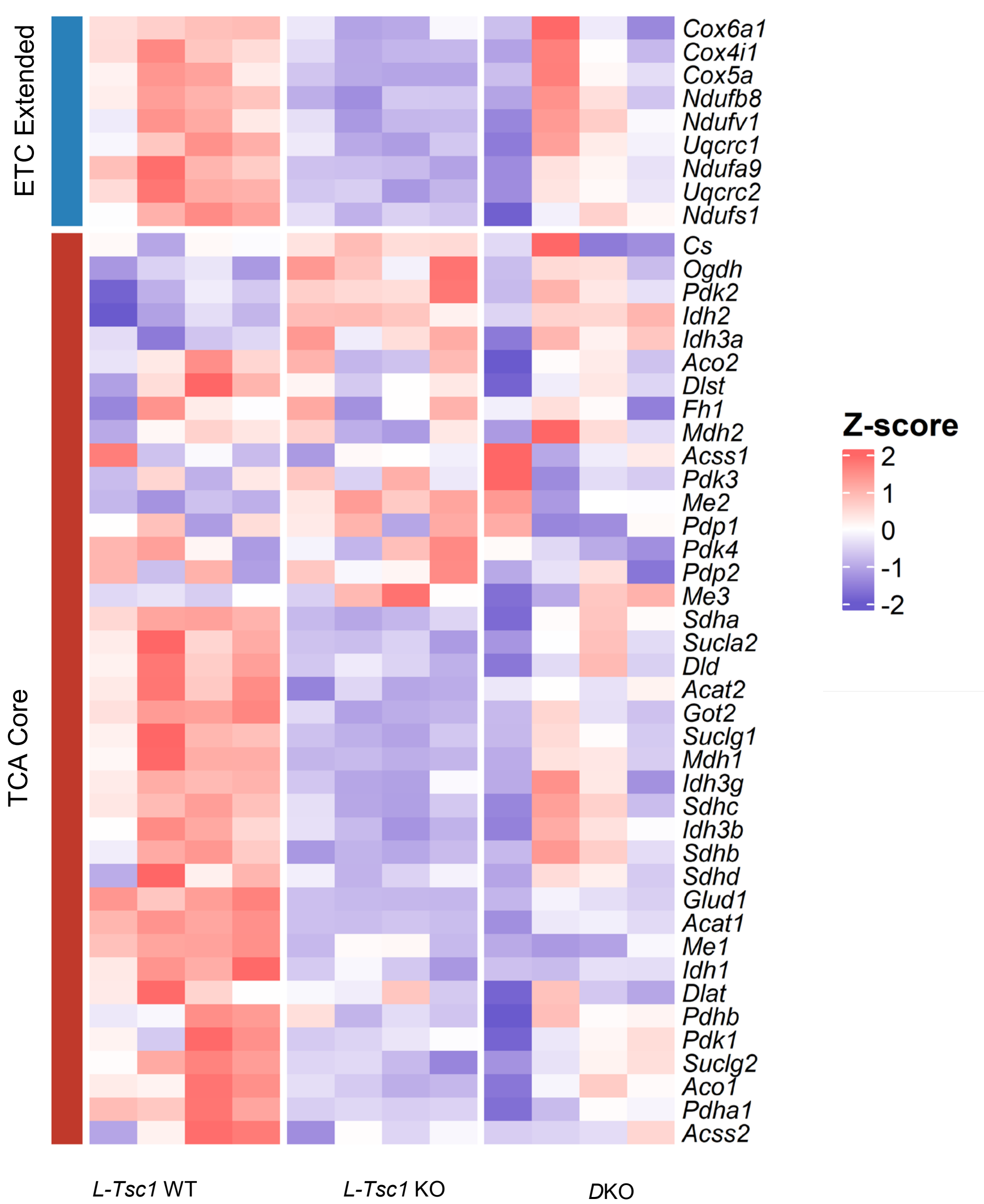

Red and green shaded cells indicate p≤0.05 (red indicates that the mean values are significantly higher for that comparison; green values significantly lower). Light red and light green shaded cells indicate 0.05<p<0.10 (light red indicates that the mean values trend higher for that comparison; light green values trend lower).

Bile Acid	L-Tsc1 KO / L-Tsc1 WT	DKO / L-Tsc1 WT	L-Tfeb KO / L-Tfeb WT	TFEB KI / TFEB WT	DKO / L-Tsc1 KO	DKO / L-Tfeb KO
cholate	2	1	3.89	6.64	0.5	0.4
glycocholate	4.38	1.52	1.73	1.54	0.35	0.52
taurocholate	1.64	1.27	1.59	1.51	0.77	0.77
chenodeoxycholate	1.6	1.29	1.52	0.97	0.81	0.86
glycochenodeoxycholate	2.29	1.16	1.55	0.86	0.51	0.69
taurochenodeoxycholate	1.51	1.17	2.19	1.69	0.78	0.76
beta-muricholate	1.04	1.09	0.51	0.14	1.05	1.7
alpha-muricholate	1.17	1.03	0.82	1.21	0.89	0.92
tauro-beta-muricholate	1.08	0.84	2.12	2.48	0.78	0.56
glyco-beta-muricholate**	1.48	0.84	1.81	1.55	0.57	0.42
cholate sulfate	0.46	0.47	3.48	2.69	1.01	0.5
deoxycholate	2.43	1.4	1.26	3.1	0.58	0.64
glycodeoxycholate	4.55	1.57	1.74	0.35	0.35	0.73
taurodeoxycholate	2.38	1.36	1.44	0.54	0.57	0.87
6beta-hydroxylithocholate	2.96	1.75	0.48	0.35	0.59	1.74
taurolithocholate	2.63	1.51	2.46	3.19	0.57	1
ursodeoxycholate	1.4	1.28	1.02	0.34	0.91	1.21
glycoursodeoxycholate	1.78	0.92	1.59	0.49	0.52	0.5
tauroursodeoxycholate	1.26	0.98	2.68	0.53	0.78	0.61
taurohyocholate*	1.55	1.34	3.68	2.09	0.87	0.47
hyodeoxycholate	3.77	1.93	0.29	0.39	0.51	1.52
taurohyodeoxycholic acid	1.92	1.01	1.45	0.55	0.53	0.61
taurocholenate sulfate*	4.61	1	4.97	4.96	0.22	0.2
7-ketodeoxycholate	1.07	0.65	0.9	1.58	0.61	0.57
taurochenodeoxycholic acid (7 or 27)-sulfate	0.56	0.61	2.33	1.05	1.1	0.65
ursocholate	0.66	0.6	0.86	0.32	0.91	0.62

sFigure 20

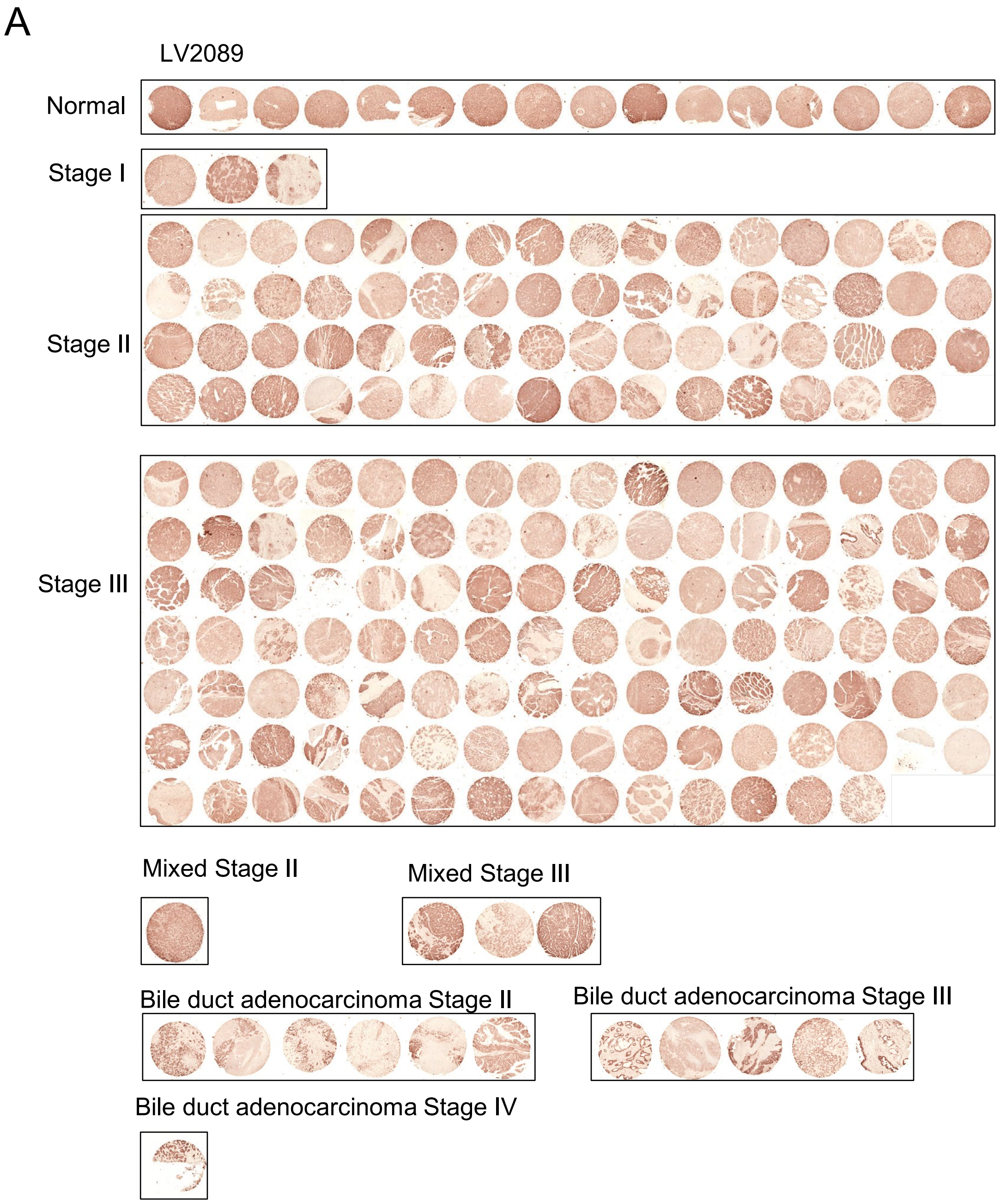

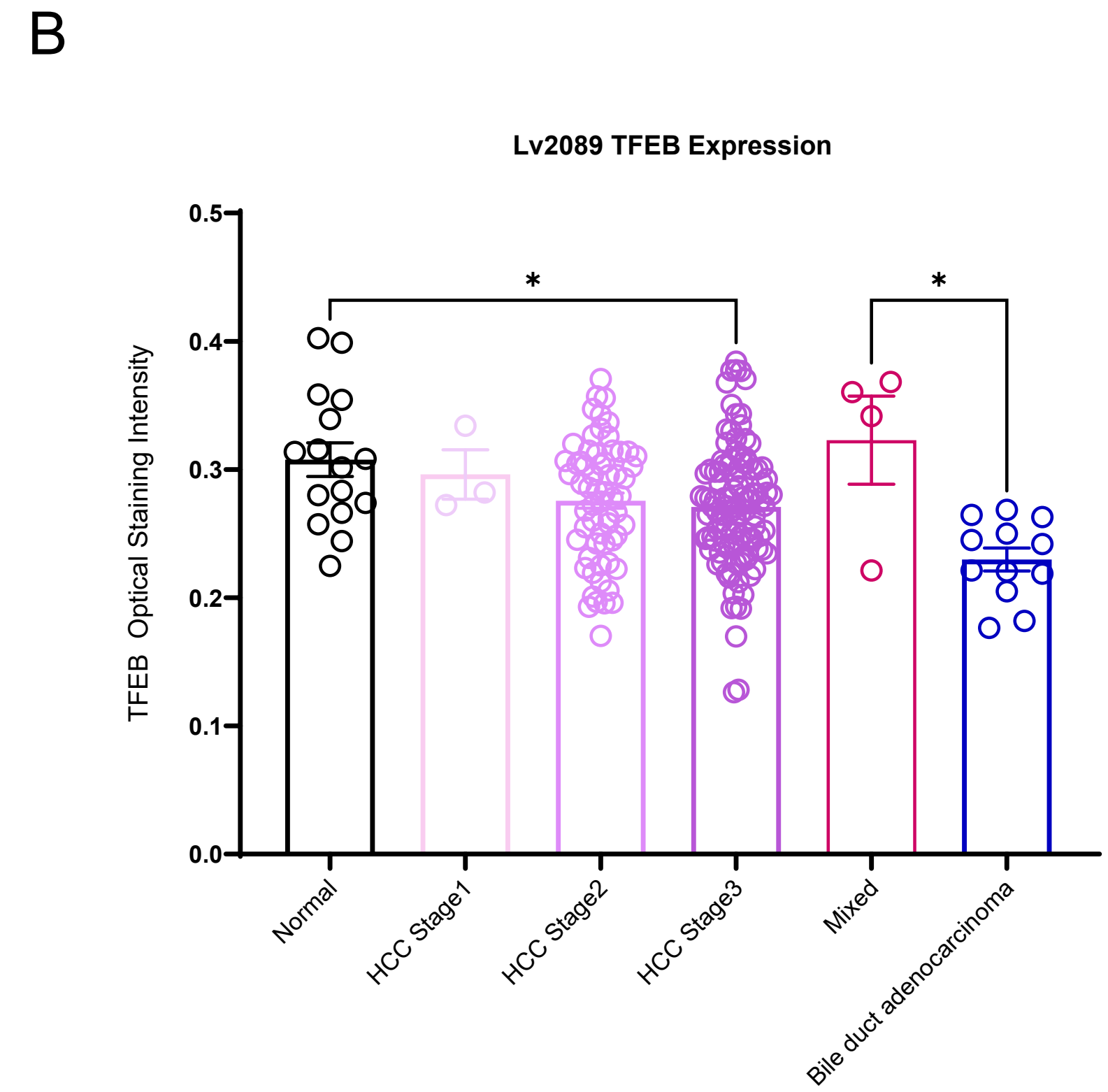

C

%(n/N)	$TFEB^{hi}$, $p\text{-}S6^{hi}$	$TFEB^{hi}$, $p\text{-}S6^{lo}$	$TFEB^{lo}$, $p\text{-}S6^{hi}$	$TFEB^{lo}$, $p\text{-}S6^{lo}$
Normal	56.25(9/16)	37.5(6/16)	0(0/16)	6.25(1/16)
Malignant Tumor stage I	33.33 (1/3)	0(0/3)	66.67(2/3)	0
Malignant Tumor stage II	24.24(16/66)	36.36(24/66)	37.88(25/66)	1.52(1/66)
Malignant Tumor stage III	58.77(67/114)	24.56(28/114)	7.89(9/114)	8.77(10/114)

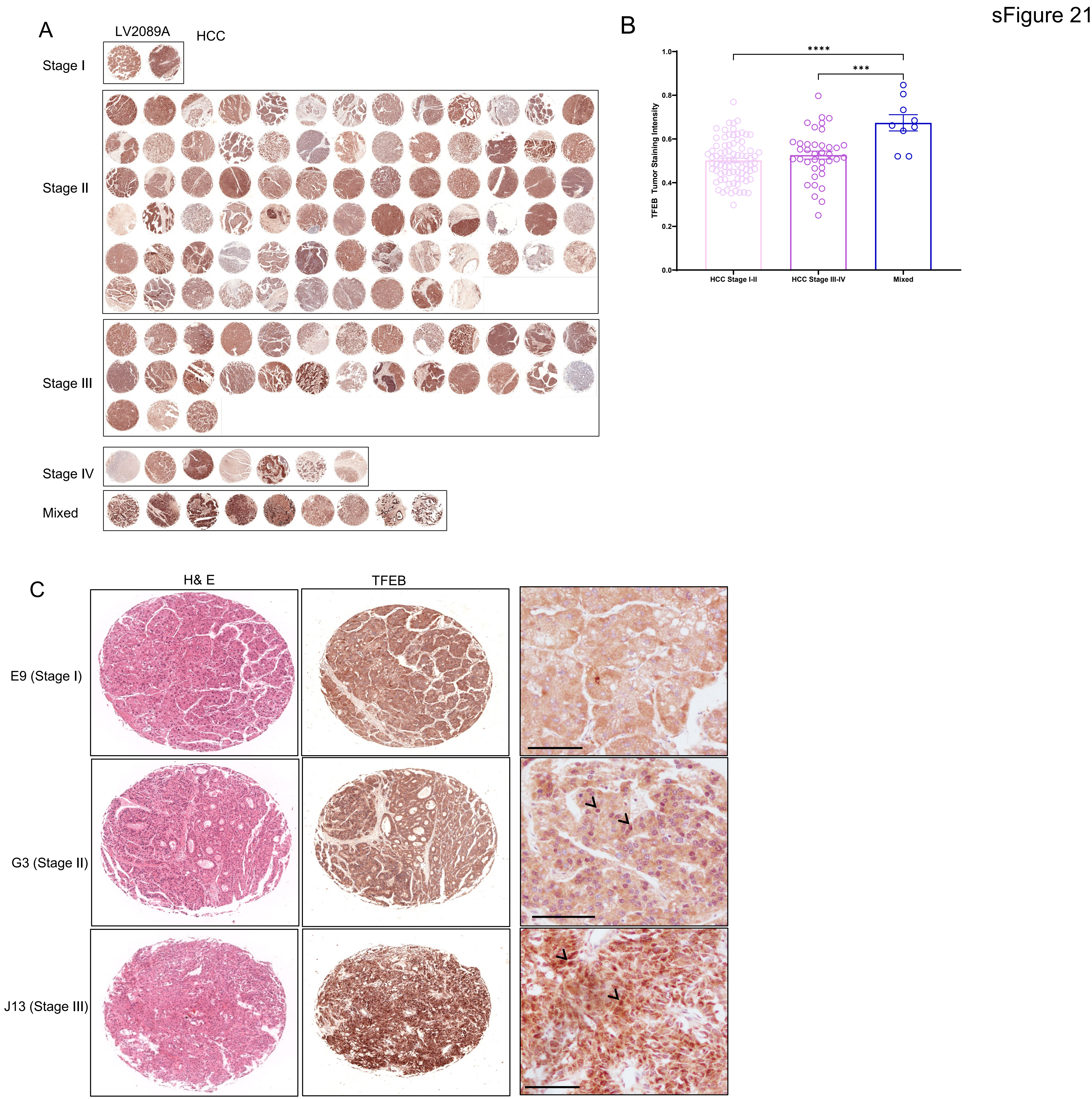

sFigure 21
A
LV2089A
HCC
Stage I
Stage II
Stage III
Stage IV
Mixed
B
TFEB Tumor Staining Intensity
1.0
0.8
0.6
0.4
0.2
0.0
****
***
HCC Stage I-II
HCC Stage III-IV
Mixed
C
H& E
TFEB
E9 (Stage I)
G3 (Stage II)
J13 (Stage III)

sFigure 22

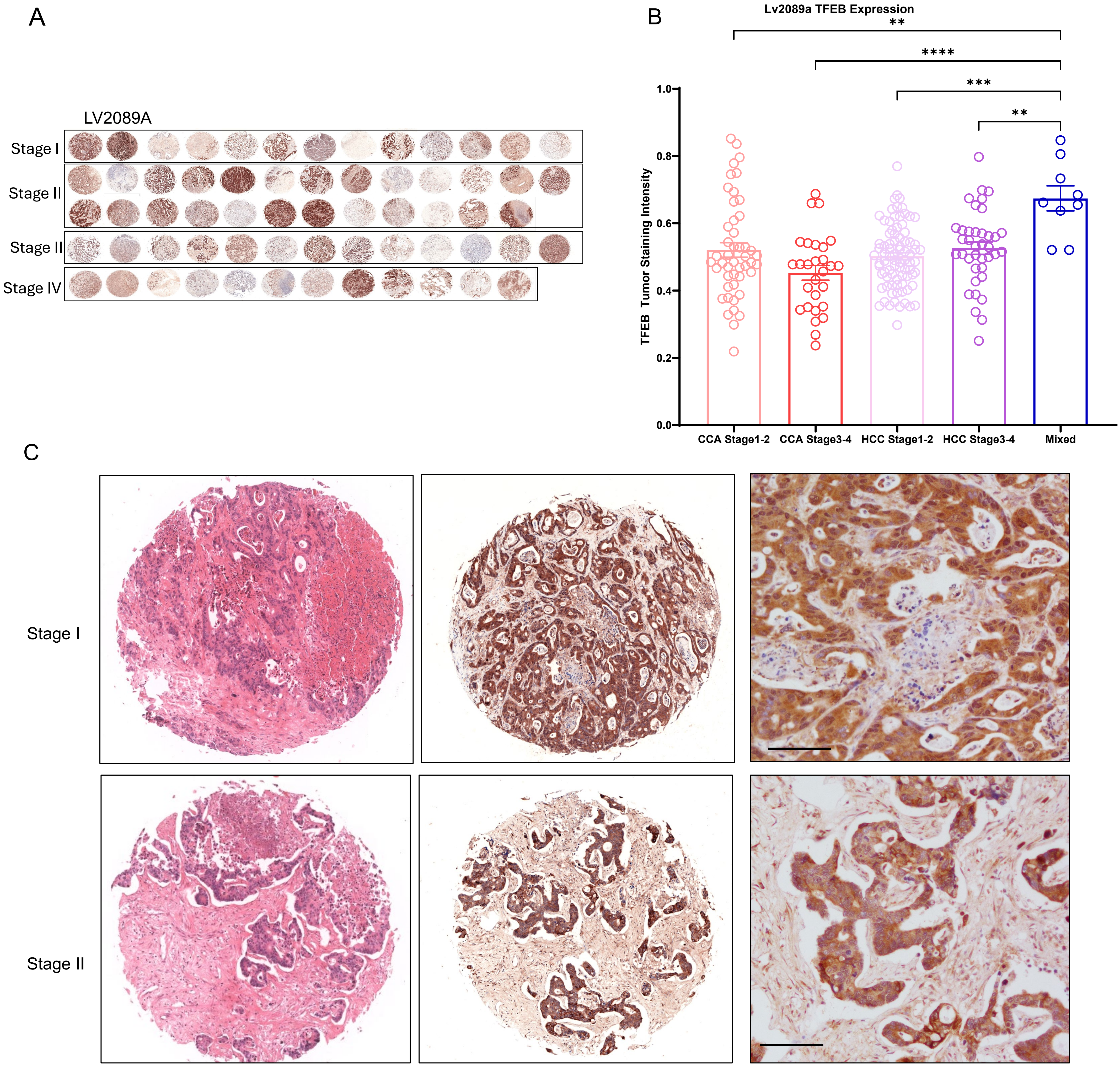

A
LV2089A
Stage I
Stage II
Stage II
Stage IV
B
Lv2089a TFEB Expression
**
****
***
**
TFEB Tumor Staining Intensity
1.0
0.8
0.6
0.4
0.2
0.0
CCA Stage1-2
CCA Stage3-4
HCC Stage1-2
HCC Stage3-4
Mixed
C
Stage I
Stage II

Table 1. Antibody information

Name	Supplier	Cat no.	Clone no.
4EBP1	Cell Signaling Technology	9452	
p-4EBP1 (Ser65)	Cell Signaling Technology	9451	
β-Actin	Sigma-Aldrich	A5441	AC-15
AKT (pan)	Cell Signaling Technology	4691	C67E7
p-AKT (Ser473)	Cell Signaling Technology	4058	193H12
p-AKT substrate (RXXS*/T*)	Cell Signaling Technology	9614	110B7
Atg5-Atg12	Cell Signaling Technology	12994	D5F5U
CK19	DSHB	AB_2133570	
Cleaved Caspase-3	Cell Signaling Technology	9661	
CYP2E1	Abcam	ab28146	
eIF2α	Cell Signaling Technology	9722	
p-eIF2α (Ser51)	Cell Signaling Technology	9721	
Epcam	DSHB	G8.8	
ERK	Cell Signaling Technology	9102	
p-ERK (Thr202/Tyr204)	Cell Signaling Technology	9101	
F4/80	Invitrogen	14-4801	BM8
FLAG	Sigma-Aldrich	F3165	M2 (clone)
FLCN (Folliculin)	Abcam	ab124885	
GAPDH	Cell Signaling Technology	2118	14C10
GCLC	Gift from Terry Kavanagh's lab at University of Washington, Seattle, Washington, USA		
GCLM	Gift from Terry Kavanagh's lab at University of Washington, Seattle, Washington, USA		
Glutamine synthase	Abcam	ab176562	
Glypican 3	Abcam	ab66596	
GSK-3α/β	Cell Signaling Technology	5676	D75D3
p-GSK3β (Ser9)	Cell Signaling Technology	5558	D85E12
p-γ-H2A.X (Ser139)	Cell Signaling Technology	9718	20E3
HepPar-1 (Hepatocyte)	Bio-Techne	NBP2-45272	
HNF4α (P1)	R&D Systems	PP-K9218-00	K9218
HNF4α (P2)	R&D Systems	PP-H6939-00	H6930
HNF4α (pan/total)	R&D Systems	PP-H1415-00	
KEAP1	Proteintech	10503-2-AP	
LAMP1	DSHB	1D4B	
LAMP2	DSHB	ABL-93	
LC3B	Custom-generated (Ding lab)	Custom rabbit polyclonal raised against the N-terminal 14 aa of human LC3B plus cysteine (PSEKTFKQRRTFEQC); generated in-house (Wen-Xing Ding lab)	
MST-1	Cell Signaling Technology	3682	
mTOR	Cell Signaling Technology	2983	7C10
NPC1	Abcam	ab134113	EPR5209
NQO1	Abcam	ab28947	A180
p21	Santa Cruz Biotechnology	sc-6246	F-5
p27	Cell Signaling Technology	3698	SX53G8.5
p53	Cell Signaling Technology	32532	
p62	Abnova	H00008878-M01	2C11
PCNA	Santa Cruz Biotechnology	sc-56	
PGC1α	Abnova	PAB12061	
PTEN	Cell Signaling Technology	9552	
p-PTEN (Ser380/Thr382/383)	Cell Signaling Technology	9554	
RagA	Cell Signaling Technology	4357	D8B5
RagC	Cell Signaling Technology	9480	
S6	Cell Signaling Technology	2217	5G10
p-S6 (Ser235/236)	Cell Signaling Technology	4858	D57.2.2E
α-SMA	Abcam	ab5694	
SOX9	MilliporeSigma	AB5535	
STAT3	Cell Signaling Technology	4904	79D7
p-STAT3 (Ser727)	Cell Signaling Technology	9134	
TAZ	Cell Signaling Technology	83669	E8E9G
TFE3	Sigma-Aldrich	HPA023881	
TFEB	Bethyl Laboratories	A303-673	

TSC1	Cell Signaling Technology	6935	D43E2
V-ATPase V1A (ATP6V1A)	Proteintech	17115-1-AP	
V-ATPase V1B2 (ATP6V1B2)	Proteintech	15097-1-AP	
YAP	Cell Signaling Technology	14074	D8H1X
p-YAP (Ser127)	Cell Signaling Technology	4911	
