# Supplementary material for: Noncanonical mTORC1-TFEB activation promotes hepatocyte plasticity and high-grade malignancy in hepatocellular carcinoma
Source: J Clin Invest. 2026 Aug 6;136(19):e206334. doi: 10.1172/JCI206334 (PMC13626827; doi:10.1172/JCI206334)
Supplement: Supplementary file 2 [file jci-136-206334-s117.pdf]

Figure 1

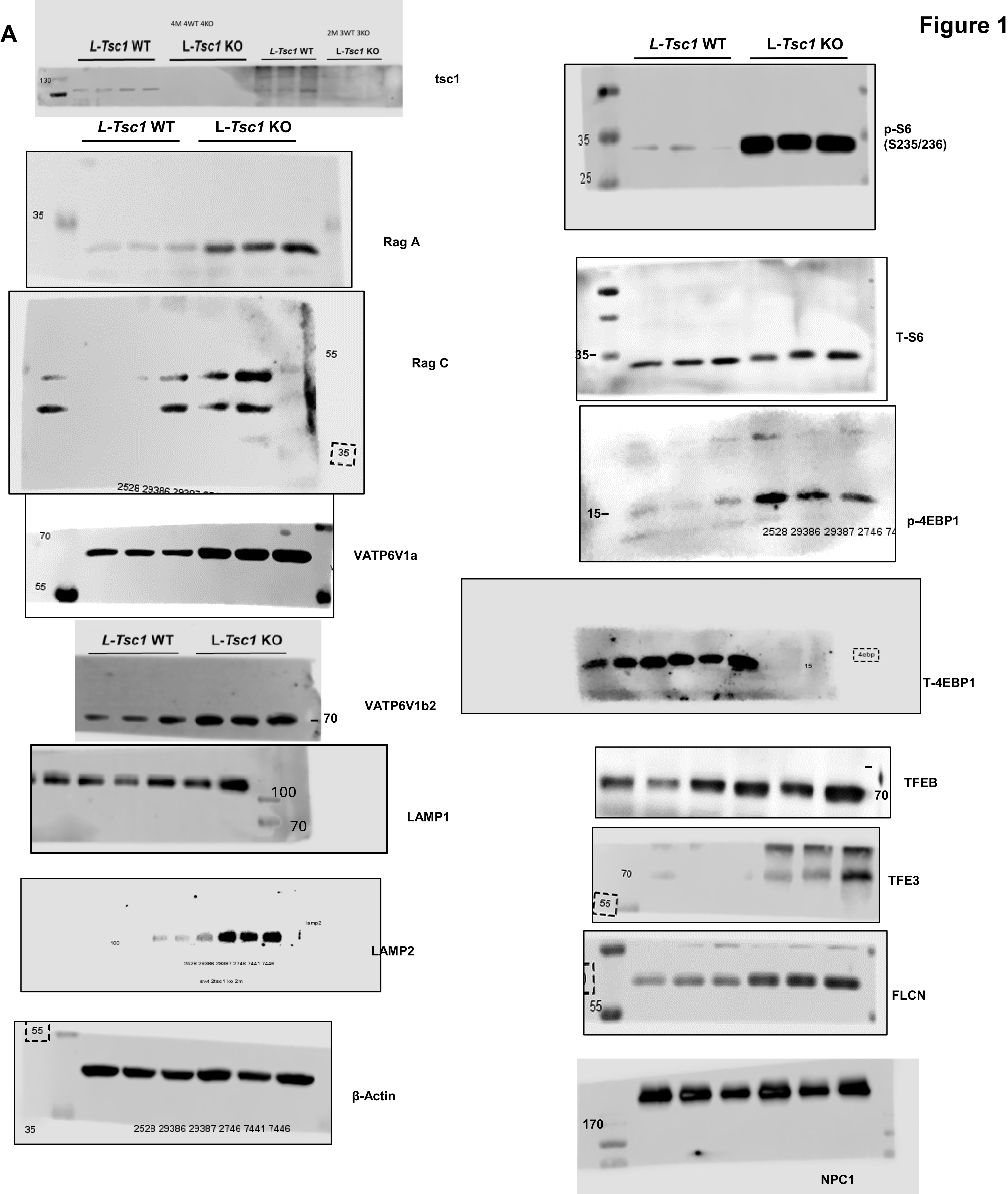
A
4M 4WT 4KO
2M 3WT 3KO
L-Tsc1 WT
L-Tsc1 KO
tsc1
130
Rag A
35
Rag C
55
35
2528 29386
VATP6V1a
70
55
VATP6V1b2
70
LAMP1
100
70
LAMP2
100
lamp2
2528 29386 29387 2746 7441 7446
β-Actin
55
35
2528 29386 29387 2746 7441 7446
p-S6 (S235/236)
35
25
T-S6
35
p-4EBP1
15
2528 29386 29387 2746
T-4EBP1
4ebp
15
TFEB
70
TFE3
70
55
FLCN
55
NPC1
170

Figure 2

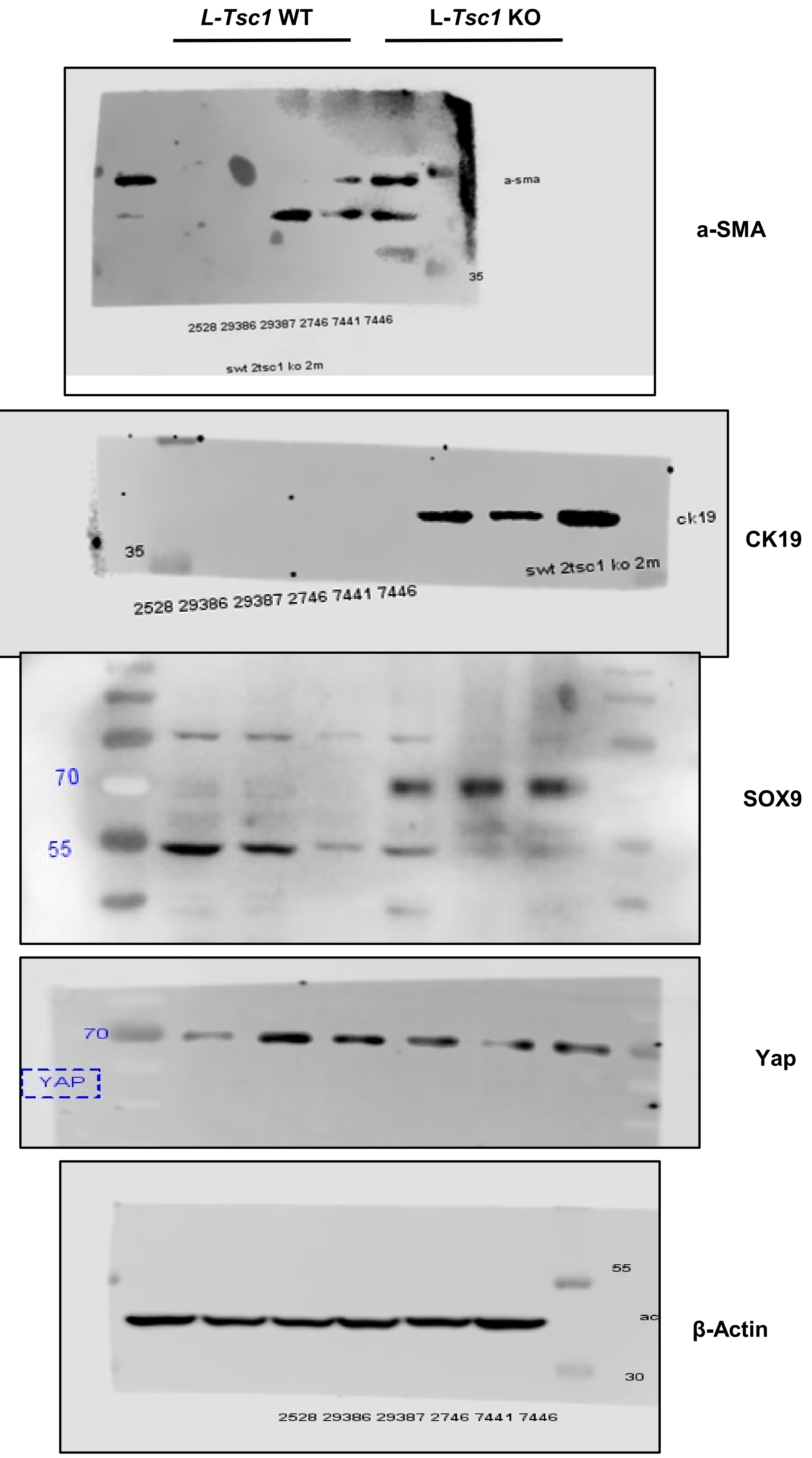
L-Tsc1 WT
L-Tsc1 KO
a-sma
35
2528 29386 29387 2746 7441 7446
swt 2tsc1 ko 2m
a-SMA
ck19
35
swt 2tsc1 ko 2m
2528 29386 29387 2746 7441 7446
CK19
70
55
SOX9
70
YAP
Yap
55
30
2528 29386 29387 2746 7441 7446
β-Actin

Figure 2

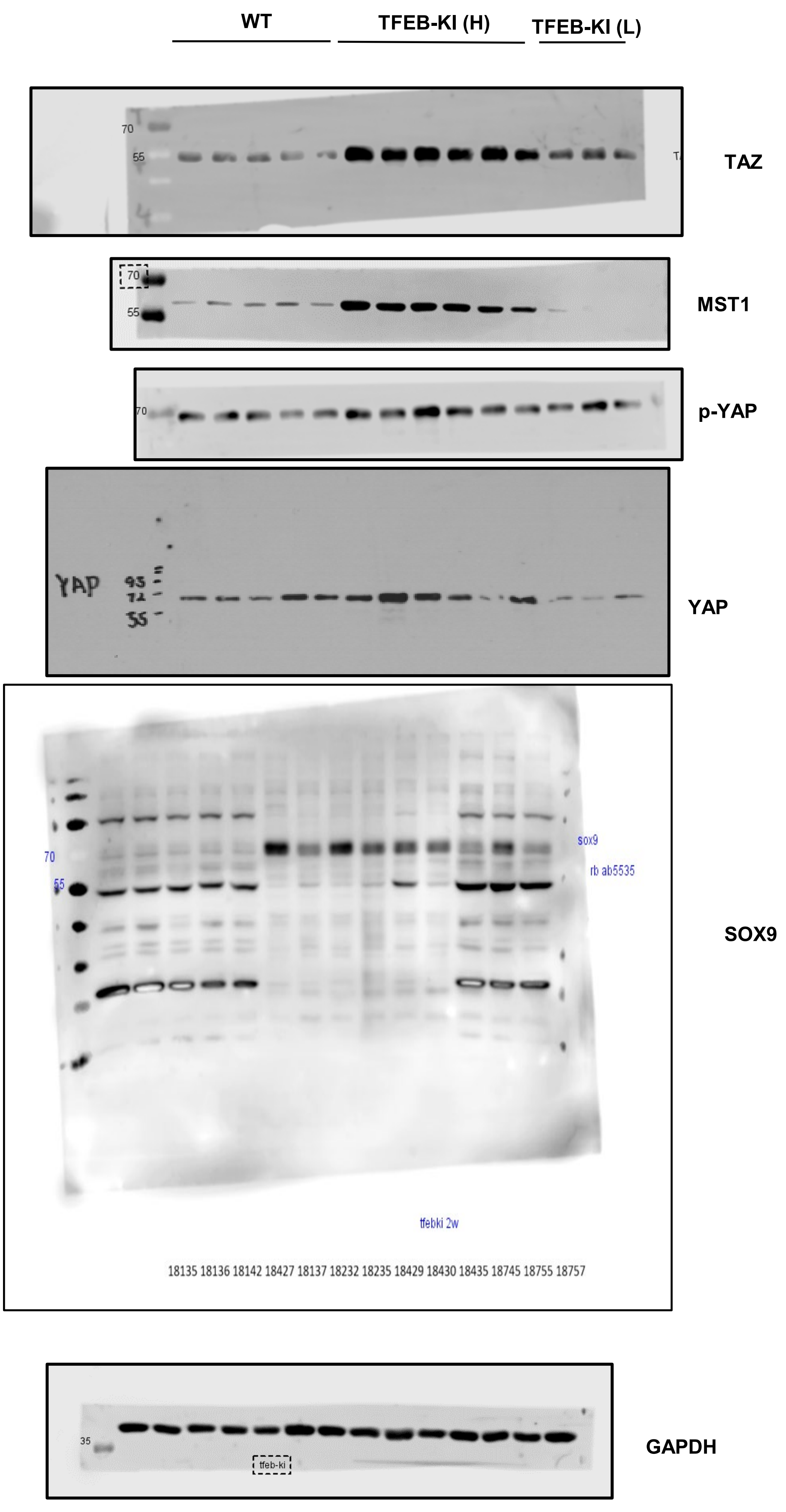

WT
TFEB-KI (H)
TFEB-KI (L)
70
55
TAZ
70
55
MST1
70
p-YAP
YAP
95
72
55
YAP
70
55
sox9
rb ab5535
SOX9
tfebki 2w
18135 18136 18142 18427 18137 18232 18235 18429 18430 18435 18745 18755 18757
35
tfeb-ki
GAPDH

Figure 3

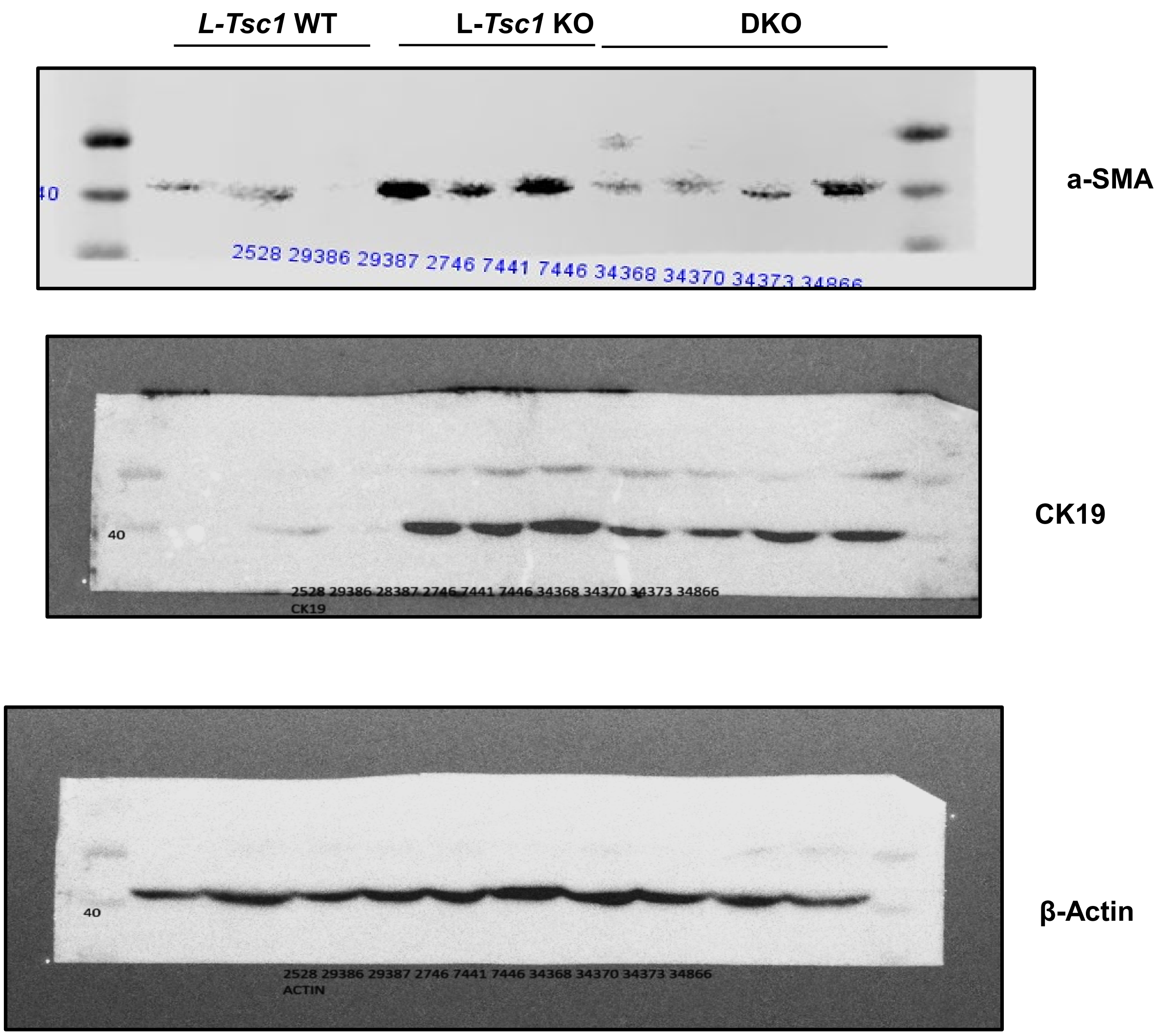

L-Tsc1 WT
L-Tsc1 KO
DKO
40
2528 29386 29387 2746 7441 7446 34368 34370 34373 34866
a-SMA
40
2528 29386 28387 2746 7441 7446 34368 34370 34373 34866
CK19
CK19
40
2528 29386 29387 2746 7441 7446 34368 34370 34373 34866
ACTIN
β-Actin

sFigure 1

LAMP1

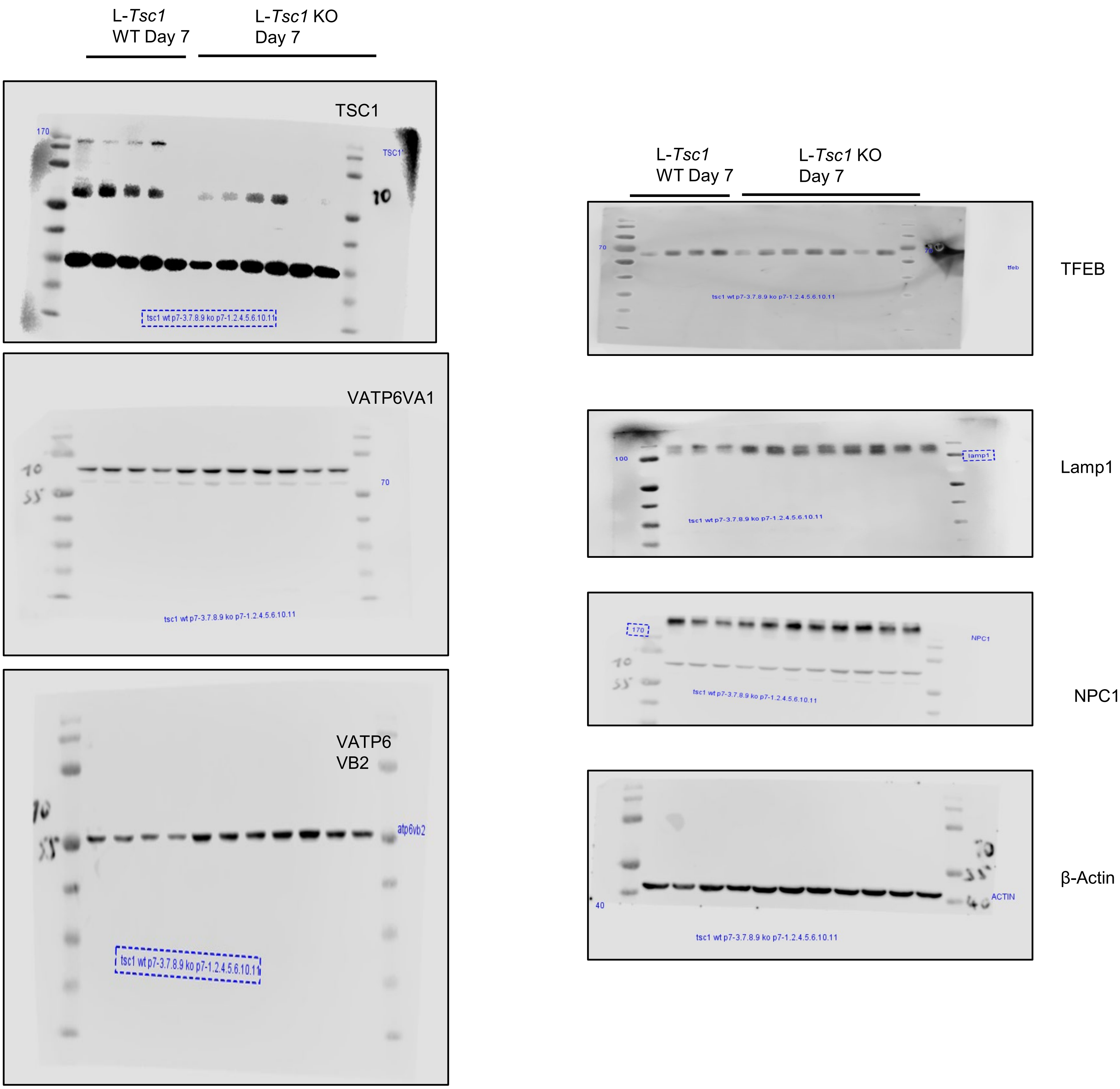

sFigure 3

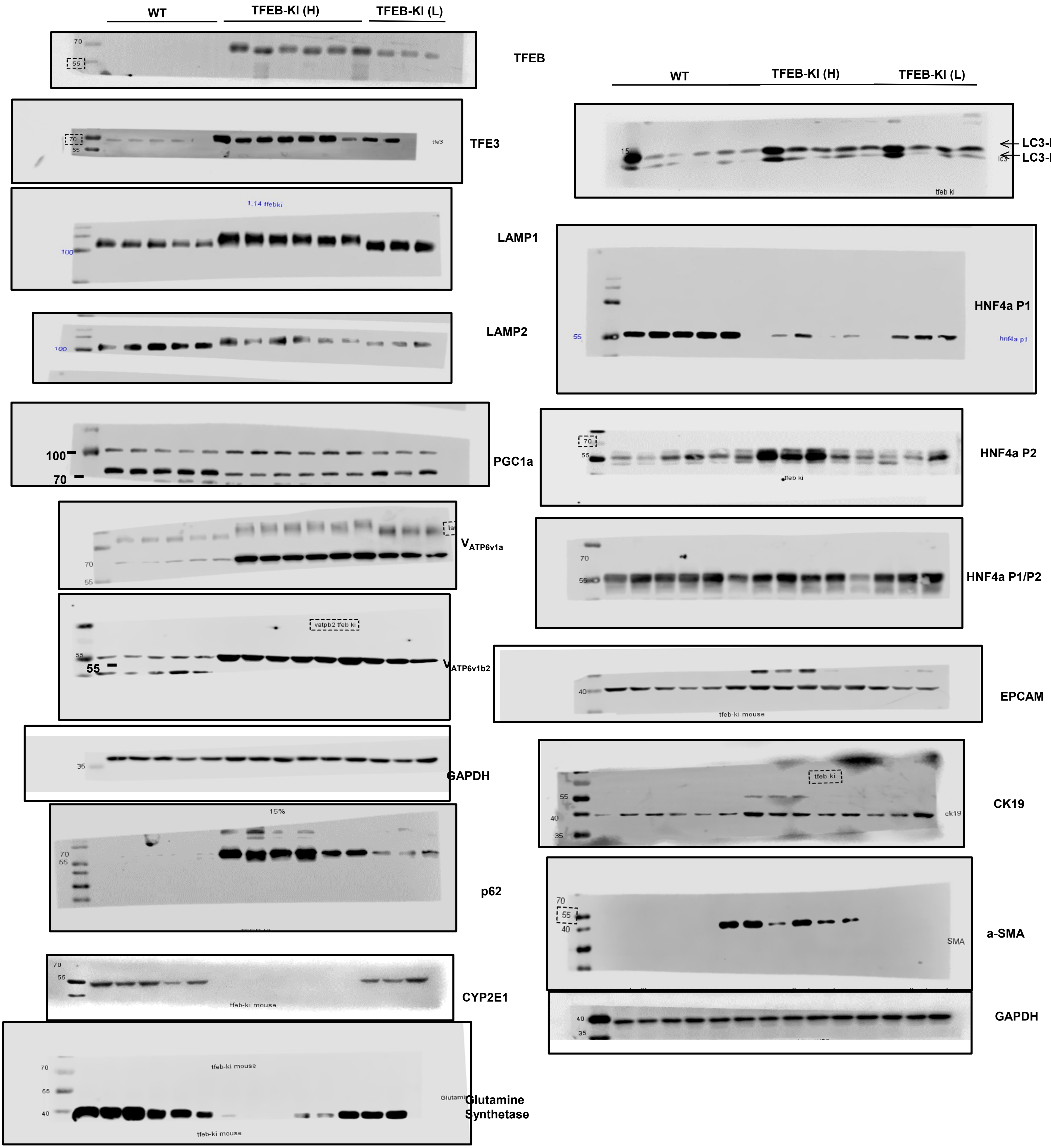

WT
TFEB-KI (H)
TFEB-KI (L)
TFEB
TFE3
1.14 tfebki
LAMP1
LAMP2
100
70
PGC1a
VATP6v1a
vatpb2 tfeb ki
55
VATP6v1b2
GAPDH
15%
p62
CYP2E1
tfeb-ki mouse
Glutamine Synthetase
LC3-I
LC3-II
tfeb ki
HNF4a P1
hnf4a p1
HNF4a P2
HNF4a P1/P2
EPCAM
tfeb-ki mouse
CK19
ck19
a-SMA
SMA
GAPDH

sFigure 6

C

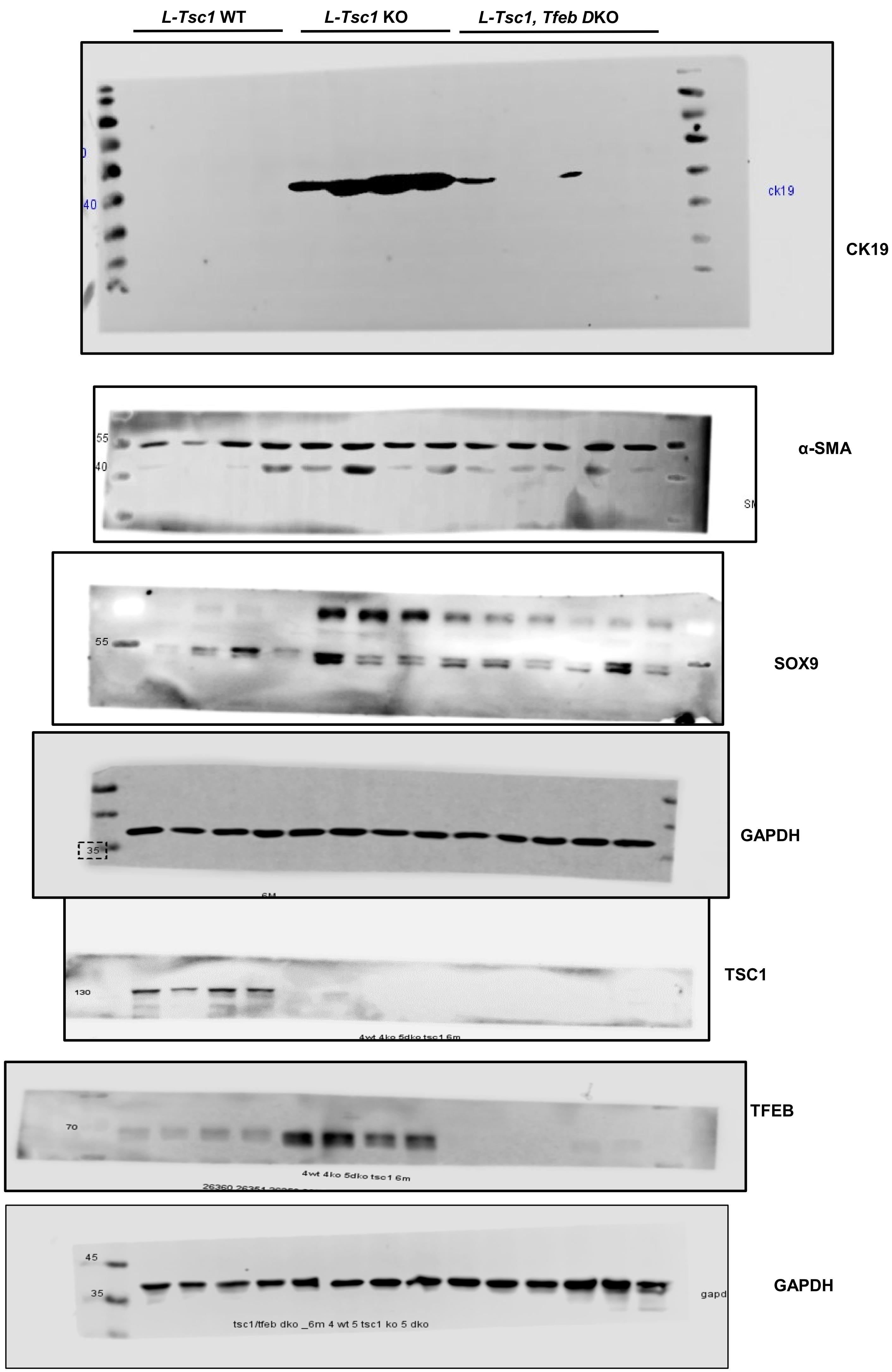
L-Tsc1 WT
L-Tsc1 KO
L-Tsc1, Tfeb DKO
40
ck19
CK19
55
40
α-SMA
55
SOX9
35
GAPDH
130
4wt 4ko 5dko tsc1 6m
TSC1
70
4wt 4ko 5dko tsc1 6m
TFEB
45
35
tsc1/tfeb dko _6m 4 wt 5 tsc1 ko 5 dko
gapd
GAPDH

sFigure 7

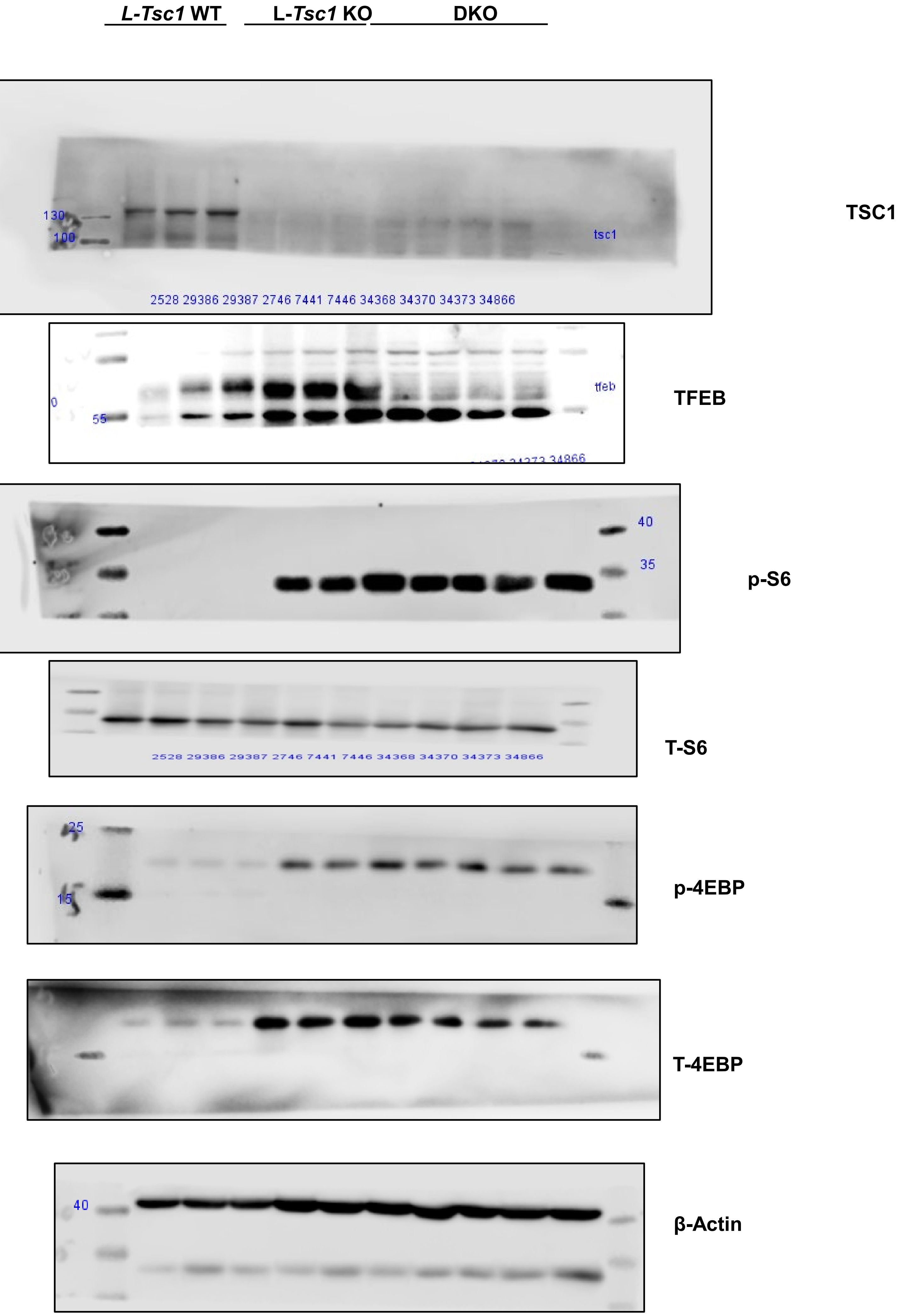

L-Tsc1 WT
L-Tsc1 KO
DKO
130
100
tsc1
2528 29386 29387 2746 7441 7446 34368 34370 34373 34866
TSC1
tfeb
55
TFEB
40
35
p-S6
2528 29386 29387 2746 7441 7446 34368 34370 34373 34866
T-S6
25
15
p-4EBP
T-4EBP
40
β-Actin

sFigure 7

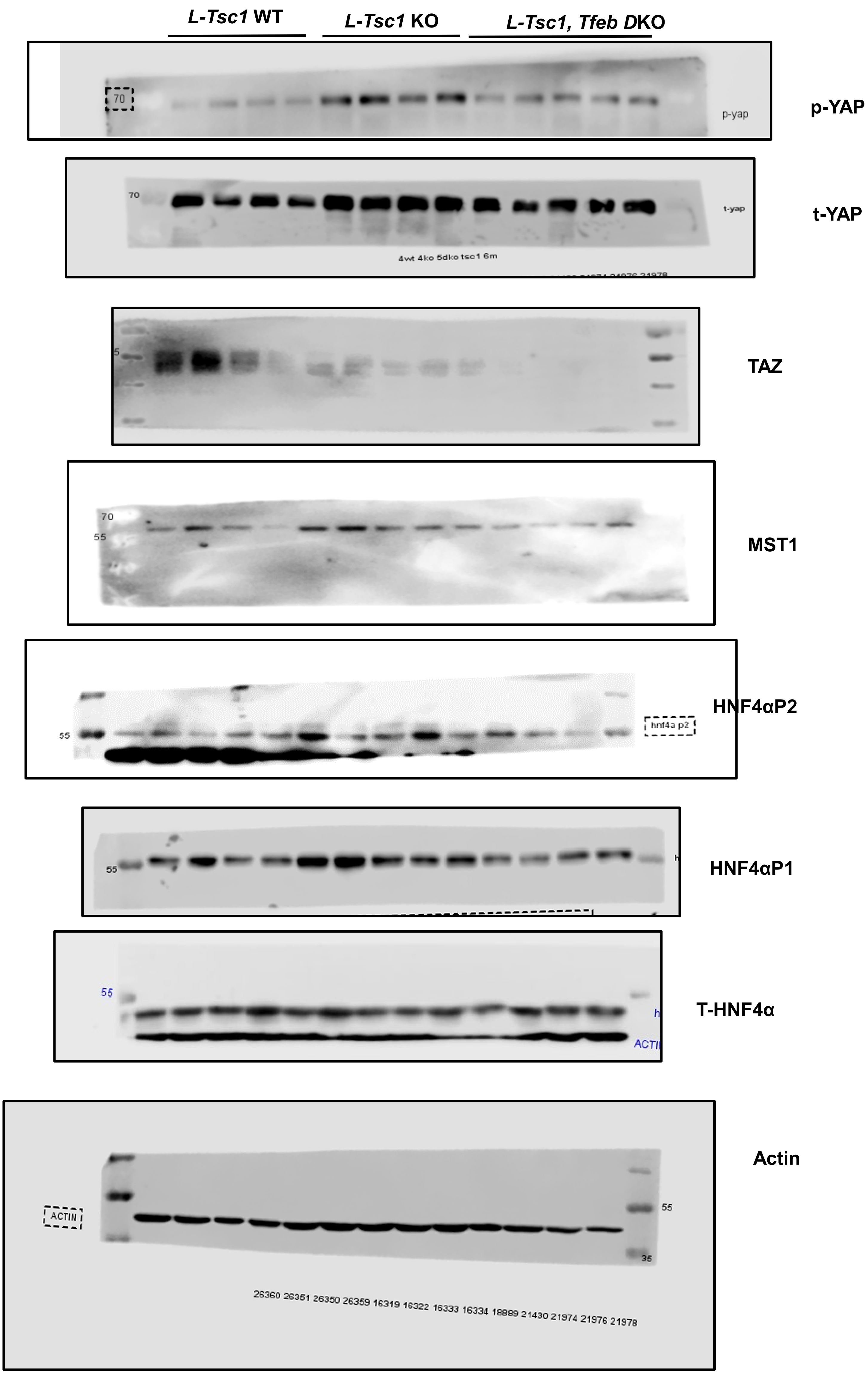
L-Tsc1 WT
L-Tsc1 KO
L-Tsc1, Tfeb DKO
70
p-yap
p-YAP
70
t-yap
t-YAP
4wt 4ko 5dko tsc1 6m
TAZ
70
55
MST1
55
hnf4a p2
HNF4αP2
55
HNF4αP1
55
ACTI
T-HNF4α
ACTIN
55
35
Actin
26360 26351 26350 26359 16319 16322 16333 16334 18889 21430 21974 21976 21978

sFigure 8

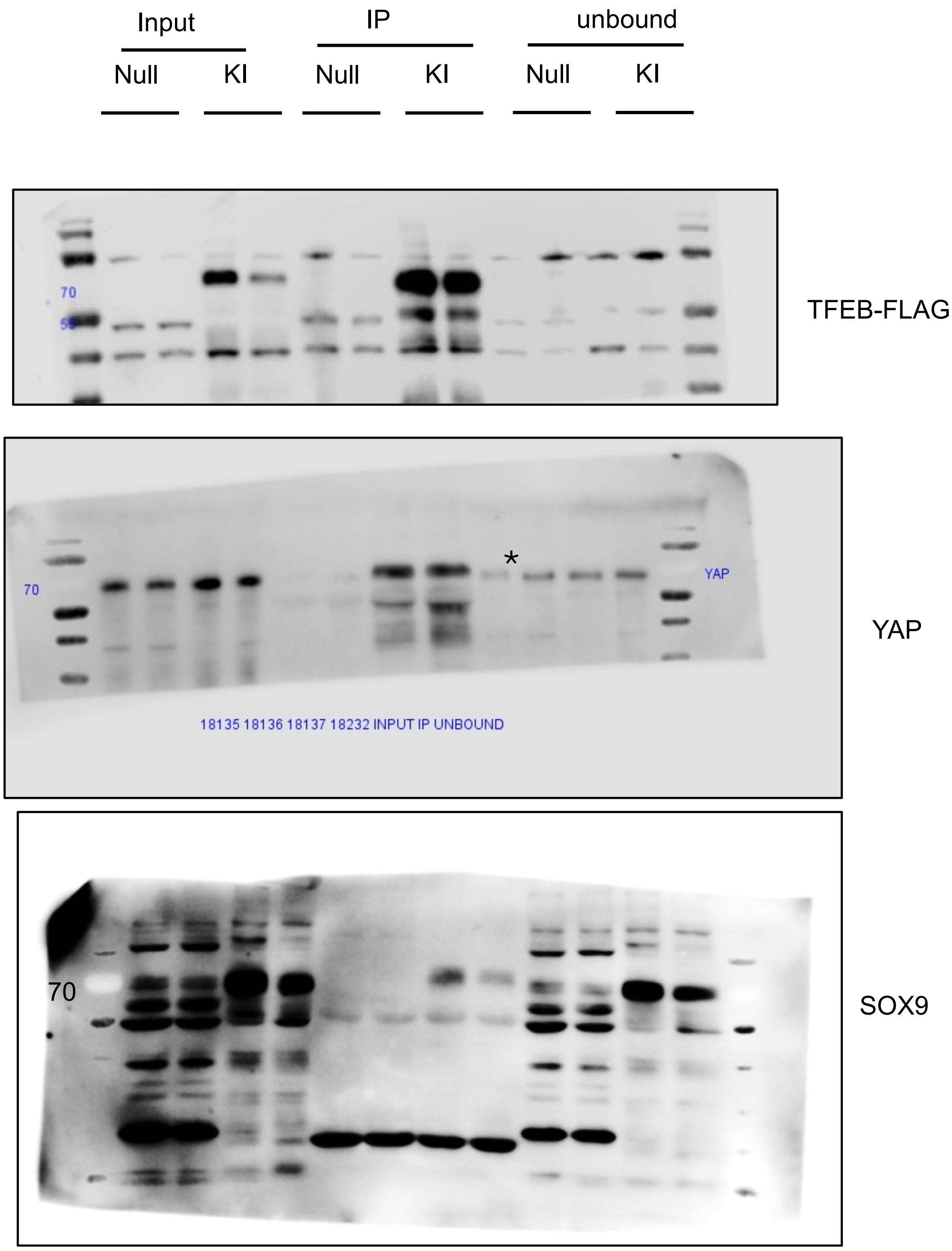
Input
IP
unbound
Null
KI
Null
KI
Null
KI
70
TFEB-FLAG
70
*
YAP
18135 18136 18137 18232 INPUT IP UNBOUND
YAP
70
SOX9

sFigure 9

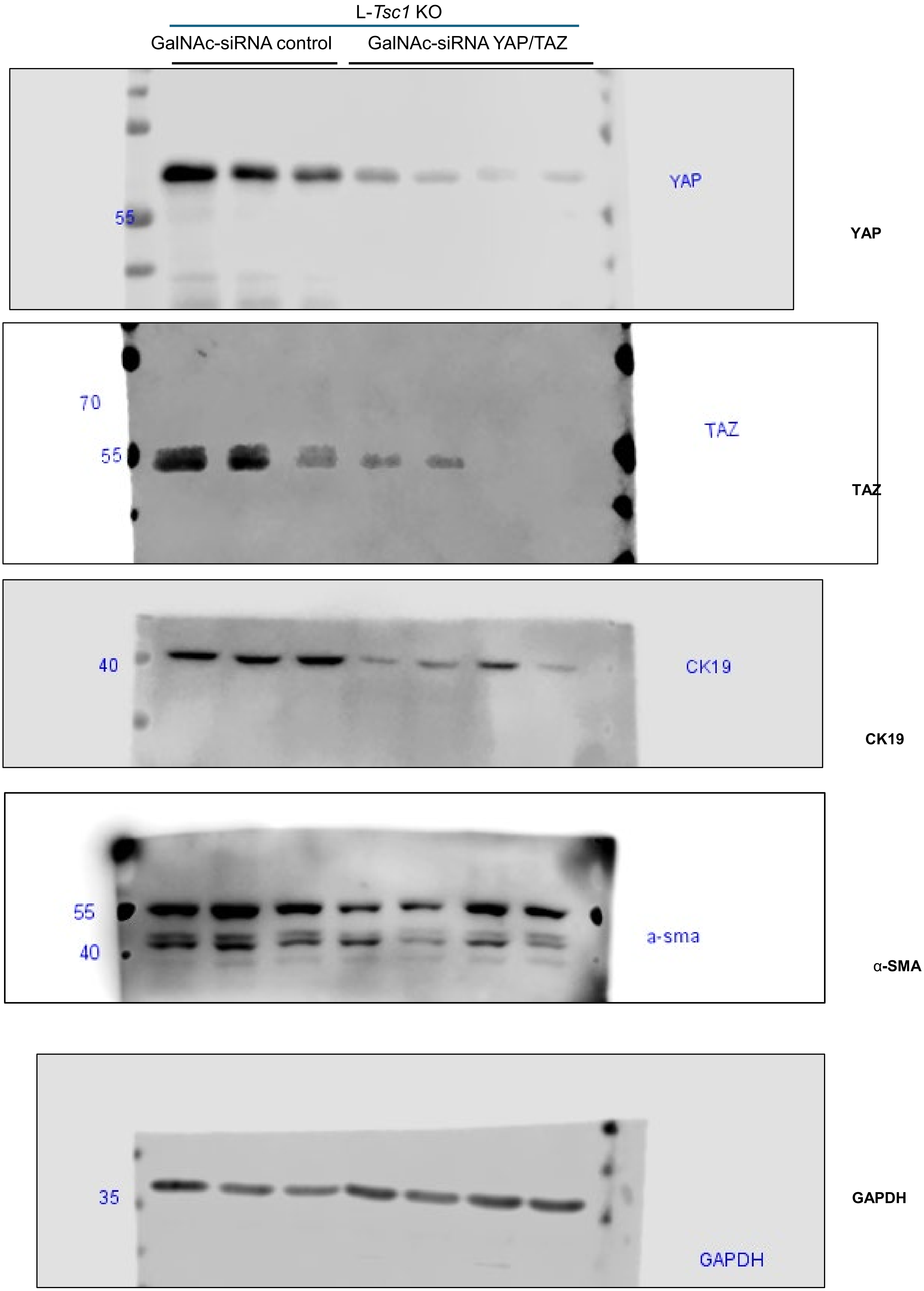

L-Tsc1 KO
GalNAc-siRNA control
GalNAc-siRNA YAP/TAZ
55
YAP
YAP
70
55
TAZ
TAZ
40
CK19
CK19
55
40
a-sma
α-SMA
35
GAPDH
GAPDH

sFigure 12

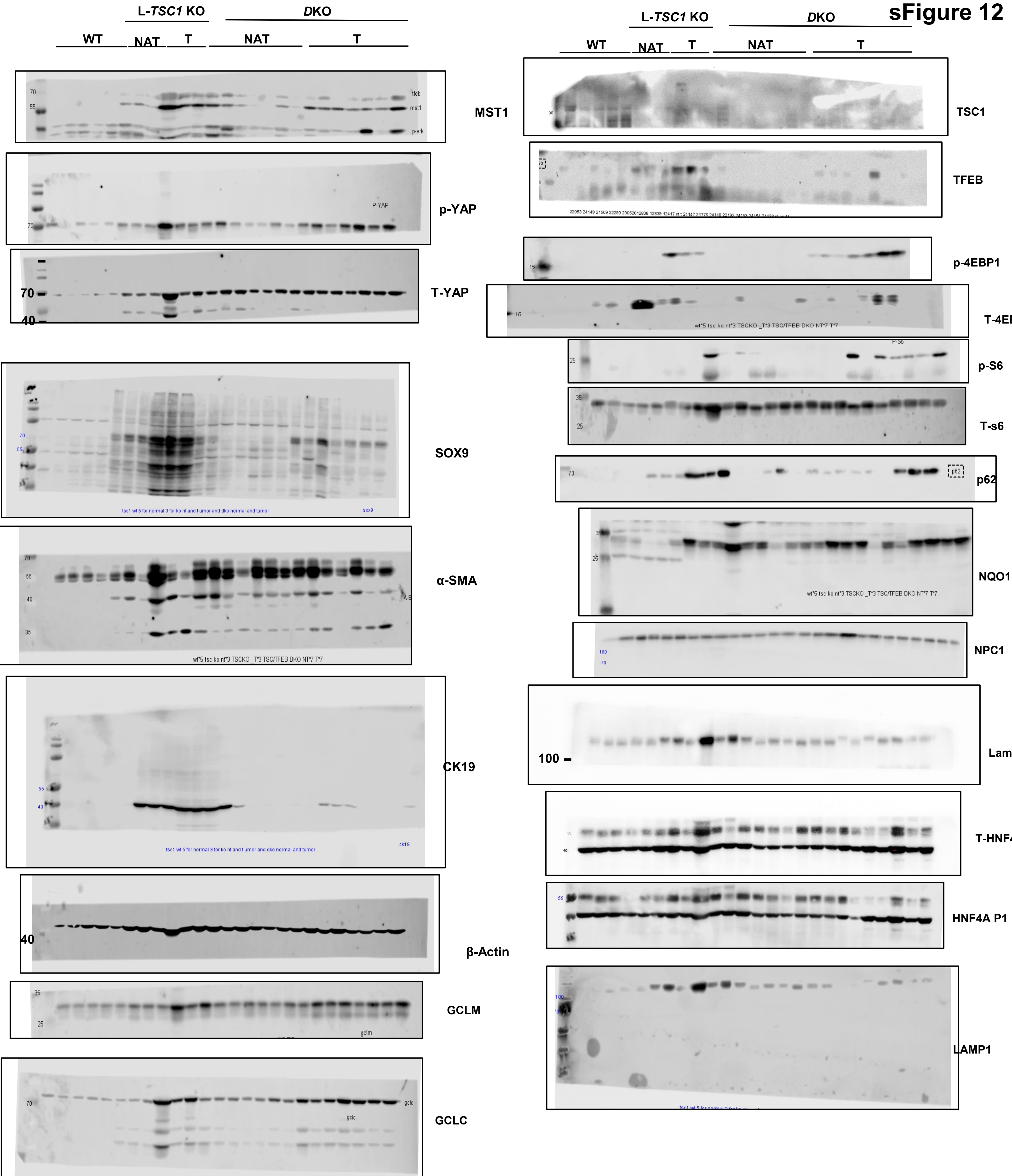
L-TSC1 KO
DKO
WT
NAT
T
MST1
p-YAP
T-YAP
SOX9
α-SMA
CK19
β-Actin
GCLM
GCLC
TSC1
TFEB
p-4EBP1
T-4E
p-S6
T-s6
p62
NQO1
NPC1
Lam
T-HNF
HNF4A P1
LAMP1

sFigure 12

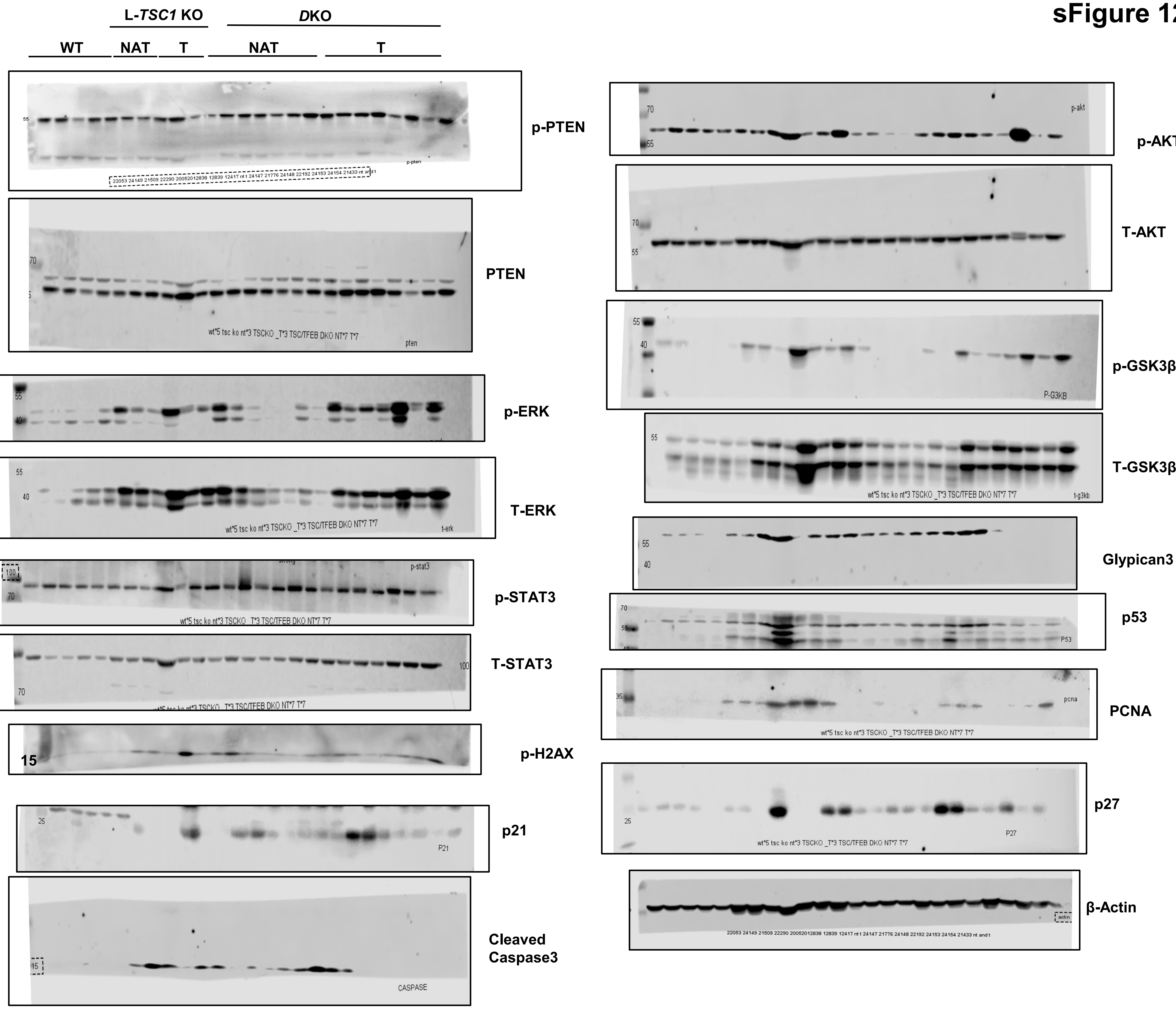
L-TSC1 KO
DKO
WT
NAT
T
NAT
T
p-PTEN
PTEN
p-ERK
T-ERK
p-STAT3
T-STAT3
p-H2AX
p21
Cleaved Caspase3
p-AKT
T-AKT
p-GSK3β
T-GSK3β
Glypican3
p53
PCNA
p27
β-Actin

sFigure 13

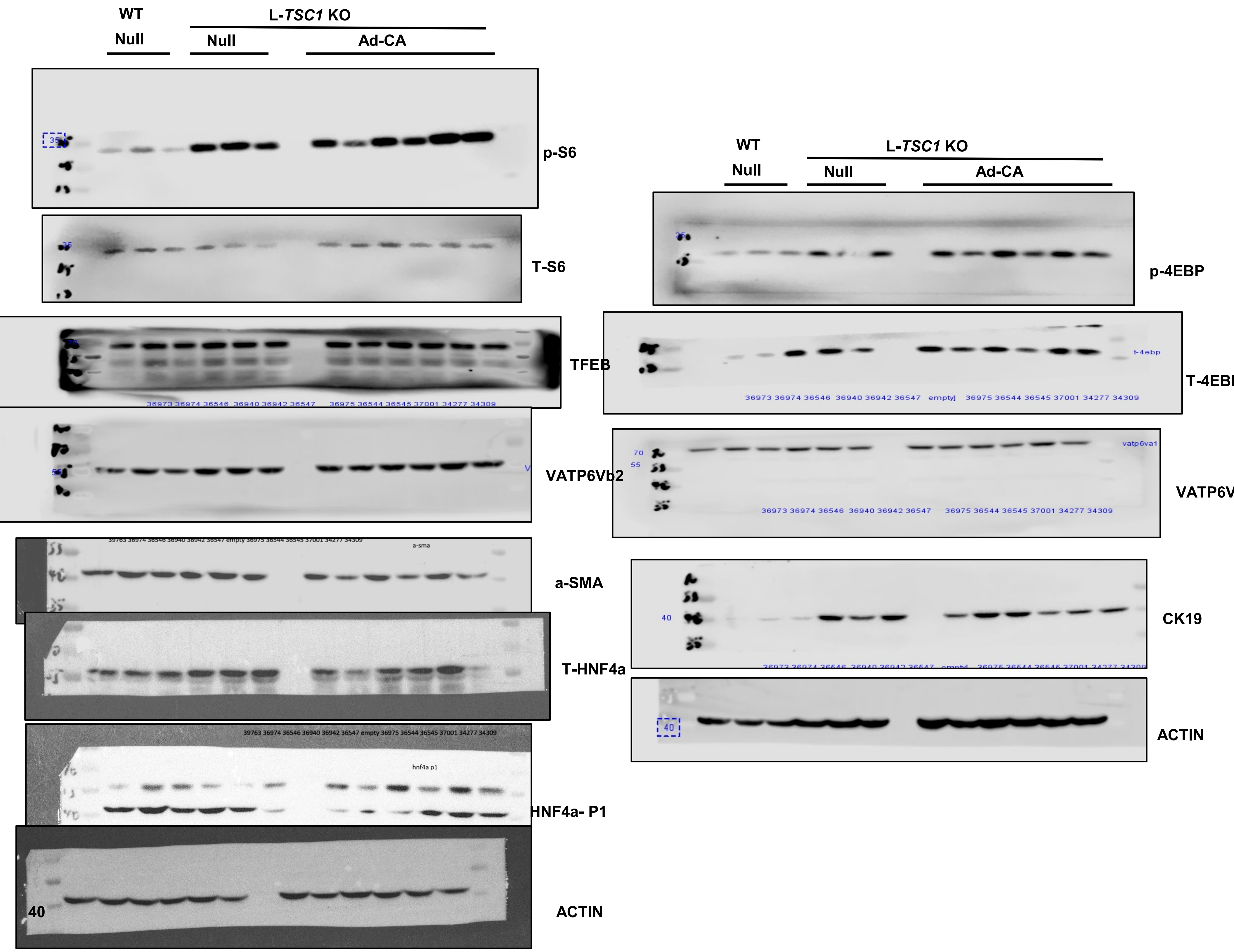

WT
Null
L-TSC1 KO
Null
Ad-CA
p-S6
T-S6
TFEB
VATP6Vb2
a-SMA
T-HNF4a
HNF4a- P1
ACTIN
WT
Null
L-TSC1 KO
Null
Ad-CA
p-4EBP
T-4EBP
VATP6V
CK19
ACTIN
